# Monitoring Mycotoxin Exposure in Food-Producing Animals (Cattle, Pig, Poultry, and Sheep)

**DOI:** 10.3390/toxins16050218

**Published:** 2024-05-09

**Authors:** Borja Muñoz-Solano, Elena Lizarraga Pérez, Elena González-Peñas

**Affiliations:** Department of Pharmaceutical Sciences, Faculty of Pharmacy and Nutrition, Universidad de Navarra, 31008 Pamplona, Spain; bmunoz.1@alumni.unav.es (B.M.-S.); elizarraga@unav.es (E.L.P.)

**Keywords:** food-producing animals, biomarker, animal biomonitoring, exposure, feed, mycotoxins

## Abstract

Food-producing animals are exposed to mycotoxins through ingestion, inhalation, or dermal contact with contaminated materials. This exposure can lead to serious consequences for animal health, affects the cost and quality of livestock production, and can even impact human health through foods of animal origin. Therefore, controlling mycotoxin exposure in animals is of utmost importance. A systematic literature search was conducted in this study to retrieve the results of monitoring exposure to mycotoxins in food-producing animals over the last five years (2019–2023), considering both external exposure (analysis of feed) and internal exposure (analysis of biomarkers in biological matrices). The most commonly used analytical technique for both approaches is LC-MS/MS due to its capability for multidetection. Several mycotoxins, especially those that are regulated (ochratoxin A, zearalenone, deoxynivalenol, aflatoxins, fumonisins, T-2, and HT-2), along with some emerging mycotoxins (sterigmatocystin, nivalenol, beauvericin, enniantins among others), were studied in 13,818 feed samples worldwide and were typically detected at low levels, although they occasionally exceeded regulatory levels. The occurrence of multiple exposure is widespread. Regarding animal biomonitoring, the primary objective of the studies retrieved was to study mycotoxin metabolism after toxin administration. Some compounds have been suggested as biomarkers of exposure in the plasma, urine, and feces of animal species such as pigs and poultry. However, further research is required, including many other mycotoxins and animal species, such as cattle and sheep.

## 1. Introduction

Some genera of filamentous fungi, especially *Aspergillus*, *Penicillium*, and *Fusarium*, are capable of producing natural contaminants called mycotoxins, which have toxic effects on humans and animals. These compounds are considered as more hazardous to human and animal health than other food contaminants, such as pesticides, preservatives, or food additives [1,2]. For this reason, there is very active research on these compounds, and the European Union (EU) considers mycotoxins as a group of priority substances [3].

Since their discovery in the 1960s, several hundred mycotoxins have been discovered [4,5] (some examples can be seen in Table 1). These compounds contaminate different raw materials worldwide, such as cereals, nuts, fruits, dried fruits, spices, coffee, etc., with cereals being one of the main sources of exposure to mycotoxins; in fact, contamination can be present in up to 60–80% of the food crops samples analyzed, as indicated by Eskola et al. (2020) [6].

Mycotoxins have very different physicochemical characteristics but generally share high stability in relation to temperature and resistance to physical and/or chemical treatments intended for their elimination from contaminated materials; once present in a product, their removal is extremely difficult [7,8].

**Table 1:** Fungi and their main mycotoxins.

Fungi	Mycotoxin	Ref.
*Fusarium*	DON, T-2, HT-2, DAS, NEO, NIV, 3-ADON, 15-ADON, FUS-X, FB1, FB2, FB3, ZEA, MON, ENNs, BEA	[7]
*Aspergillus*	AFB1, AFB2, AFG1, AFG2, OTA, FB2, FB4, STER, PAT	[9]
*Penicillium*	OTA, PAT, ROQC, CIT, cyclopiazonic acid	[10]

AFB1: aflatoxin B1; AFB2: aflatoxin B2; AFG1: aflatoxin G1; AFG2: aflatoxin G2; BEA: beauvericin; CIT: citrinin; DAS: diacetoxyscirpenol; DON: deoxynivalenol; 3-ADON: 3-acetyl deoxynivalenol; 15-ADON: 15-acetyl deoxynivalenol; ENNs: enniatins; FB1: fumonisin B1; FB2: fumonisin B2; FB3: fumonisin B3; FB4: fumonisin B4; FUS-X: fusarenon-X; HT-2: HT-2 toxin; MON: moniliformin; NEO: neosolaniol; NIV: nivalenol; OTA: ochratoxin A; PAT: patulin; ROQC: roquefortine C; STER: sterigmatocystin; T-2: T-2 toxin; ZEA: zearalenone.

To prevent fungal and mycotoxin contamination, good practices should be followed throughout the entire process, from the field to the table [7]. Additionally, regulations in different regions around the world (a summary of which can be found in [6,11]) aim to prevent and limit the presence of these toxins in food crops because mycotoxin levels above the maximum legislated levels, such as those set by the EU, are considered unsafe [12]; however, low concentrations of mycotoxins in the diet can lead to chronic toxicity [8].

Currently, some factors could disrupt the typical global distribution of mycotoxin contamination. Firstly, fungal infestation heavily depends on climatic conditions (temperature and humidity) and there is insufficient understanding of how global warming and changes in precipitation patterns due to climate change will affect food security in general [13] and mycotoxin distribution in particular. The EU MycoKey project concluded that climate change is increasing the prevalence of mycotoxins in food and feed [14]. Moreover, if ecosystems change and crops have to grow in unsuitable climate conditions (pollution, nutrient deficiencies, plant damage by insects or pests, etc.), stressed plants will be more susceptible to fungal contamination [2,3,15,16]. 

Secondly, the global trade of raw materials has increased, with most countries utilizing raw materials produced in different regions of the world [11], each with varying climatic conditions, agricultural and storage procedures, or legislation regarding mycotoxin contamination limits. All of the above underscore the challenge and necessity of mycotoxin control.

Mycotoxins reach animals via different routes. The most significant route is ingestion through feed and/or forage [3]. However, exposure through skin contact or aerosols has been demonstrated in farm staff [17], with varying levels of aflatoxin B1 (AFB1), deoxynivalenol (DON) conjugates, aflatoxin M1 (AFM1), enniatin B (ENNB), citrinin (CIT), dihydrocitrinone (DH-CIT), and ochratoxin A (OTA) found in the biological fluids of swine production workers. This was due to the presence of fungi in litter and feed samples, the generation of dust and aerosols during normal farm work, and the confinement in which animals are kept [18,19]. Therefore, if humans are exposed to mycotoxins in the farm environment through dermal or airborne contact, it can be assumed that animals will also be exposed to them. 

Mycotoxins in animals often cause structural and functional damage to the liver, nephrotoxicity, poor growth weight (due to vomiting, diarrhea, loss of appetite, etc.), poor productivity (due to decreased milk or egg production and lower quality), susceptibility to diseases (due to immunosuppressive effects), dehydration, weakness, death, respiratory infections and pulmonary edema, and reproductive problems, such as hypoestrogenism, sterility, and abortions, etc. [2,20,21]. The effects vary depending on the mycotoxin, contamination level, animal health status, and animal species. For instance, ruminants appear to be less sensitive than poultry and pigs [2], and pigs are severely affected by mycotoxins [22]. 

Moreover, mycotoxins can contaminate foods derived from contaminated animals, such as meat, milk, or eggs, reaching the human food chain and also causing impairment of the health status of consumers [15]. The symptoms of mycotoxicosis in humans are quite similar and dangerous to those in animals: carcinogenicity, organ damage, impairment to the endocrine, reproductive, or immune system, allergenicity, etc. [23,24]. The International Agency for Research on Cancer (IARC) has evaluated and classified some mycotoxins concerning their carcinogenicity to humans into three groups: Group 1 (carcinogenic to humans), which includes aflatoxins (AFs), especially AFB1; Group 2B (possibly carcinogenic to humans), which includes fumonisins (FBs), OTA, and sterigmatocystin (STER); and finally, Group 3 (not classifiable), in which most mycotoxins are included, mainly because little information was available at the time of the IARC evaluation. This group includes zearalenone (ZEA), DON, nivalenol (NIV), T-2 and HT-2 toxins, CIT, and patulin (PAT). Others, such as enniatins (ENNs) or beauvericin (BEA), have not been evaluated [23,25,26,27]. 

Mycotoxins not only pose food safety problems but also have significant economic implications for the livestock sector, which is vital to the global economy [28]. Additionally, increasing demand for livestock products is expected in order to feed a growing world population [20]. The presence of mycotoxins in raw materials and feeds imposes a high cost for farmers and food importers. This is due to the need to test crops for mycotoxins to comply with regulations, the losses of contaminated batches, damage to the reputation of companies or exporting countries [29], reduced food production, the cost associated with treating animal mycotoxicosis, and even the loss of animals due to illness [30]. For instance, in the USA, aflatoxin contamination in corn is estimated to result in losses ranging from USD 52.1 million to USD 1.68 billion annually [31]. 

Finally, another important consideration is that when raw materials or feeds are contaminated with mycotoxins, the concurrent presence of several of them is the most common scenario. This can occur due to several factors: multiple fungal species contaminating the same product, a single fungal species producing more than one mycotoxin, and feeds being prepared using various raw materials [20,32]. These co-occurring mycotoxins can have additive, antagonistic, or synergistic effects [30,33], and this aspect is not fully understood, despite ongoing research [34,35]. The authors emphasize that this is an issue that warrants serious consideration.

From the above, it can be inferred that mycotoxin contamination of raw materials and feed is becoming increasingly significant, with mycotoxins posing one of the most significant hazards worldwide [6] due to their widespread presence and their implications for economic, animal, and human health.

The main objective of this work is to contribute to the control of mycotoxin exposure in food-producing animals, particularly in cattle, pigs, poultry, and sheep, given their critical role in the livestock sector and economy of Europe [28]. To achieve this, a systematic review is conducted here to recover the results of monitoring mycotoxins in food-producing animals over the last five years.

## 2. Monitoring Mycotoxin Exposure in Food-Producing Animals

Two of the essential elements for the necessary control of mycotoxin contamination are monitoring and surveillance actions [36], since they provide data that enable us (a) to assess the exposure of animals and humans, (b) to guarantee the quality and safety of raw materials and their derivatives before use, ensuring they do not pose any danger to human health, animal health, or livestock production, (c) to assess risks to animal and human health, and (d) to ensure regulatory compliance [36,37,38]. Furthermore, these actions must be continuously carried out due to changes in contamination levels over time and in the different raw materials that may result from the factors indicated above.

Compound feed (hereafter, feed) is typically prepared from cereals, which are the most important feed components and, also, raw materials prone to fungal and mycotoxin contamination [39]. Consequently, the predominant approach to studying the exposure of animals to mycotoxins has been the analysis of the presence of these compounds in raw materials or feed. These data are then combined with existing information on dietary intake. In the EU, the European Food Safety Authority (EFSA) Panel on Contaminants in the Food Chain evaluates the risks to human and animal health from various hazardous compounds, including mycotoxins, present in food and/or feed. Where possible, the EFSA establishes a tolerable daily intake for a substance and provides data for setting the maximum levels of contaminants in food and feed or suggests monitoring measures related to the presence of contaminants. An example can be seen in the EFSA Risk Assessment of ergot alkaloids in feed [40]. This approach is referred to as external exposure assessment [41]. 

This approach is very useful in some aspects—for example, it allows for the development of knowledge on the level of mycotoxin contamination in raw materials and feeds, along with the development of appropriate strategies to prevent the presence of mycotoxin in these matrices. 

In March 2024, the EFSA published a call for the collection of data on the occurrence of contaminants in food and feed to be used in the preparation of EFSA scientific opinions and reports. BEA and ENNs are mycotoxins on the priority list because scientific opinions are being prepared. Other mycotoxins for which data are needed included AFB1 in feed, OTA, DON and 3 and 15-acetyldeoxynivalenol (3- and 15-ADON), DON 3-glucoside (DON-3gluc), ZEA, its derivatives and modified forms, FBs and their modified forms, alternariol (AOH), alternariol monomethyl ether (AME), tenuazonic acid, tentoxin (TENT), T-2 and HT-2 and their modified forms, NIV and its modified forms, ergot alkaloids (ERGOT), ENNs, STER, BEA, CIT, moniliformin (MON), diacetoxyscirpenol (DAS), and phomopsins in food and feed [42]. 

However, this approach also has some drawbacks. In the case of food-producing animals, (a) there are different species of interest with varying diet composition, feed intake levels, absorption rates, distribution patterns, elimination processes, or sensitivity to these toxins, which vary not only between species but also among individuals within the same species [43,44], and this information is typically not available; (b) there is a lack of adequate databases on feed consumption [40]; (c) mycotoxins may not be homogeneously distributed in raw materials or feed, making it difficult to obtain a representative sample [45]; and finally, (d) in the samples to be analyzed, mycotoxins can undergo chemical modifications as a result of metabolism by plants or other competing fungi, or they may become bound to matrix components; in such cases, they would not be detected using analytical methods designed for the analysis of parent compounds [7,46,47,48]. 

The second and more recent approach is the assessment of the internal exposure of animals to mycotoxins. This is carried out by analyzing biomarkers in biological tissues or fluids. A biomarker of mycotoxin exposure can be defined as a molecule whose presence in a biological matrix can be correlated with mycotoxin exposure. The significant potential of this approach lies in its ability to assess exposure due to all possible routes (including dermal or inhalation), and not only through feed or forage ingestion. Additionally, it typically requires simple sample extraction in most cases and provides information on individual exposure; furthermore, it could offer a relationship between biomarker presence and toxic effects [3]. This approach is increasingly used in monitoring human exposure [18,43], known as human biomonitoring (HBM). Similarly, animal biomonitoring (ABM) could be as useful as HBM in assessing animal exposure [39]. 

Nevertheless, this approach also has its drawbacks. Firstly, it is necessary to determine good biomarkers for each of the toxins in each species and matrix, which is not an easy task. Most ABM studies use the parent compound, its conjugates (phase II metabolites), or its adducts with macromolecules as biomarkers of exposure [49]. However, selecting appropriate biomarkers should be based on studies on the metabolism and toxicokinetics of these compounds in different animal species. Appropriate analytical methods should be developed with good sensitivity (usually higher than that needed for toxins analysis in feed) and multianalyte capability and should be validated. For this purpose, biomarker reference standards must be available. Finally, the biological significance of levels found in biological fluids or tissues must be elucidated [43,50]. 

For all of the above, both approaches, external and internal exposure assessments, have their advantages and disadvantages. Neither is preferable; instead, they should be used as complementary methods [39,51] to understand the exposure of food-producing animals to mycotoxins and to obtain data for effective risk assessment.

This review considers both approaches, namely external exposure, focusing on studies analyzing feed for mycotoxins, and internal exposure, with the recovery of studies analyzing biomarkers of mycotoxin exposure in the biological matrices of food-producing animals.

## 3. External Exposure

**Figure 1:**
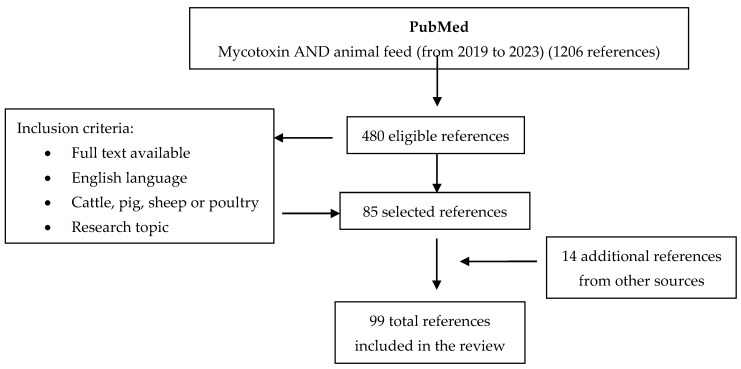
Flowchart of excluded and included studies based on the PRISMA Statement.

A systematic review based on the PRISMA statement [52] regarding the presence of mycotoxins in cattle, pig, poultry, and sheep feed is presented. In total, 99 articles were selected. The review strategy is described in Figure 1.

### 3.1. Analytical Methods

The development of analytical methods for quantifying mycotoxins in feed is very complex. Mycotoxins represent a heterogeneous group of chemical compounds, and their co-occurrence is very likely. Additionally, feed composition is highly variable due to feed being prepared with different raw materials and in diverse proportions. Furthermore, even within the same species, there are differences in composition driven by the need to meet specific nutritional requirements depending on the age of the animal, the season of the year, and other factors. However, due to the consequences of the consumption of contaminated feed, it is necessary to develop methods for their rapid detection and quantification. Preferably, these methods should enable the simultaneous analysis of several mycotoxins. Finally, these methods should utilize accessible, economical, and efficient technologies to facilitate their implementation in small companies and control laboratories [53].

The methods developed for the detection and quantification of mycotoxins in feed over the last five years (2019–2023) are described in Table S1.

Sample preparation plays a crucial role in this analysis due to the complexity of the matrix [54]. In fact, in all studies retrieved from the bibliography search, different clean-up steps, with their advantages and disadvantages, were required before analysis. Table S1 details the various methodologies employed to prepare feed samples in the last five years.

The first step in sample preparation involves extracting analytes from the feed using solid–liquid extraction (SLE) with various solvents. In some cases, this is the only clean-up step [54,55,56,57,58,59,60,61,62,63,64,65,66,67,68,69]. This methodology is characterized by its simplicity, speed, and cost-effectiveness, as well as its low selectivity, making it suitable for extracting several mycotoxins simultaneously. In fact, for some techniques, such as the enzyme-linked immunosorbent assay (ELISA) or the lateral flow immunochromatographic assay, SLE is the preferred choice [56,57,58,59,62,63,65]. Also, SLE is used to prepare samples before liquid chromatography-tandem mass spectrometry (LC-MS/MS) analysis and detection [54,60,61,64,66,67]. However, its main drawback is its low selectivity against matrix components, which may impact quantification [54]. Arroyo-Manzanares et al. (2019) [68] also used SLE before fluorescence detection (FLD). Different solvents have been employed, such as methanol (MeOH) [55,59,62,63,65,69], mixtures of MeOH/H_2_O [57,58] or MeOH/phosphate-buffered saline [56], H_2_O [57], acetonitrile (ACN) [68] and mixtures of ACN and H_2_O acidified using acetic acid [60,66] or formic acid [54,61,64,67] to facilitate the extraction of mycotoxins by breaking bonds between these compounds and proteins. 

In other cases, the extract after SLE may not be clear enough, necessitating a second cleanup step. Several authors have used immunoaffinity columns (IAC) before LC-FLD [70,71,72,73,74,75,76,77,78], LC-MS/MS [54,79,80], or LC coupled with an ultraviolet detector [73,77]. IAC-based clean-up is highly specific since these columns are composed of monoclonal antibodies against the mycotoxins of interest. This specificity allows for the removal of matrix components and other unwanted compounds in the extract, resulting in very clear extracts generally suitable for any type of liquid chromatography (LC) detector. This is advantageous when the aim is to analyze mycotoxins individually. However, the drawbacks of these methods include their high cost, the fact that they are usually designed for the analysis of a single or very few analytes, and the fact that there is a limited variety of columns available [79]. For these reasons, the simultaneous detection of multiple mycotoxins in routine analysis remains a challenge, and more research is needed to obtain new IAC columns capable of retaining multiple mycotoxins. For instance, Liu et al. (2022) [79] prepared a novel in-house multi-IAC for the extraction of six mycotoxins (DON, AFB1, ZEA, OTA, T-2 toxin, and FB1) in feed.

Solid phase extraction (SPE) is also employed after SLE [53,63,81,82,83,84,85]. It is a versatile clean-up technique that, depending on the chemical nature of the sorbent, removes or retains different compounds extracted from the matrix. This process enables cleaner samples to be obtained with fewer compounds that could co-elute and hinder analyte quantification [53] or generate matrix effects in the case of mass spectrometry-based detection. SPE has a lower selectivity than IAC, allowing for the simultaneous extraction of several mycotoxins. 

The QuEChERS (Quick, Easy, Cheap, Effective, Rugged, and Safe) method is also employed by [68,86,87,88]. This procedure involves one [68] or two steps [86,87,88]. The first consists of extracting mycotoxins using an ACN/H_2_O mixture acidified with formic acid and some salts (usually MgSO_4_ and NaCl) to achieve the separation of the ACN phase from the aqueous phase. Afterward, some authors improve the clean-up of the ACN extract by using solids for dispersive SPE, such as mixtures of two of the three following compounds: C18, primary/secondary amine (PSA), and MgSO_4_ [86,87,88]. 

Regarding the techniques for detecting and quantifying mycotoxins in animal feed, most authors used LC (on reversed-phase columns) coupled with mass spectrometry [54,60,61,64,66,67,68,79,80,81,83,84,85,86,87,88], employing electrospray ionization and triple quadrupoles (QqQs) or quadrupole ion traps (QTraps) as mass analyzers. In addition, LC coupled with FLD has been widely used [53,63,68,70,71,72,73,74,75,76,77,78,82], and, in some cases (for DON quantification), LC has been coupled with an ultraviolet detector [73,77]. 

Other methodologies have also been employed, such as ELISA [57,58,59,62,63,74], capillary electrophoresis-laser-induced fluorescence [55], or the lateral flow immunochromatographic assay [56]. These methods can facilitate the easy and rapid extraction of compounds from the matrix, as well as being economical techniques. However, they are being replaced by multidetection methods based on LC and MS/MS detectors.

**Figure 2:**
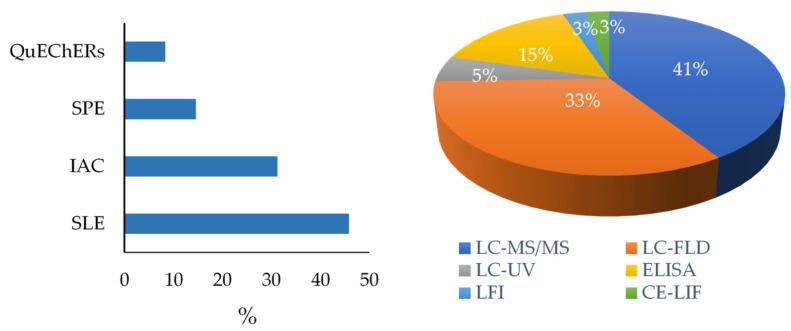
Percentage of articles using the different methodologies for mycotoxin analysis in feed in the retrieved publications (2019–2023). SPE: solid phase extraction, IAC: immunoaffinity columns, SLE: solid–liquid extraction, LFI: lateral flow immunochromatography, CE-LIF: capillary electrophoresis-laser-induced fluorescence, LC: liquid chromatography, MS: mass spectrometry, UV: ultraviolet detector, FLD: fluorescence detector.

The fundamental advantage of mass spectrometry over other detectors is the ability to simultaneously detect mycotoxins with different physicochemical properties in a single analysis, allowing multidetection. In addition, these detectors can provide structural information if needed. Conversely, matrix effects can make quantification difficult, the economic cost of the analysis is much higher than other techniques due to the high price of the equipment, and significant training is needed by analysts. These reasons hinder mass spectrometry’s implementation in control and production laboratories, resulting in a decrease in the number of samples analyzed by producers using this technique [57]. The FLD detector has also been used as an alternative to MS/MS detection. LC-FLD is less expensive and easier to use than LC-MS/MS, having good specificity and sensitivity. However, multidetection is more limited than in the case of mass spectrometer (MS) detection, and some mycotoxins require derivatization before quantification [53,70,71,74,75,76]. The development of new methodologies that facilitate the rapid, easy, and inexpensive analysis of mycotoxins in feed is essential for both research and feed control in small laboratories. These methods will contribute substantially to improving the control of feed production. Table S1 and Figure 2 show the different methodologies used for the determination of mycotoxins in feed in the articles reviewed (2019–2023).

### 3.2. Feed Analysis

**Figure 3:**
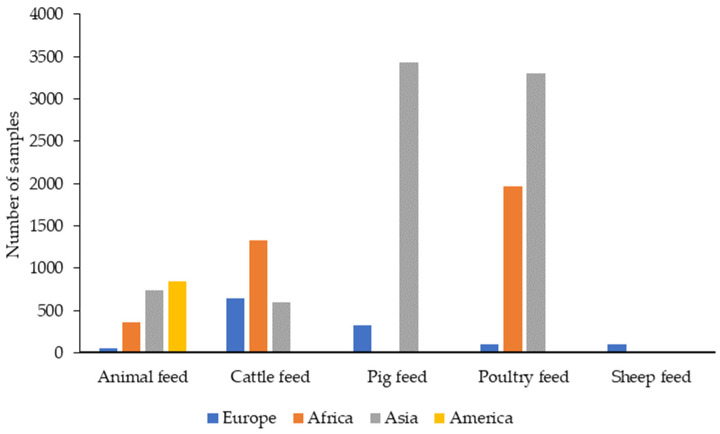
Number of feed samples analyzed by type and continent. Animal refers to samples for which the species was not indicated.

A total of 27 articles published between 2019 and 2023 were retrieved from PubMed with data on the presence and levels of different mycotoxins in feed for species intended for human consumption (Table S2). In total, the results of 13,818 feed samples were reported. According to species, 2557 samples corresponded to cattle feed, 3763 to pigs, 5363 to poultry, and 116 to sheep. The species was not indicated in 2003 samples, which were grouped as “animal feed”. Therefore, over the last five years, feed for poultry and feed for sheep have been the most and the least studied, respectively, for the presence of mycotoxins, as can be seen in Figure 3. 

The continent with the highest number of samples analyzed was Asia (8073 samples) followed by Africa (3670), Europe (1225), and the Americas (850, only from Brazil). Figure 3 shows the main types of feed and the number of samples analyzed in the different continents.

**Figure 4:**
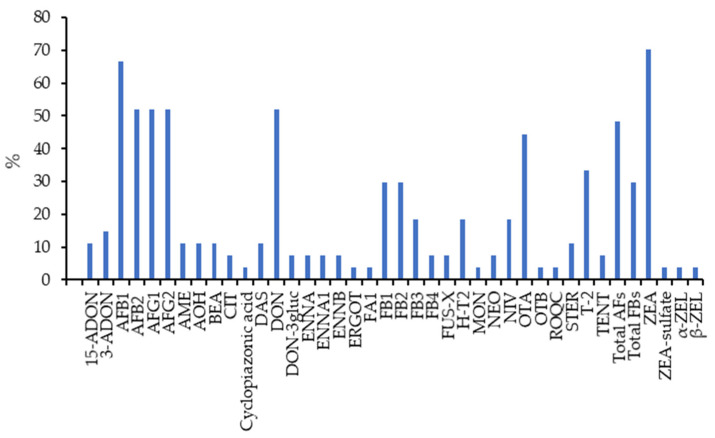
Mycotoxins analyzed in the recovered studies published between 2019 and 2023 and the percentage of articles that analyze each of them. 15-ADON: 15-acetyl deoxynivalenol; 3-ADON: 3-acetyl deoxynivalenol; AFB1: aflatoxin B1; AFB2: aflatoxin B2; AFG1: aflatoxin G1; AFG2: aflatoxin G2; AFs: aflatoxins; AME: alternariol monomethyl ether; AOH: alternariol; BEA: beauvericin; CIT: citrinin; DAS: diacetoxyscirpenol; DON: deoxynivalenol; DON-3gluc: DON-3 glucoside; ENNA: enniatin A; ENNA1: enniatin A1; ENNB: enniatin B; FA1: fumonisin A1; FB1: fumonisin B1; FB2: fumonisin B2; FB3: fumonisin B3; FB4: fumonisin B3; FBs: fumonisins; FUS-X: fusarenon-X; HT-2: HT-2 toxin; MON: moniliformin; NEO: neosolaniol; NIV: nivalenol; OTA: ochratoxin A; OTB: ochratoxin B; ROQC: roquefortine C; STER: sterigmatocystin; T-2: T-2 toxin; TENT: tentoxin; ZEA: zearalenone; α-ZEL: α-zearalenol; β-ZEL: β-zearalenol.

Of the 27 publications retrieved from the bibliographic search, between 30 and 70% of the articles provide data on the presence and levels of AFs, DON, ZEA, total FBs, total AFs, FB1, FB2, T-2, and OTA. AFB1 (in 67% of the retrieved articles) and ZEA (in 70%) are the most studied mycotoxins. This is expected because these mycotoxins are among those representing the greatest economic and animal health concern [20] and because different countries have established maximum limits for these compounds in feed. Other mycotoxins have also been analyzed but in a smaller number of articles (<20%). Figure 4 shows the mycotoxins studied and the percentage of selected publications in which they are present.

Data on the number of samples in each study, the prevalence, the highest values found, the countries, and the time interval for sample collection are given in Table S2. Some studies did not indicate the percentage of positive samples; in these cases, the prevalence range was obtained from the data retrieved. 

#### 3.2.1. Ochratoxin A

OTA is a common contaminant of cereals and is thermostable, which is why, if present in feed components, it is difficult to remove during feed production [89]. OTA is absorbed by animals through the gastrointestinal tract and exerts several toxic effects on them [89], although susceptibility varies between species. Ruminants, for instance, are less susceptible due to their metabolization in the rumen, whereas pigs are particularly vulnerable [90]. In 2023, the EFSA published a Scientific Opinion on the risks to animal health related to the presence of OTA in feed [90]. The panel concluded, based on the available data, that the risk of adverse effects related to OTA in feed is low.

**Figure 5:**
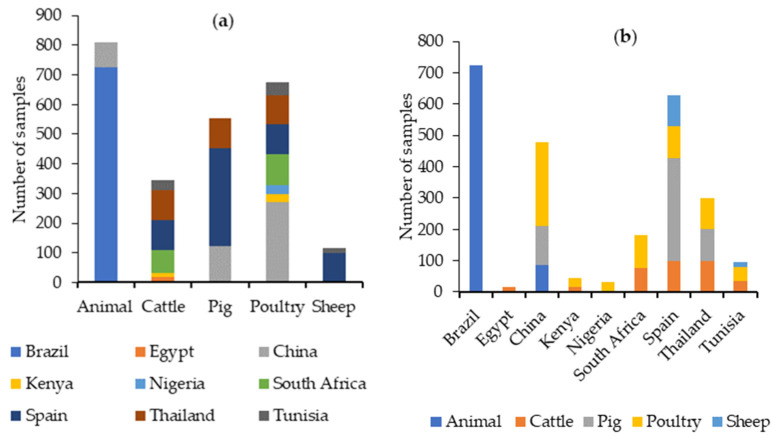
Number of feed samples analyzed for OTA by (**a**) feed type and (**b**) country. Animal refers to samples for which the species was not indicated.

The data from our literature search covering the period from 2019 to 2023 reveal that 2498 feed samples have been analyzed for OTA, with detectable levels of this toxin found in various types of feed worldwide (see Table S2 and Table 2, and Figure 5). The highest number of samples have been analyzed for OTA in samples grouped as “animal feed” and in poultry feed samples, followed by pig feed. Brazil, Spain, and China were the countries from which most feed samples were analyzed for OTA (Figure 5).

Globally, the percentage of positive samples (above the limit of quantification (LOQ)) for OTA (when indicated) has ranged from 0 to 56%. The highest percentage was observed in a Kenyan study on cattle feed (56%). The authors noted significant variability in both the prevalence and levels of OTA when analyzing samples collected during two different annual periods, and they concluded that, due to the maximum levels found, OTA is not of concern in feed in Kenya [61]. The maximum value of OTA found was 187.9 µg/kg in cattle feed in South Africa; however, in this case, the percentage of positive samples was low (3.9%) [81]. 

In the EU, the maximum levels for OTA in feed are set at 50 µg/kg for pigs and 100 µg/kg for poultry [91]. Most of the retrieved studies reported maximum OTA levels below these regulatory limits (see Table S2). However, a pig feed sample from Spain contained 65.5 µg/kg, exceeding the EU’s maximum limit [82]. Table 2 provides a summary of OTA contamination data found in the literature between 2019 and 2023.

Additionally, ochratoxin B (OTB) was found in 20% of 30 poultry feed samples from Nigeria, with a maximum level of 24 µg/kg [64]. OTB is a metabolite resulting from the dechlorination of OTA, and limited information is available on its effects on animal health [90].

#### 3.2.2. Zearalenone

ZEA is a common contaminant of corn, wheat, barley, sorghum, and rye, cereals normally present in feed. The main effects of ZEA and its metabolites, α-ZEL and β-ZEL, derive from their estrogenic characteristics and vary between species. Pigs are very sensitive, especially females, whereas cattle are more resistant. This variability depends on the metabolism of ZEA in different animals [92,93].

**Figure 6:**
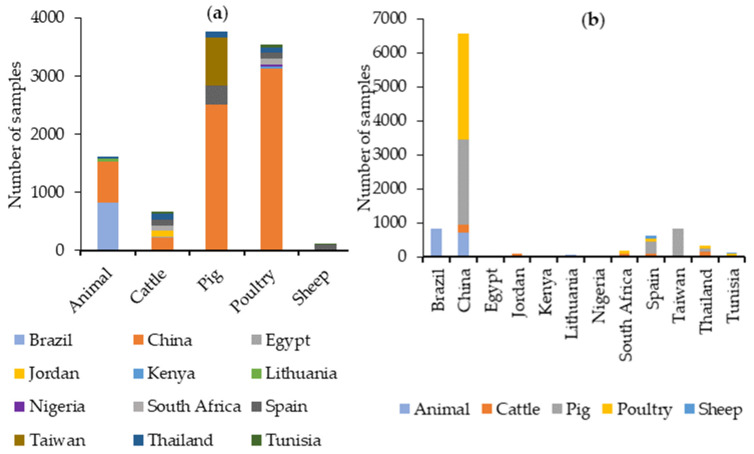
Number of feed samples analyzed for ZEA by (**a**) feed type and (**b**) country. Animal refers to samples for which the species was not indicated.

For ZEA, 9685 feed samples have been analyzed. Pig feed has been the most commonly analyzed feed type, followed by poultry feed. China is by far the country for which ZEA has been analyzed in the most feed samples, as can be seen in Figure 6. 

The data retrieved from our literature search between 2019 and 2023 indicate that ZEA has been found in all types of feed worldwide (see Table S2 and Table 3). Globally, the percentage of positive samples (>LOQ) (when indicated) has ranged from 3 to 100%. A prevalence of 100% for ZEA was found in studies conducted on cattle feed in Kenya [61] and poultry feed in China [77] and Kenya [61]. Very high levels of prevalence have also been found in samples grouped as “animal feed” (91%) [85], in cattle feed (99.3%) [77], and in pig feed in China (99–99.4%) [77,85]; in pig feed in Thailand (91%) [87]; and in poultry feed (94%) in China [85] and South Africa (99%) [67]. The maximum value found was 7681 µg/kg in pig feed in Spain; however, in this case, the percentage of positive samples was low (7%) [81]. 

For ZEA, the EU has set out a maximum level of 500 µg/kg for calves, dairy cattle, sheep (including lamb), and goats (including kids) and a maximum level of 250 µg/kg in pig feed [91]. Many authors report maximum results higher than those established by the European legislation. 

Some ZEA derivatives have been found in poultry feed in some studies: in Nigeria. ZEA sulfate was quantified in 13.3% of 30 samples, with a maximum level of 162 µg/kg [64], while α-ZEL and β-ZEL were observed in 99% of 105 samples in South Africa [67]. Table 3 shows a summary of the ZEA contamination data retrieved from the literature between 2019 and 2023. 

#### 3.2.3. Deoxynivalenol and Its Derivatives

DON mainly contaminates cereals. The EFSA considers that at dietary levels, no adverse effects are expected; however, if high levels are present in feed, there is a potential risk of chronic adverse effects in animals [94]. 

**Figure 7:**
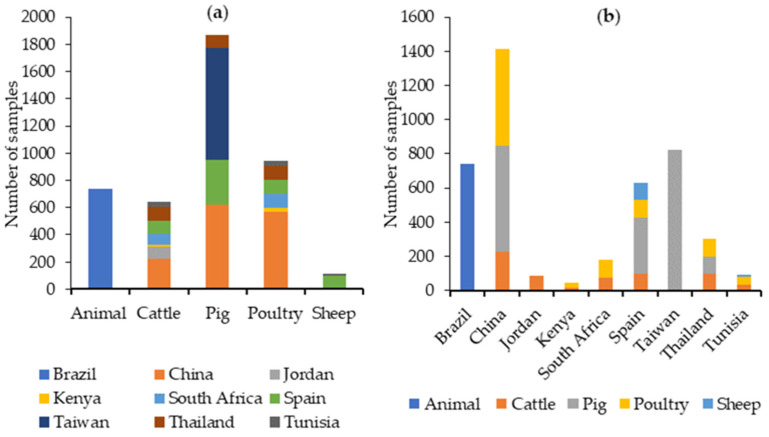
Number of feed samples analyzed for DON by (**a**) feed type and (**b**) country. Animal refers to samples for which the species was not indicated.

The data retrieved from our literature search between 2019 and 2023 indicate that DON was analyzed in 4445 samples. The most common type of feed analyzed was pig feed, followed by poultry feed. China is the country from which the most feed samples have been analyzed for DON, as can be seen in Figure 7.

DON has been found in all types of feed worldwide (see Table S2 and Table 4). Globally, the percentage of positive samples (>LOQ) (when indicated) has ranged from 4.4 to 100%. The highest percentages were found in studies conducted on cattle feed (99.3%) and pig feed (99.6%) in China [77], pig feed (91.4%) in Taiwan [69], and poultry feed in South Africa (98%) [67], Tunisia (100%) [86], China (99.7%) [77], and Kenya (100%) [61]. The maximum value found was in Taiwan (>5000 µg/kg) in pig feed; in this case, the percentage of positive samples was 91.4% [69].

Regarding DON, the EU has set out a maximum level of 5000 µg/kg, except for feed for pigs (900 µg/kg) and calves (<4 months), lambs, kids, and dogs (2000 µg/kg) [91]. In general, the levels observed in studies are lower than those set out in the EU legislation, except for pig feed samples from China (3712.2 µg/kg) [77] and Taiwan [69]. Table 4 shows a summary of the DON contamination data found in the literature between 2019 and 2023.

DON derivatives were also detected in the feed. 15-ADON has been analyzed in cattle, pig, and sheep feed, with a prevalence between 5% (poultry feed in Tunisia [86]) and 36% (cattle feed in Thailand [87]), and the highest level was 858.8 µg/kg in a South Africa cattle feed sample [67]. 3-ADON has also been found in these types of feed, with a prevalence (when indicated) between 1% in poultry feed in Thailand [87] and 95% in the same type of feed in South Africa [67]. In some cases, DON-3 glucoside (DON-3gluc) has also been found, such as in Kenya [61] (in high prevalence, 88–100%) and Thailand [60] (Table S2). Table 5 shows a summary of the DON derivatives’ contamination data found in the literature between 2019 and 2023. 

#### 3.2.4. Aflatoxins

AFB1, aflatoxin B2 (AFB2), aflatoxin G1 (AFG1), and aflatoxin G2 (AFG2) are produced by fungi of the genus Aspergillus in warm and humid climates. For this reason, climate change could have an impact on their presence in cereals and, therefore, in feed. This phenomenon was observed in Italy in 2003–2004 and in France in 2015, where dry and hot weather favored crop contamination with Aspergillus flavus, previously uncommon in these countries [16]. This is why monitoring the presence of AFs in the feed is continuously necessary. There is evidence for the carcinogenicity of AFB1 and AFG1, while for AFB2 and AFG2, such evidence is limited. AFB1 is the most frequent aflatoxin, and AFB2, AFG1, and AFG2 are usually only present when AFB1 is detected [95].

**Figure 8:**
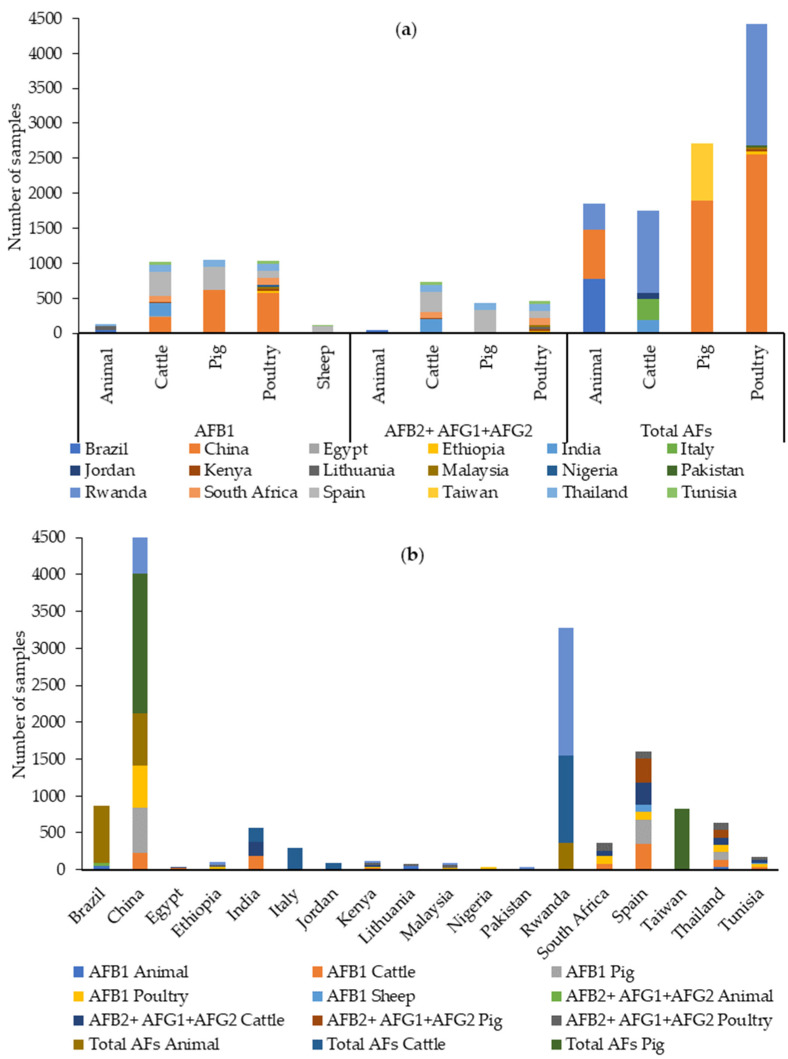
Number of feed samples analyzed for AFs by (**a**) feed type and (**b**) country. Animal refers to samples for which the species was not indicated.

The data retrieved from our literature search between 2019 and 2023 indicate that AFB1 was analyzed in 3342 samples, and AFB2, AFG1, and AFG2 were analyzed in 1781. AFB1 has been analyzed in approximately the same number of cattle, pig, and poultry feed samples, and most of these samples were analyzed in China, while AFB2, AFG1, and AFG2 were analyzed specifically in cattle feed in Spain, as can be seen in Figure 8.

Total AFs have been analyzed in 10,729 feed samples, especially in poultry feed and in China (Figure 8).

AFs have been found in all types of feed worldwide (see Table S2 and Table 6). Globally, for AFB1, the percentage of positive samples (>LOQ) (when indicated) has ranged from 3.1 to 100%. The highest percentages were found in studies on cattle (100%), pig (100%), and poultry (99.9%) feeds in China [77], on cattle (94%) and poultry (93%) feeds in Kenya [61], and on poultry feeds in South Africa (98%) [67] and Nigeria (83.3%) [64]. 

The highest values were found in poultry feed samples from Nigeria (760 µg/kg) [64], followed by poultry feed in Ethiopia (633.94 µg/kg) [78], samples grouped as “animal feed” in Brazil (390 µg/kg [66]), cattle feed in India (374.6 µg/kg) [74], poultry feed in Thailand (326.4 µg/kg) [87], and cattle feed in Kenya (134 µg/kg) [61] and in China (77.5 µg/kg) [77].

AFB2, AFG1, and AFG2 have been found in all types of feed analyzed. The prevalence and maximum values were as follows: AFB2: 1.3–100% and 188 µg/kg in poultry feed in Nigeria [64]; AFG1: 0.9–97% and 921.4 µg/kg in poultry feed in Ethiopia [78]; AFG2: 0–82% and 221.4 µg/kg in poultry feed in Ethiopia, respectively [78]. Table 6 shows a summary of the AF contamination data found in the literature between 2019 and 2023.

For AFB1, the EU has set out a maximum level of 10 µg/kg, except for feed for dairy cattle and calves, dairy sheep and lambs, piglets and young poultry animals (5 µg/kg) and feed for cattle, sheep, pigs, and poultry (20 µg/kg) [38]. Of the 18 authors reporting AFB1 values in feed intended for different food-producing animals, 8 of them [61,64,66,74,77,78,81,87] (44.4%) present maximum values higher than those set out by the European Commission, and in these studies, the prevalence is also high. As for the other studies, they report maximum values below or close to the limits established by the EU, with lower prevalence data.

When total AFs were analyzed, the percentage of positive samples (>LOQ) (when indicated) ranged from 1 to 97.5%. The highest percentages were found in studies on poultry feed in Ethiopia (94%) [78], Kenya (93%) [61], and Pakistan (97.5%) [59]. The highest values were found in cattle feed samples from India (406.1 µg/kg) [74], followed by pig feed in China (245 µg/kg [83]) and poultry feed in Ethiopia (1918.8 µg/kg) [78].

#### 3.2.5. Fumonisins

FBs are produced by species of fungi of the genus *Fusarium*. There are various different FBs, the most important being fumonisin B1 (FB1) (the most widespread), fumonisin B2 (FB2), fumonisin B3 (FB3), and fumonisin B4 (FB4). Species differences in the toxicokinetics of these compounds have been found [96]. Unlike other mycotoxins, FBs are polar molecules and, therefore, are soluble in water. In some cases, this property hinders their simultaneous extraction together with other less polar mycotoxins, since their extraction is less efficient when organic solvents are used [97].

The data retrieved from our literature search between 2019 and 2023 indicate that FB1 and FB2 were analyzed in 862 samples, especially from pig and poultry feeds, with Thailand being the country from which the most feed samples have been analyzed for FB1 + FB2. 

**Figure 9:**
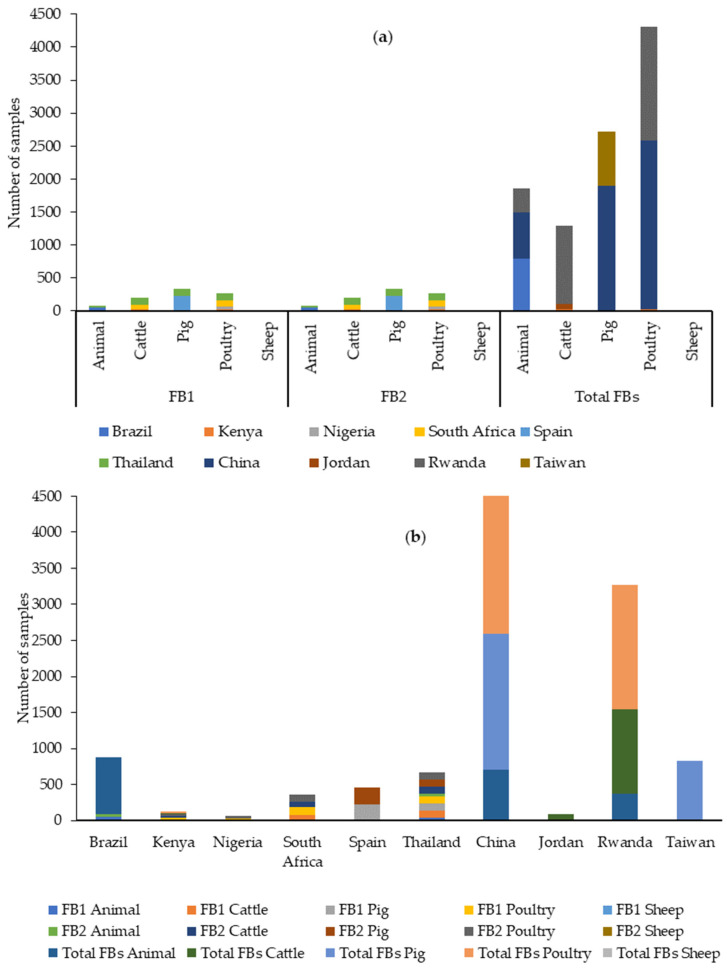
Number of feed samples analyzed for FBs by (**a**) feed type and (**b**) country. Animal refers to samples for which the species was not indicated.

Total FBs were evaluated in 10,173 feed samples, mostly from poultry feed, with China being the country from which the most feed samples have been analyzed (Figure 9).

Neither FB1 + FB2 nor total FBs were analyzed in sheep feed in any of the retrieved studies. They were found in other types of feed, usually with a high prevalence (see Table S2 and Table 7). For instance, FB1 + FB2 were found in close to 100% of samples in studies analyzing poultry feed from Kenya [61], Nigeria [64], South Africa [67], and Thailand [87]. Similarly, total FBs appeared in 100% of the samples of poultry and cattle feed in Kenya (4) and in a similar percentage of samples of animal (93.4%), poultry (99%), and pig (99%) feeds in China [83,84,85] (see Table S2 and Table 7).

The maximum levels found for FB1 and FB2 were 53,000 µg/kg and 2800 µg/kg, respectively, in samples grouped as “animal feed” from Brazil [66]. When total FBs were analyzed, the maximum level found was 17,490 µg/kg in Brazil in samples grouped as “animal feed” [57].

For FBs, the EU has set out a maximum level for FB1 + FB2 of 5000 µg/kg for pigs, 20,000 µg/kg for poultry, and 50,000 µg/kg for adult ruminants [91]. In general, the sum of even the maximum levels of both mycotoxins in each study is lower than the levels set out in the EU legislation, except for in samples grouped as “animal feed” from Brazil [66] and South Africa [67]. Table 7 shows a summary of the FB contamination data found in the literature between 2019 and 2023. 

FB3 and FB4 have also been detected in some feed samples. FB3 was analyzed in 289 samples of animal [60], cattle [61,81] and poultry feeds [61,64,67]. The prevalence was very high (close to 100%) in poultry feed and the maximum value found was 243 µg/kg in poultry feed in Kenya [61]. Regarding FB4, it has been studied in 73 samples of cattle [61] and poultry feeds [61,64], with a high prevalence (89–96.7%) in poultry feeds and a maximum level of 387.8 µg/kg in poultry feeds in Kenya [61].

#### 3.2.6. T-2 and HT-2

T-2 and HT-2 toxins are included in the group of trichothecenes and are produced by some species of the genus Fusarium. Both mycotoxins are found in cereals, especially in oats. Both are toxic to animals and humans, and different sensitivities have been observed among different species, with pigs being particularly sensitive. The EFSA, in its Scientific Opinion on the risks to animal and public health related to the presence of T-2 and HT-2 toxins in food and feed, considers that more studies on the presence of these toxins in feeds are needed [98]. 

**Figure 10:**
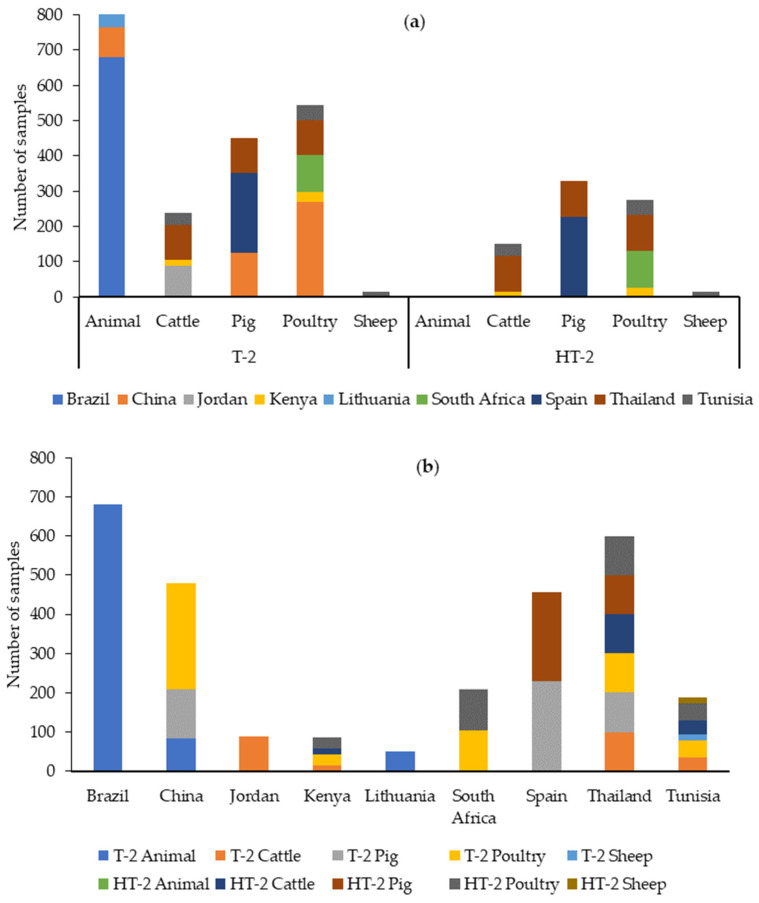
Number of feed samples analyzed for T-2 and HT-2 by (**a**) feed type and (**b**) country. Animal refers to samples for which the species was not indicated.

The data retrieved from our literature search between 2019 and 2023 indicate that T-2 was analyzed in 2069 samples. The highest number of samples was analyzed in samples grouped as “animal feed”, followed by poultry feeds. Brazil is the country for which most feed samples have been analyzed for T-2, as can be seen in Figure 10.

Globally, the percentage of positive samples (>LOQ) has ranged from 0.9 to 100% (see Table S2 and Table 8). The highest percentage (100%) was found in studies conducted on poultry feeds in South Africa [67]. Among the other studies, the maximum prevalence (when indicated) was 31.3% in Brazil [57]. The maximum value found was in Jordan (1734.6 µg/kg) in cattle feeds [62]. Table 8 shows a summary of the T-2 contamination data found in the literature between 2019 and 2023.

HT-2 was analyzed in 770 samples, especially from pig feeds. Thailand is the country from which most feed samples have been analyzed for HT-2 (Figure 10).

Globally, the percentage of positive samples (>LOQ) has ranged from 0.9 to 100% (see Table S2 and Table 8). The highest percentage (100%) was found in studies conducted on poultry feed in South Africa, although in this study, the maximum level was low (5.9 µg/kg) [67]. Among the other studies, the maximum prevalence (when indicated) was 37% in cattle feed from Tunisia [86], and in this country, the maximum value was also found (173.4 µg/kg). Table 8 shows a summary of HT-2 contamination data found in the literature between 2019 and 2023. 

Regarding T-2 and HT-2, the EU has set out a maximum level for the sum of both toxins of 250 µg/kg [98]. This is due to the rapid metabolization of T-2 to HT-2 and because T-2 toxicity may be partly due to the presence of this metabolite [91]. In those studies that analyzed both mycotoxins, the sum of the maximum levels of T-2 and HT-2 was below the established value, except for a study from Tunisia [86], in which the maximum value for T-2 alone was 956.5 µg/kg, and a study from Jordan, in which a sample of cattle feed contained 1734.6 µg/kg T-2 [62], well above the 250 µg/kg limit for the sum of both mycotoxins. 

#### 3.2.7. Other Mycotoxins

The trend in recent years, in addition to the study of the regulated mycotoxins, involves analyzing the presence of emerging mycotoxins, such as STER, NIV, BEA, enniatin A (ENNA), enniatin A1 (ENNA1), and roquefortine C (ROQC), among others. This has been made possible with the advances in LC-MS/MS, which has allowed the simultaneous detection of compounds with different physicochemical characteristics and with sufficient sensitivity. This technology is also enabling the discovery of hitherto unknown compounds that could be of great interest for both human and animal food safety in the future. In Table S2 and Table 9, data regarding NIV, neosolaniol (NEO), DAS, and STER are shown. In general, when indicated, these mycotoxins have been found at low prevalence values, except for NIV, which appeared in 94–96% of cattle and poultry feed samples from Kenya [61].

Other mycotoxins have also been analyzed, including AME, AOH, TENT, BEA, ENNA, ENNA1, enniatin B (ENNB), enniatin B1 (ENNB1), CIT, fusarenon-X (FUS-X), ERGOT, cyclopiazonic acid, MON, and ROQC (see Table S2). Typically, they have been found at a low prevalence, except for AME in poultry feed in South Africa (100%) [67]; BEA in pig feed in Spain (93.4%) [68], poultry feed in Tunisia (100%) [86] and Nigeria (100%) [64]; ENNB in pig feed in Spain (100%) [68] and in cattle (80%) and poultry (79%) feed samples [86] in Tunisia [86]; MON in poultry feed in Nigeria (93.3%) [64]; and ERGOT in poultry feed in Kenya (81%) [61].

#### 3.2.8. Multiexposure

Although individual mycotoxin levels often fall below the maximum regulated limits, they may still be present at low levels and probably in combination with others. The impact of this phenomenon on animal health remains uncertain [67,99]. Consequently, relying solely on the analysis of a single mycotoxin within a matrix does not provide adequate data for an adequate risk assessment [81].

Some authors have presented data on some mycotoxins in a sample by analyzing them individually, for instance using the ELISA methodology [57,62,65,69]; however, to investigate simultaneous exposure to multiple mycotoxins, technologies such as LC-MS/MS are required. This approach allows the simultaneous determination of several mycotoxins, despite their diverse physicochemical characteristics [61,67,85].

Among the 27 studies retrieved from the literature from 2019 to 2023, 12 provided data on mycotoxin co-occurrence. Their findings demonstrated co-occurrence in feed samples from all continents in which studies were carried out, with a high prevalence observed in most samples.

In the Americas, data were reported only from Brazil. The analysis of samples grouped as “animal feed” for the presence of AFs, DON, FBs, OTA, T-2, and ZEA revealed contamination in 87% of the samples, with between two and six toxins detected. The most frequent mixtures included DON–ZEA (45.2% of samples), Afs–DON (42.1%), and Afs–ZEA (41.5%) [57]. Another study conducted in Brazil, in which AFs, FBs, OTA, ZEA, and DON were analyzed in 45 samples grouped as “animal feed” samples, confirmed the coexistence of mycotoxins in 51% of the samples, detecting between two and five mycotoxins. The main mixture observed was FBs and DON, frequently coexisting with ZEA [66].

In Africa, several studies have documented the coexistence of mycotoxins. For example, in Kenya, 96% of samples (16 cattle and 27 poultry feed) were found to be contaminated with between two and eight toxins. All samples showed levels of AFs and ZEA, and 98% also contained FBs, 92% contained NIV, 89% contained DON, 87% contained DON-3gluc, 70% contained ERGOT, 6% contained T-2, and 4% contained HT-2. Furthermore, OTA coincided with FB1 in 25% of samples [60]. In South Africa, 50% of the 105 poultry feed samples analyzed exhibited detectable levels of the 17 mycotoxins under investigation. The primary combination, found in 51% of the samples, comprised AFs, FBs, ZEA (and derivatives), and DON (and derivatives). Additionally, FBs, ZEA (and derivatives), and DON (and derivatives) were present in 42% of the samples. Furthermore, both combinations, namely AFs, FBs, and ZEA (and derivatives), as well as AFs, FBs, HT-2, and T-2, appeared in 26% of the samples. The authors noted that the coexistence of DON, ZEA, and FBs is due to their production by *Fusarium* species [67]. Also, in South Africa, an analysis of 77 cattle feed samples for 23 mycotoxins revealed that 20% of them contained two mycotoxins in various combinations, while 66% were contaminated with at least three mycotoxins [81]. In Tunisia, the presence of 22 mycotoxins was investigated in 43 poultry, 35 cattle, and 16 sheep feed samples. This study found that two mycotoxins co-occurred in 97.6%, 94%, and 89% of poultry, sheep, and cattle feed samples, respectively. Additionally, 26% of the samples contained five different toxins, with eight mycotoxins coexisting in 5% of the samples [86]. In Nigeria, an analysis of 30 poultry feed samples revealed contamination with four mycotoxins in all samples, with AFs and FBs coexisting in 80% of them [64].

In Europe, a study conducted in Spain revealed that 69% of 228 samples of pig feed had the simultaneous presence of three to five mycotoxins, with a notable prevalence of combinations involving emerging mycotoxins, such as ENNs–BEA. Furthermore, 8.3% of the samples contained two or more regulated mycotoxins [68]. Similarly, another study in Spain demonstrated the coexistence of mycotoxins: out of 400 samples analyzed (including feed for cattle, pigs, poultry, and sheep, 100 samples each), 63.5% presented a coexistence of two to five mycotoxins. The most common combinations observed were ZEA and DON (23.8%), AFG2–ZEA–DON (13%), and AFB1–ZEA–DON (11%) [82]. 

In Taiwan (Asia), the analysis of pig feed samples revealed that 91.3% of the 823 samples analyzed exhibited co-contamination. Authors noted that two mycotoxins were present in 28.25% of the samples, three were present in 38.17%, and four were present in 23.54% of samples, with the most frequent combination being Afs–ZEA–FBs–DON, which were the four mycotoxins evaluated in the study [69]. In Thailand, the analysis of cattle feed samples showed that 96.6% contained two or more mycotoxins (ranging from 2 to 69). Combinations included ZEA co-occurring with FB1, DON, and AFB1, as well as FB1 appearing alongside AFB1 and DON. Additionally, mixtures of DON and AFB1, and AFB1 with ZEA, FB1, and DON were found [60].

In China, the co-occurrence of AFB1, DON, and ZEA in pig, poultry, and cattle feed samples was investigated. The authors highlighted that the simultaneous presence of these mycotoxins is very common in their study. The mixture of AFB1–DON–ZEA was found in 97.8% of pig feed samples, 98.4% of poultry feed samples, and 95.7% of cattle feed samples [77]. 

Hence, the occurrence of simultaneous contamination with multiple mycotoxins in feed is widespread worldwide and is present in an important percentage of samples. Moreover, the data indicate that as the number of mycotoxins analyzed increases, so does the detection of simultaneous contamination. These findings underscore the importance of considering combined toxicity studies of the most prevalent mixtures. In addition, such data should be taken into account for risk assessment studies and, if necessary, adjustments to legislation regarding the maximum mycotoxin levels in feed.

## 4. Internal Exposure

As was explained in the Introduction, feed analysis alone does not adequately assess the exposure of animals to mycotoxins. Another approach, internal exposure (or ABM), based on the analysis of biomarkers of exposure in biological fluids or tissues, complements the data obtained using the external exposure approach and helps in risk assessment. For ABM, plasma, feces, and urine are the most commonly used matrices [100]. Plasma samples can reveal systemic exposure to toxins, while feces and urine samples can provide information on toxin excretion and possible elimination pathways. Biomarker levels in the urine are expected to be higher than those in the plasma or blood; however, this matrix has some disadvantages, such as greater inter- and intra-individual volume variation, and urine collection is often more difficult for animals compared to blood or plasma [101]. 

When explaining the levels of mycotoxins in animal biological matrices, it should be taken into account that the non-detection of metabolites can sometimes be explained by their low levels in biological matrices and/or the high detection limit of the applied method. Furthermore, it is challenging to determine the appropriate sampling time, as the levels in the analyzed biological matrix depend on the time elapsed since the last ingestion of contaminated feed and on the kinetics of the compounds [102], or the optimal site for sample extraction, because levels can vary among them [103]. Also, identifying good biomarkers is crucial because differences in metabolism have been observed for OTA [104], FBs [105], AFs [106], and CIT [107] due to the age of the animal. Age-related toxicokinetic differences were observed in pigs, leading to greater internal exposure to toxins in juveniles due to immaturity in metabolic activity or organ development and a higher feed intake/weight ratio. After the intravenous administration of ZEA into growing piglets, it was converted to α-ZEL at a lower rate than in old pigs; moreover, after the intravenous administration of ZEA-14G, the conversion to ZEA has been observed to a similar extent as in older pigs, whereas its oral bioavailability is greater for juveniles [108]. In addition, equations are needed to relate intake and levels in the analyzed biological matrix. Dänicke and Winkler (2015) developed equations to estimate exposure to ZEA by analyzing ZEA and its derivatives in some biological matrices of pigs and cows, but they proved difficult to apply to individual animals due to the variability found [50]. 

**Figure 11:**
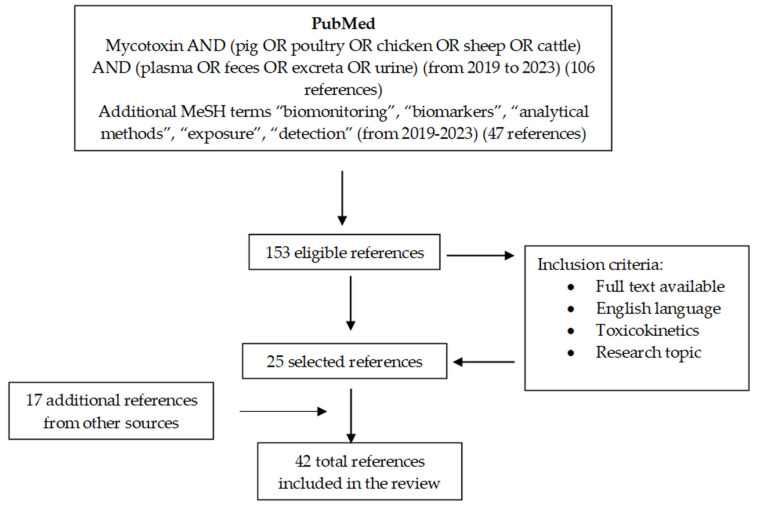
Flow diagram of excluded and included studies based on PRISMA Statement.

Based on the PRISMA statement [52], a systematic review regarding the biomonitoring of mycotoxins in animal biological fluids from 2019 to 2023 (extending from 2017, when no data were found) was performed, and the results are summarized in Table 10 and Table S3 and Figure 11.

After performing the review, it was observed that almost all of the studies analyzed mycotoxins in biological fluids to study their metabolism in different animal species to obtain the best biomarkers for each mycotoxin, species, and matrix. For this purpose, animals were fed using mycotoxin-contaminated feeds. A smaller number of the reviewed studies analyzed mycotoxin levels in biological fluids to determine the efficacy of mycotoxin binders in removing mycotoxins from the contaminated diet [49,100,109,110,111]. Only two studies were found to have the aim of biomonitoring mycotoxins in animals [103,112].

**Table 10:** Summary of studies on the levels of mycotoxins and their derivatives in animal biological samples.

Mycotoxins and Metabolites	Matrix	Animal	Year	Reference
OTA, OTα	Plasma Kidney, liver, muscle	Pig	2023	[109]
AFB1, AFB2, AFG1, AFG2, AFM1, OTA, OTB, ZEA, DON, 3- and 15-ADON, DOM-1, T-2, HT-2; STER, NEO, DAS, FUS-X, NIV (and their glucuronide or sulfate conjugates)	Plasma	Poultry Pig Sheep Cattle	2023	[112]
AFB1, AFB2, AFG1, AFG2, AFM1 and AFM2	Plasma	Poultry Cattle	2023	[49]
DON, isoDON, DON-3GlcA, DON-15GlcA, DOM-1, DOM-3GlcA, DOM-15GlcA	Serum Urine	Pig	2023	[113]
OTA, OTα	Plasma Feces/excreta, urine liver, kidney, muscle, skin, fat	Poultry Pig	2022	[114]
FB1, FB2, FB3 HFB1, HFB2, HFB3	Excreta	Poultry	2022	[115]
DON, 3 and 15-ADON, DOM-1, ZEA, α-ZEL, β-ZEL, α-ZAL, β-ZAL, ZAN, OTA, AFB1, AFB2, AFG1, AFM1, T-2, HT-2, OTα	Feces Serum	Pig	2021	[39]
DON, 3-ADON, 15-ADON, DOM-1, ZEA, α-ZEL, β-ZEL, α-ZAL, β-ZAL, ZAN, OTA, AFB1, AFB2, AFG1, AFM1, T-2, HT-2, NIV, TEN, AOH, AME, ATX-I, CIT, DAS, FUS-X, STER, T-2 triol, OTα, HFB1, DH-CIT, ENNs and BEA	Urine	Pig	2021	[116]
DON, DOM-1, ZEA, α-ZEL, OTA, OTα, CIT, DH-CIT	Plasma Urine	Pig	2021	[117]
CIT, DH-CIT	Plasma	Poultry Pig	2020	[107,118]
FM1, HFB1	Plasma	Poultry	2020	[110]
DON, DOM-1, 3/15ADON, AFB1, AFM1, ENNA, ENNA1, ENNB, ENNB1, BEA, FB1, FB2, OTA, ZEA, α-ZEL, β-ZEL, α-ZAL, β-ZAL, ZAN, TEA, AOH, AME, T-2	Plasma Urine Feces/excreta	Poultry Pig	2019	[44]
AFB1, DON, DON-s, DON-GlcA, ZEA, ZEA-GlcA	Plasma Feces/excreta Urine	Poultry Pig	2019	[100]
DON, DOM1, 3/15ADON, AFB1, AFM1, ENNA, ENNA1, ENNB, ENNB1, BEA, FB1, FB2, OTA, ZEA, α-ZEL, β-ZEL, α-ZAL, β-ZAL, ZAN, TEA, AOH, AME, T-2	Plasma DBS	Poultry Pig	2019	[119]
ZEA, α-ZEL, βZEL, ZAN, α-ZAL, β-ZAL, ZEN14G, ZEN14S, ZEA-14GlcA, α-ZEL-14GlcA, α-ZEL-7GlcA, β-ZEL-14GlcA, and β-ZEL-16GlcA	Plasma	Pig	2019	[48]
AFM1, DON, DOM-1, ZEA, α-ZEL, β-ZEL, FB1, OTA, DOM1,	Urine	Pig	2019	[120]
AFB1, AF2, AFG1, AFG2, AFM1, AFP1, AFQ1, AFB1-N^7^-guanine	Feces/excreta Ileal content	Poultry	2019	[121]
FB1, FB2, FB3, pHFB1, HFB1, pHFB2, HFB2, FB3, pHFB3, HFB3	Feces Urine Serum	Pig	2018	[111]
FB1, pHFB1, HFB1 and FB2	Plasma	Poultry	2018	[122]
ZEA, ZAN, β-ZAL, α-ZAL, β-ZEL, α-ZEL	Heart, liver, spleen and muscle	Pig	2018	[123]
FUS-X, NIV	Plasma Feces Urine	Goats	2018	[124]
AFB1	Liver and gizzards	Poultry	2017	[125]
ZEA, α-ZEL, β-ZEL, Phase II metabolites	Urine Feces	Poultry	2017	[126]

AFB1: aflatoxin B1; AFB2: aflatoxin B2; AFG1: aflatoxin G1; AFG2: aflatoxin G2; AFM1: aflatoxin M1; AFP1: aflatoxin P1; AME: alternariol monomethyl ether; AOH: alternariol; ATX-I: altertoxine I; CIT: citrinin; BEA: beauvericin; DAS: diacetoxyscirpenol; DBS: dried blood spot; DOM-1: deepoxydeoxynivalenol; DON: deoxynivalenol; 3-ADON: 3-acetyldeoxynivalenol; 15-ADON: 15-acetyldeoxynivalenol; DON-3GlcA: DON-3-glucuronide; DON-15GlcA: DON-15-glucuronide; DON-s: DON sulfate; ENNA: enniatin A; ENNA1: enniatin A1; ENNB: enniatin B; ENNB1: enniatin B; FBs: fumonisins; FUS-X: fusarenon-X; DH-CIT: dihydrocitrinone; HFBx: hydrolized FBx; HT-2: HT-2 toxin; NEO: neosolaniol; NIV: nivalenol; OTα: ochratoxin α; OTA: ochratoxin A; pHFBx: partially hydrolized FBx; STER: sterigmatocystin; T-2: T-2 toxin; TEN: tentoxine; ZAL: zearalanol; α-ZAL: α-zearalanol; β-zearalanol; ZAN: zearalanone; ZEA: zearalenone; α-ZEL: α-zearalenol; β-ZEL: β-zearalenol; ZEL-14GlcA: ZEA-14-glucuronide; ZEL-16GlcA: ZEA-16-glucuronide; ZAN: zearalanone; ZAN-14GlcA: ZAN-14-glucuronide.

### 4.1. Analytical Methods

**Figure 12:**
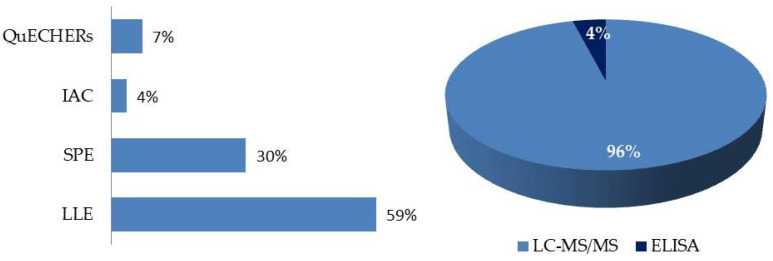
Extraction and detection techniques for mycotoxin determination in animal plasma/feces/urine/excreta according to the articles reviewed (2019–2023).

In the retrieved studies published in the last five years, we observed various analytical techniques to detect and quantify toxins in biological samples, as shown in Figure 12 and Table S3. The development of these methods could be more challenging than those for the determination of mycotoxins in feed, due to the complexity of the matrices analyzed and the need for better sensitivities [50]. 

In general, mycotoxins are found in biological samples at extremely low levels (typically at ng/mL levels). Hence, the analysis of mycotoxins in biological samples, such as plasma, blood, urine, feces, excreta, or tissue samples, requires the development and validation of sufficiently sensitive analytical methods [39,49]. Moreover, being able to detect a number of these compounds simultaneously is essential to reduce analysis time and cost. For these reasons, LC-MS/MS is the analytical technique mainly employed for ABM studies [43]. However, when MS detectors are utilized, matrix components may interfere with analyte retention, reduce method purification, recovery, and sensitivity, and produce matrix effects. To overcome these challenges, different steps have been included to extract analytes and clean matrix components. 

The most commonly used extraction, clean-up, and enrichment procedures were liquid-liquid extraction (LLE) using various organic solvents (64% of the articles reviewed). Ethyl acetate (EtOAc) [44,100,109,114,116,126], ACN [39,44,48,49,107,116,118,119], MeOH [111,113,125], and their mixtures, such as MeOH/ACN [111], MeOH/EtOAc [44] and ACN/H_2_O [111,115,121,124], were the most commonly used solvents for mycotoxin LLE. Acid solutions were also employed to alter protein binding, thereby enhancing extraction efficiency [44,49,109,111,113,115,116,121]. SPE, based on the retention of analytes on a fixed support in a cartridge, which is useful for purifying and preconcentrating analytes, was used in 24% of the reviewed articles [44,107,110,115,121,122,124,127]. The procedure developed by Arce et al. (2020) [128] employing Captiva EMR-lipid cartridges to mitigate matrix effects by removing phospholipids from the plasma during sample preparation was used by Muñoz et al. (2023) [112], while the QuEChERS method was used by De Baere et al. (2023) [49] and Yan et al. (2018) [123]. The use of IAC was limited due to its inhibition of the simultaneous extraction of multiple compounds, making it more suitable for single mycotoxins, but not in multidetection, only being used by Gambacorta et al. (2019) [120]. Furthermore, some authors have utilized an enzymatic deconjugation method to evaluate phase II metabolites. Plasma, feces, or urine samples were incubated with β-glucuronidase or β-glucuronidase/arylsulfatase before the selected extraction procedure was applied [112,113,116].

Regarding the detection and quantification of mycotoxins and their metabolites in plasma, feces, urine, or excreta samples, LC has become the main tool and was used in nearly all of the referenced articles. Ultra-LC, which enhances chromatographic resolution and efficiency while reducing solvent consumption and run times, was reported in 23.1% of the methods [49,107,110,115,122]. ELISA [125] has been scarcely used (Figure 12). In the reviewed articles, either a single mycotoxin (or structurally related compounds) or multiple mycotoxins simultaneously were determined (60% and 40% of the reviewed studies, respectively).

The chromatographic system coupled with tandem MS has proven to be a valuable and confirmatory technique for the determination of mycotoxins and metabolites in biological fluids, appearing in 92% of all referenced articles. The main ionization source used was electrospray (ESI). As for the mass analyzers, triple quadrupoles (QqQs) or quadrupole ion traps (QTraps) were used (76% and 24%, respectively). Quadrupole time of flight (QTOF) [126] and Q-Orbitrap [48] have also been employed.

### 4.2. Biomarkers of Exposure

Some compounds (Table 10) have been identified in the investigations conducted to evaluate toxicokinetics and identify biomarkers of exposure following oral and/or intravenous mycotoxin administration. These studies were mainly conducted on pigs, poultry, and cattle. 

For AFs, the available data demonstrate significant heterogeneity, possibly due to the diverse metabolic pathways among species (e.g., in ruminants due to the rumen or due to age differences) [106]. AFB1 undergoes a wide range of biotransformation reactions in various animal species. Phase I reactions (O-dealkylation, ketoreduction, hydroxylation, and epoxidation) result in the formation of metabolites, such as aflatoxin P1 (AP1), aflatoxicol (AFL), AFM1, aflatoxin Q1 (AFQ1), and aflatoxin B2a. Aflatoxin-8,9-epoxide, the product of epoxidation, is highly reactive and binds to DNA (forming guanine adducts) or proteins. Furthermore, phase II conjugation with glutathione plays a crucial role in detoxification. Among these, AFB1, AFM1, and AFQ1 consistently emerge as possible biomarkers in various animal species [49,129]. 

In the reviewed studies, free OTA was found in all biological fluids studied, being more prevalent than its metabolites, which supports the low level of OTA biotransformation reported by some authors [129]. Regarding its metabolites, OTα was detected in feces and urine [109,114], and only small amounts were detected for OTB, the hydroxylated form of OTA, or phase II metabolites. Therefore, the parent compound was proposed as an OTA biomarker of exposure in both excreta and plasma samples [100,109,130]. 

DON undergoes several biotransformation pathways depending on the species. Deepoxidesoxynivalenol (DOM-1) is significantly formed in ruminants and pigs. As for phase II reactions, glucuronidation predominates in pigs and ruminants, whereas the formation of sulfates in poultry is dominant [100]. 

The presence of ZEA and its metabolites in meat, eggs, and milk is generally very low, although they can be detected in the blood, liver, intestine, urine, or feces [131]. The main metabolites of ZEA in animal species are α-zearalenol (α-ZEL) and β-zearalenol (β-ZEL), while their different phase II glucuronide or sulfate conjugates are also detected in different species, such as ZEA-14GlcA, ZEA-16GlcA, and ZEA-14,16diGlcA [4]. The formation of α-ZEL or β-ZEL depends on the species [48,50,100], and this is significant because β-ZEL is less estrogenic than α-ZEL [93] and the diverse metabolism of ZEA correlates with the varying sensitivity of animals. For example, α-ZEL is predominantly produced in pigs and turkeys, while in cattle, goats, broilers, and poultry, β-ZEL is more abundant [93], making these species more resistant to the estrogenic effect of ZEA. In fact, in ruminants, a high level of feed intake has been associated with higher systemic exposure to ZEA, probably due to the shorter time that these compounds are in contact with rumen microbes, resulting in lower metabolization [50]. The reconversion of these metabolites to ZEA has also been proposed [53]. 

In the case of FBs, they have demonstrated high stability in several species; however, they can be partially (pHFBs) or fully (HFBs) hydrolyzed by intestinal microbiota, depending on the species, and these metabolites have been detected in excreta. No phase II metabolism (sulfation or glucuronidation) has been observed [110]. FBs exhibit low absorption and are predominantly excreted via the fecal route. Both FBs and their hydrolyzed forms could be considered as appropriate animal biomarkers of exposure [115].

Very few studies were retrieved from 2019 to 2023 which studied other mycotoxins. For CIT, biotransformation into its metabolite DH-CIT has been observed, although to a limited extent, and differences are found between species for all of its toxicokinetic parameters, including oral bioavailability [107,132].

### 4.3. Biomarkers of Exposure According to the Animal Species

#### 4.3.1. Cattle

No studies have been found for sheep. For cattle, only DeBaere et al. (2023) analyzed plasma samples to test the effects of an AFB1-specific mycotoxin detoxifying agent (bentonite) using contaminated feed. Their method was able to detect AFs and the metabolites AFM1 and AFM2. Only AFB1 and AFM1 were detected [49]. 

#### 4.3.2. Pigs

Tkaczyk et al. (2021) identified AFB1, AFM1, and AFB2 as biomarkers of AFB1 exposure in the urine of pigs [39]. 

After feeding pigs using an OTA-contaminated diet, this mycotoxin was quantified in biological samples [114,116,122]. Low levels of OTα (lower than that of OTA) were also detected, and it may be considered as a biomarker of OTA in pigs [114,117].

The main route of excretion of DON in pigs is the urine [120,133], and in this matrix, DON is the most abundant biomarker, although very low concentrations of metabolites, such as DON-GlcA, are also detected. A positive correlation has been demonstrated between DON intake and the level of this toxin and its metabolites in the urine or blood. According to Tkaczyk et al. (2021) [39], low levels of DON and DOM-1 and high levels of DON’s conjugated forms, DON-15-glucuronide (DON-15GlcA) and DON-3-glucuronide (DON-3GlcA), are found in the serum and plasma [44,113], and thus they have been proposed as good biomarkers of exposure to DON in these matrices. Biomarkers for DON were not identified in feces [44]; although they appeared in some studies, their concentrations were below the detection limits of the method used [39,100]. 

In some studies, after the oral administration of ZEA, both the parent compound and its glucuronide conjugates, but no other metabolites, could be detected in pig plasma and urine. Furthermore, these glucuronides are observed at higher levels than ZEA, which is why they have been proposed as good biomarkers of exposure [44,120]. In feces, unlike β-ZEL, ZEA and α-ZEL were found [44]. 

FBs have a low bioavailability and are very stable once absorbed by pigs. They are mainly eliminated through the feces. FB1 was detected in urine and feces, although low levels of pHFB1 (partially hydrolyzed FB1) and HFB1 (hydrolyzed FB1) have been found due to the hydrolyzation of FB1 by the fecal microbiota. HFB1 levels have also been detected in the serum [111]. 

Following the dietary administration of CIT to pigs, its absorption was complete and this toxin was rapidly eliminated. Then, CIT and DH-CIT were detected in pig plasma samples [107]. 

#### 4.3.3. Poultry

AFB1 and AFL were detected in poultry plasma when administered as a single oral bolus [44,49,100]. AFB1 [100], AFM1, and AFB1-N7-guanine were detected in urine and feces samples [121]. AFM1 was detected in both the excreta and ileal content. The higher concentrations found in excreta [121] suggest primary excretion via urine. However, it cannot be used as a urinary aflatoxin biomarker because it was detected in both matrices. Meanwhile, AFB1-N7-guanine can be used as a urinary biomarker since it was found in poultry excreta but not in the ileal content. 

OTA and OTα were the main biomarkers identified in the plasma and excreta of broilers after OTA administration [44,100,114]. Other metabolites were searched for in these matrices but were not present (or only in trace amounts). This confirms the limited biotransformation of OTA.

In broilers and after a single oral dose of DON, due to its low biotransformation and excretion, only DON sulfate (DON-s) was found in the plasma and excreta [44]. Consequently, in these matrices, DON, DOM-1, and phase II metabolites are not considered as ideal DON biomarkers. 

In poultry, ZEA is characterized by its low oral bioavailability and rapid elimination. ZEA biomarkers may include metabolites such as α-ZEL, β-ZEL, and zearalenone glucuronides (ZEN-16GlcA, ZEN-14GlcA, and ZEN-14,16diGlcA) found in the plasma, urine, and feces [126]. 

Research on FBs and their metabolites in poultry excreta is very limited. After the oral administration of feed contaminated with FB1, low levels of FB1 and traces of pHFB1 were detected in the plasma, and no phase II metabolites or HFB1 were found [110,122].

As in pigs, after administration in the diet of poultry, CIT was well absorbed and rapidly eliminated. Only CIT and DH-CIT were detected in poultry plasma samples [107]. 

In Table 11, the main biomarkers found in poultry and pigs are shown.

### 4.4. Analysis of Animal Samples

In the reviewed articles, there are very few studies in which the determination of mycotoxins and their metabolites for ABM was the main objective. Baranski et al. in 2021 [103] studied the presence of ZEA and its metabolites α-ZEL and β-ZEL in blood samples from sick dairy cows. ZEA was detected in all samples; however, no metabolites were found. The authors observed different concentrations of ZEA depending on the collection site, with the caudal median vein of the liver having the highest concentrations of ZEA. On the other hand, Muñoz et al. (2023) analyzed 19 compounds (mycotoxins and some derivatives, including AFB1, AFB2, AFG1, AFG2, AFM1, OTA, OTB, STER, T-2 y HT-2, ZEA, DON, DOM-1, 3-ADON, 15-ADON, NIV, FUS-X, NEO, and DAS) in a large number of animal plasma samples from poultry, pigs, cattle, and sheep to evaluate mycotoxin exposure in these animals [112]. Except for one poultry sample that showed the presence of DON, no mycotoxin or derivatives were detected. After enzymatic treatment with β-glucuronidase/arylsulfatase, all samples displayed the presence of STER, with no differences found between the species. Remarkably, this mycotoxin appeared in 100% of the analyzed samples, despite its low prevalence and levels in the feed. This suggests that animals may have been exposed through routes other than feed, emphasizing the role of the farm environment [112].

A limited number of biomonitoring studies have been conducted to assess the efficacy of mycotoxin binders in reducing mycotoxin toxicity in animal feed [49,100,109,110,111,134]. To evaluate the efficacy and safety of these detoxifying agents, mycotoxin concentrations were measured in chicken and pig samples obtained during a feeding trial. Depending on the chemical structures of the mycotoxins, binders may exhibit varying degrees of effectiveness against specific mycotoxins. Various types of mycotoxin binders are discussed in the reviewed articles, each with distinct mechanisms of action and applications, i.e., bentonite serves as an AFB1 and DON detoxifier in chickens [49,100], OTA-hydrolyzing enzyme is utilized in pigs [109], and plasma-activated water [134] and fumonisin esterase act as FBs detoxifiers in pigs and chickens [110,111].

## 5. Conclusions

Given the persistent and widespread occurrence of mycotoxins, it is crucial to implement control and continuous monitoring measures to ensure food safety, mitigate risks to animal and human health, and diminish the economic impact on the livestock industry and the global economy. This involves evaluating both external exposure through feed analysis and internal exposure through the analysis of biomarkers in animal biological matrices, as they are complementary. This manuscript provides a state-of-the-art review of mycotoxin exposure monitoring in food-producing animals over the past five years. It encompasses both external and internal exposure (animal biomonitoring) assessment methods. Additionally, it identifies challenges associated with these approaches.

In the literature over the last five years, various studies have analyzed nearly 14,000 feed samples intended for cattle, pigs, poultry, and sheep for the presence of mycotoxins. Feed for poultry was the most commonly studied, while feed for sheep was the least commonly studied. Asia emerged as the region with the highest number of samples analyzed. The most studied mycotoxins included AFs and ZEA, owing to their significance with regard to animal health and economic cost and the necessity of complying with regulations. Despite mycotoxins being widespread, they were generally found at levels lower than those set out in EU regulations. However, some samples exhibited higher values, emphasizing the necessity of continuous monitoring. Furthermore, additional mycotoxins, such as emerging ones, should be incorporated into monitoring studies. Multiexposure has been demonstrated in studies worldwide, detecting that as the number of mycotoxins analyzed increases, so does the detection of simultaneous contamination. Therefore, further research is necessary to assess the combined toxicity of mycotoxins and their implications for animal health. 

Concerning the internal exposure approach (ABM), it is an essential part for evaluating mycotoxin exposure. Some mycotoxins and metabolites have been identified as potential exposure biomarkers in the plasma, urine, and feces of animal species, especially pig and poultry. However, further research is needed to better understand the metabolism of mycotoxins, because toxicokinetic studies have only been carried out for a few mycotoxins, particularly those regulated in feed and foods, and emerging mycotoxins should also be studied. Despite their significance as food producers, no toxicokinetic studies have been found for sheep, and very few exist for cattle. Moreover, other aspects should be considered for ABM studies: new methodologies should be developed which are capable of simultaneously detecting multiple compounds with sufficient sensitivity for detecting the low levels encountered in biological matrices. To achieve this, reference materials and analytical validation guidelines are required. Furthermore, the relationship between biomarker levels and intake or their effects on animal health must be established. 

To summarize, by combining both approaches, namely internal and external exposure (ABM), the early detection and management of mycotoxin exposure in animals can be enhanced, thereby benefiting animal and human health as well as the livestock sector.

## Figures and Tables

**Table 2:** Summary of OTA contamination data found in the literature (2019–2023).

Matrix	n	Prevalence (%)	Maximum Level (µg/kg)	Collection	Countries
Animal * feed	810	2–34.3	87.8	2016–2021	Brazil [57,66], China [84]
Cattle feed	345	3.9–56	187.9	2014–2020	Tunisia [86], Egypt [70], Thailand [87], South Africa [81], Spain [82], Kenya [61]
Pig feed	552	0–7	65.5	2017–2020	China [84], Spain [68,82], Thailand [87];
Poultry feed	675	0–26.7	27	2013–2020	China [84], South Africa [67], Tunisia [86], Nigeria [64], Thailand [87], Spain [82] and Kenya [61]
Sheep feed	116	8–31	45.3	2016–2020	Tunisia [86], Spain [82]
TOTAL	2498	0–56	187.9	2013–2021	

* Animal refers to samples for which the species was not indicated.

**Table 3:** Summary of the ZEA contamination data found in the literature (2019–2023).

Matrix	n	Prevalence (%)	Maximum Level (µg/kg)	Collection	Countries
Animal * feed	1612	29–91	2503.9	2016–2021	Brazil [57,66], China [83,84,85], Lithuania [73], Thailand [60]
Cattle feed	658	3–100	1793.7	2014–2020	China [77], Egypt [70], Jordan [62], Kenya [61], South Africa [81], Spain [82], Thailand [87], Tunisia [86]
Pig feed	3762	7.0–99.4	7681	2015–2021	China [77,83,84,85], Spain [82], Thailand [87], Taiwan [69]
Poultry feed	3537	56–100	1490	2013–2021	China [77,83,84,85], Kenya [61], Nigeria [64], South Africa [67], Spain [82], Thailand [87], Tunisia [86]
Sheep feed	116	3–52	658	2016–2020	Tunisia [86], Spain [82]
TOTAL	9685	3–100	7681	2013–2021	

* Animal refers to samples for which the species was not indicated.

**Table 4:** Summary of the DON contamination data found in the literature (2019–2023).

Matrix	n	Prevalence (%)	Maximum Level (µg/kg)	Collection	Countries
Animal * feed	871	26.5–87.9	4969.1	2016–2021	Brazil [57,66], Lithuania [73], Thailand [60]
Cattle feed	641	37–99.3	2490	2016–2020	China [77], Jordan [62], Kenya [61], South Africa [81], Spain [82], Thailand [87], Tunisia [86]
Pig feed	1871	4.4–99.6	>5000	2015–2020	China [77], Spain [68,82], Taiwan [69], Thailand [87]
Poultry feed	946	31–100	2970.1	2015–2020	China [77], Kenya [61], South Africa [67], Spain [82], Thailand [87] Tunisia [86]
Sheep feed	116	6.0–72	887	2016–2020	Tunisia [86], Spain [82]
TOTAL	4445	4.4–100	>5000	2015–2021	

* Animal refers to samples for which the species was not indicated.

**Table 5:** Summary of the DON derivatives contamination data found in the literature (2019–2023).

Matrix	n	Prevalence (%)	Maximum Level (µg/kg)	Collection	Countries
15-ADON					
Animal * feed	0				
Cattle feed	212	17–36	858.8	2016–2019	South Africa [81], Thailand [87], Tunisia [86]
Pig feed	100	16	83.2	n.i. **	Thailand [87]
Poultry feed	248	5–35	840.7	2015–2017	South Africa [67], Thailand [87], Tunisia [86]
Sheep feed	16	25	19	2016–2017	Tunisia [86]
TOTAL	576	5–36	858.8	2015–2019	
3-ADON					
Animal Feed	0				
Cattle feed	212	3–16.9	300.0	2016–2019	South Africa [81], Thailand [87], Tunisia [86]
Pig feed	100	n.i.	n.i.	n.i.	Thailand [87]
Poultry feed	248	1–95	167.9	2015–2017	South Africa [67], Thailand [87], Tunisia [86]
Sheep feed	16	n.i.	n.i.	n.i.	Tunisia [86]
TOTAL	576	1–95	300	2015–2019	
DON-3gluc					
Animal Feed	34	26.5	28.8	2018–2019	Thailand [60]
Cattle feed	16	88	61.7	2018–2019	Kenya [61]
Pig feed	0				
Poultry feed	27	100	45.7	2019	Kenya [61]
Sheep feed	0				
TOTAL	77	26.5–100	61.7	2018–2019	

* Animal refers to samples for which the species was not indicated. ** n.i.: not indicated.

**Table 6:** Summary of the AF contamination data found in the literature (2019–2023).

Matrix	n	Prevalence (%)	Maximum Level (µg/kg)	Collection	Countries
AFB1					
Animal * feed	130	13–61	390	2016–2020	Brazil [66], Lithuania [73], Thailand [60]
Cattle feed	1012	3.9–100	374.6	2014–2020	China [77], Tunisia [86], Egypt [70], Thailand [87], Spain [71,75,82], South Africa [81], Kenya [61], India [74]
Pig feed	1048	3.1–100	59.7	2017–2020	China [77], Spain [68,82], Thailand [87]
Poultry feed	1036	13–99.9	760	2013–2020	China [77], Ethiopia [78], South Africa [67], Tunisia [86], Nigeria [64], Thailand [87], Spain [82], Kenya [61], Malaysia [76]
Sheep feed	116	12	6.1	2016–2020	Tunisia [86], Spain [82]
TOTAL	3342	3.1–100	760	2013–2020	
AFB2					
Animal feed	45	4	5.4	2016	Brazil [66]
Cattle feed	727	5–81	31.5	2014–2020	Tunisia [86], Egypt [70], Thailand [87], Spain [71,82], Shout Africa [81], Kenya [61], India [74]
Pig feed	428	1.3–14	4.1	2017–2020	Spain [68,82], Thailand [87]
Poultry feed	465	11–100	188	2013–2020	South Africa [67], Tunisia [86], Nigeria [64], Thailand [87], Spain [82], Kenya [61], Malaysia [76], Ethiopia [78]
Sheep feed	116	15	4.9	2016–2020	Tunisia [86], Spain [82]
TOTAL	1781	1.3–100	188	2013–2020	
AFG1					
Animal * feed	45	4	12	2016	Brazil [66]
Cattle feed	727	2.6–88	123	2014–2020	Tunisia [86], Egypt [70], Thailand [87], Spain [71,82], Shout Africa [81], Kenya [61], India [74]
Pig feed	428	0.9–10	6	2017–2020	Spain [68,82], Thailand [87]
Poultry feed	465	7–97	921.4	2013–2020	South Africa [67], Tunisia [86], Nigeria [64], Thailand [87], Spain [82], Kenya [61], Malaysia [76], Ethiopia [78]
Sheep feed	116	10	6.5	2016–2020	Tunisia [86], Spain [82]
TOTAL	1781	0.9–97	921.4	2013–2020	
AFG2					
Animal feed	45	0		2016	Brazil [66]
Cattle feed	727	1.3–44	28.5	2014–2020	Tunisia [86], Egypt [70], Thailand [87], Spain [71,82], Shout Africa [81], Kenya [61], India [74]
Pig feed	428	0–17	4.4	2017–2020	Spain [68,82], Thailand [87]
Poultry feed	465	2–82	221.4	2013–2020	South Africa [67], Tunisia [86], Nigeria [64], Thailand [87], Spain [82], Kenya [61], Malaysia [76], Ethiopia [78]
Sheep feed	116	16	4	2016–2020	Tunisia [86], Spain [82]
TOTAL	1781	0–82	221.4	2013–2020	
Total AFs					
Animal * feed	1850	1–65.7	66.7	2017–2021	Brazil [57], China [83,84,85], Rwanda [65]
Cattle feed	1750	2.4–59	406.1	2013–2021	India [74], Italy [58], Jordan [62], Rwanda [65]
Pig feed	2715	21–58	245.0	2015–2021	China [83,84,85], Taiwan [69]
Poultry feed	4414	7.4–97.5	1919.8	2017–2021	China [83,84,85], Ethiopia [78], Kenya [61], Malaysia [76], Pakistan [59], Rwanda [65]
Sheep feed	0				
TOTAL	10,729	1–97.5	1919.8	2013–2021	

* Animal refers to samples for which the species was not indicated.

**Table 7:** Summary of the FB contamination data found in the literature (2019–2023).

Matrix	n	Prevalence (%)	Maximum Level (µg/kg)	Collection	Countries
FB1					
Animal * feed	79	41.2–9	53,000	2016–2019	Brazil [66], Thailand [60]
Cattle feed	193	23.4–100	1494	2018–2019	Tunisia [86], Thailand [87], Kenya [61]
Pig feed	328	50–85	3959	2017	Spain [68], Thailand [87]
Poultry feed	262	96–100	7125.3	2013–2019	South Africa [67], Nigeria [64], Thailand [87], Kenya [61]
Sheep feed	0				Tunisia [86]
TOTAL	862	23.4–100	53,000	2013–2019	
FB2					
Animal feed	79	14.7–87	2800	2016–2019	Brazil [66], Thailand [60]
Cattle feed	193	19.5–94	677.3	2018–2019	Tunisia [86], Thailand [87], Kenya [61]
Pig feed	328	29.8–77	961	2017	Spain [68], Thailand [87]
Poultry feed	262	91–100	728.8	2013–2019	South Africa [67], Nigeria [64], Thailand [87], Kenya [61]
sheep feed	0				Tunisia [86]
TOTAL	862	14.7–100	2800	2013–2019	
Total FBs					
Animal feed	1860	45.4–93.4	17,490	2017–2021	Brazil [57], China [83,84,85], Rwanda [65]
Cattle feed	1284	100	11,638.2	2017–2019	Jordan [62], Kenya [61], Rwanda [65]
Pig feed	2715	50.4–99	13,254	2015–2021	China [83,84,85], Taiwan [69]
Poultry feed	4314	91–100	17,052	2017–2021	China [83,84,85], Kenya [61], Rwanda [65]
Sheep feed	0				
TOTAL	10,173	45.4–100	17,490	2015–2021	

* Animal refers to samples for which the species was not indicated.

**Table 8:** Summary of T-2 and HT-2 contamination data found in the literature (2019–2023).

Matrix	n	Prevalence (%)	Maximum Level (µg/kg)	Collection	Countries
T-2					
Animal * feed	817	21.3–31.3	246.7	2016–2021	Brazil [57], China [84], Lithuania [73]
Cattle feed	239	2–13	1734.6	2016–2019	Tunisia [86], Thailand [87], Kenya [61], Jordan [62]
Pig feed	452	0.9–1	35.9	2017–2020	China [84], Spain [68], Thailand [87]
Poultry feed	545	4–100	956.5	2015–2020	China [84], South Africa [67], Tunisia [86], Thailand [87], Kenya [61]
Sheep feed	16	n.i. **	n.i.	2016–2017	Tunisia [86]
TOTAL	2069	0.9–100	1734.6	2015–2021	
HT-2					
Animal feed	0				
Cattle feed	151	1–37	173.4	2016–2019	Tunisia [86], Thailand [87], Kenya [61]
Pig feed	328	0.9–7	123	2017	Spain [68], Thailand [87]
Poultry feed	275	4–100	119.8	2015–2019	South Africa [67], Tunisia [86], Thailand [87], Kenya [61]
Sheep feed	16	13	13.1	2016–2017	Tunisia [86]
TOTAL	770	0.9–100	173.4	2015–2019	

* Animal refers to samples for which the species was not indicated. ** n.i.: not indicated.

**Table 9:** Summary of NIV, NEO, DAS, and STER contamination data found in the literature (2019–2023).

Matrix	n	Prevalence (%)	Maximum Level (µg/kg)	Collection	Countries
NIV					
Animal * feed	0				
Cattle feed	228	5.2–94	117.5	2016–2019	Tunisia [86], Thailand [87], South Africa [81], Kenya [61]
Pig feed	100	18	165.4	n.i. **	Thailand [87]
Poultry feed	200	23.3–96	647	2013–2019	Tunisia [86], Nigeria [64], Thailand [87], Kenya [61]
Sheep feed	16	n.i.	n.i.	2016–2017	Tunisia [86]
TOTAL	544	5.2–96	647	2013–2019	
NEO					
Animal feed	0				
Cattle feed	135	n.i.	n.i.	2016–2017	Tunisia [86], Thailand [87]
Pig feed	100	n.i.	n.i.	n.i.	Thailand [87]
Poultry feed	143	n.i.	n.i.	2016–2017	Tunisia [86], Thailand [87]
Sheep feed	16	n.i.	n.i.	2016–2017	Tunisia [86]
TOTAL	394	n.i.	n.i.	2016–2017	
DAS					
Animal feed	0				
Cattle feed	212	1	4.4	2016–2019	Tunisia [86], Thailand [87]
Pig feed	100	2	5.1	n.i.	Thailand [87]
Poultry feed	143	3–14	219.2	2016–2017	Tunisia [86], Thailand [87]
Sheep feed	16	n.i.	n.i.	2016–2017	Tunisia [86]
TOTAL	471	1–14	219.2	2016–2019	
STER					
Animal feed	0				
Cattle feed	177	6–45.5	139.1	2018–2020	South Africa [81], Spain [82]
Pig feed	328	2.2–10	308	2017–2020	Spain [68,82]
Poultry feed	100	7	5.1	2019–2020	Spain [82]
Sheep feed	100	5	5.6	2019–2021	Spain [82]
TOTAL	705	2.2–45.5	308	2017–2021	

* Animal refers to samples for which the species was not indicated. ** n.i.: not indicated.

**Table 11:** Principal biomarkers found in animal plasma, urine, feces, and excreta after oral or intravenous mycotoxin administration in poultry and pigs.

	Poultry				Pigs			
Mycotoxin	Plasma Serum or Blood	Urine	Feces	Excreta	Plasma Serum or Blood	Urine	Feces	Excreta
AFB1	**AFB1** AFL	AFB1-N7-Gua		**AFB1**		**AFB1**, AFM1, AFB2		
OTA	**OTA**, OTα			**OTA**, OTα	OTA	**OTA**, OTα	OTA, OTα	**OTA**, OTα
DON	**DON-s**			**DON-s**	DON, DOM-1 **DON-GlcA**	**DON**, DON-GlcA		
ZEA	**ZEA**, α-ZEL, β-ZEL, ZEA-GlcA	**ZEA**, α-ZEL, β-ZEL, ZEA-GlcA	**ZEA**, α-ZEL, β-ZEL, ZEA-GlcA		ZEA, **ZEA-GlcA**	ZEA, **ZEA-GlcA**	**ZEA**, α-ZEL	
FB1	**FB1**, pHFB1				**FB1** HFB1	**FB1** HFB1	**FB1** HFB1, pHFB1	
CIT	CIT, DH-CIT				CIT, DH-CIT			

AFB1: aflatoxin B1; AFB2: aflatoxin B2; AFL: aflatoxicol; AFM1: aflatoxin M1; DOM-1: deepoxy-deoxynivalenol; DON: deoxynivalenol; 3-ADON: 3-acetyldeoxynivalenol; 15-ADON: 15-acetyldeoxynivalenol; DON-3GlcA: deoxynivalenol-3-glucuronide; DON-GlcA: DON-glucuronides; DON-s: deoxynivalenol sulfate; FB1: fumonisin B1; HFB1: hydrolyzed FB1; pHFB1: partially hydrolyzed FB1; OTα: ochratoxin α; OTA: ochratoxin A; ZAL: zearalanol; α-ZAL: α-zearalanol; β-zearalanol; ZEA: zearalenone; α-ZEL: α-zearalenol; β-ZEL: β-zearalenol; ZEA-GlcA: ZEA-glucuronides. In bold most prevalent biomarkers.

## Data Availability

Not applicable.

## Supplementary Materials

The following are available online at https://www.mdpi.com/article/10.3390/toxins16050218/s1, Table S1: Analytical methods employed for mycotoxin analysis in feed. Table S2: Levels of mycotoxins in feed obtained from the bibliographic search (2019–2023). Table S3: Analytical methods employed for the analysis of mycotoxin biomarkers in animal biological fluids.

## Abbreviations

15-ADON	15-acetyldeoxynivalenol
3-ADON	3-acetyldeoxynivalenol
ABM	Animal biomonitoring
ACN	Acetonitrile
AFB1	Aflatoxin B1
AFB2	Aflatoxin B2
AFG1	Aflatoxin G1
AFG2	Aflatoxin G2
AFL	Aflatoxicol
AFM1	Aflatoxin M1
AFP1	Aflatoxin P1
AFQ1	Aflatoxin Q1
AFs	Aflatoxins
AME	Alternariol monomethyl ether
AOH	Alternariol
BEA	Beauvericin
CIT	Citrinin
DAS	Diacetoxyscirpenol
DH-CIT	Dihydrocitrinone
DOM-1	Deepoxidesoxynivalenol
DON	Deoxynivalenol
DON-3GlcA	DON-3-glucuronide
DON-15GlcA	DON-15-glucuronide
DON-3gluc	DON-3 glucoside
DON-s	DON sulfate
EFSA	European Food Safety Authority
ELISA	Enzyme-linked immunosorbent assay
ENNA	Enniatin A
ENNA1	Enniatin A1
ENNB	Enniatin B
ENNB1	Enniatin B1
ERGOT	ergot alkaloids
EtOAc	Ethyl acetate
EU	European Union
FB1	Fumonisin B1
FB2	Fumonisin B2
FB3	Fumonisin B3
FB4	Fumonisin B3
FBs	Fumonisins
FLD	Fluorescence detector
FUS-X	Fusarenon-X
HBM	Human biomonitoring
GlcA	Glucuronide
HFBx	Hydrolyzed FBx
HT-2	HT-2 toxin
IAC	Immunoaffinity column
IARC	International Agency for Research on Cancer
LC	Liquid chromatography
LLE	Liquid-liquid extraction
LOQ	Limit of quantification
MeOH	Methanol
MON	Moniliformin
MS	Mass spectrometer
MS/MS	Tandem mass spectrometry
NEO	Neosolaniol
NIV	Nivalenol
OTA	Ochratoxin A
OTα	Ochratoxin α
OTB	Ochratoxin B
PAT	Patulin
pHFBx	Partially hydrolyzed FBx
QuEChERs	Quick, easy, cheap, effective, rugged, and safe
ROQC	Roquefortine C
SLE	Solid–liquid extraction
SPE	Solid phase extraction
STER	Sterigmatocystin
T-2	T-2 toxin
TENT	Tentoxin
ZAL	Zearalanol
α-ZAL	α-zearalanol
β-ZAL	β-zearalanol
ZAN	Zearalanone
ZAN-14GlcA	ZAN-14-glucuronide
ZEA	Zearalenone
α-ZEL	α-zearalenol
β-ZEL	β-zearalenol
ZEL-14GlcA	ZEA-14-glucuronide
ZEL-16GlcA	ZEA-16-glucuronide
