# Supplementary material for: Monitoring Mycotoxin Exposure in Food-Producing Animals (Cattle, Pig, Poultry, and Sheep)
Source: Toxins (Basel). 2024 May 9;16(5):218. doi: 10.3390/toxins16050218 (PMC11125880; doi:10.3390/toxins16050218)
Supplement: Supplementary file 1 [file toxins-16-00218-s001.zip › toxins-2978810-supplementary.pdf]

Supplementary Materials: Monitoring Mycotoxin Exposure in Food-Producing Animals (Cattle, Pig, Poultry, and Sheep)

Borja Muñoz-Solano, Elena Lizarraga Pérez and Elena González-Peñas *

Department of Pharmaceutical Sciences, Faculty of Pharmacy and Nutrition, Universidad de Navarra, 31008 Pamplona, Spain; bmunoz.1@alumni.unav.es (B.M.-S.); elizarraga@unav.es (E.L.P.)
* Correspondence: mgpenas@unav.es

Table S1. Analytical methods employed for mycotoxin analysis in feed.

Matrix	Analyte/s	LOQ (µg/kg)	Sample preparation	Separation and detection technique	year	Ref
Pig feed	Total AFs, ZEA, Total FBs	1-250	SLE<br>20 g + 100 mL of 70% MeOH (mix 25 °C, 3 min). Whatman No. 1 filtration.<br>AFs: measured directly.<br>ZEA: dilute 1:4 with 70% MeOH<br>FBs: dilute 1:19 with H_2O	ELISA<br>AgraQuant Total Aflatoxin Assay 1/20<br>AgraQuant Total Fumonisin Assay 0.25/5.0<br>AgraQuant Zearalenone Plus Assay 25/1000	2019	[1]
Pig feed	DON	25	SLE<br>20 g + 100 mL of H_2O (mix 25 °C, 3 min). Whatman No. 1 filtration.<br>Dilute 1:4 with H_2O	ELISA<br>AgraQuant Deoxynivalenol Assay 0.25/5.0	2019	[1]
Cattle, poultry, and animal feed	AFs, FBs	n.i*	SLE<br>20 g + 40 mL of 90% MeOH (mix 2 min). Whatman No. 1 filtration. pH adjusted to 7.0 (25%NaOH or 2M HCl).<br>FBs: measured directly.<br>AFs: dilute 1 mL with 1.5 mL 57% MeOH	ELISA<br>Helica Biosystems	2019	[2]
Poultry and cattle feed	AFB1, AFB2, AFG1, AFG2, OTA, FB1, FB2, ZEA, DON	0.6-18	SLE<br>1 g + 4 mL ACN/ H_2O/Acetic acid (80:20:1) + vortex 1 min + orbital shaker 60 min + centrifugation 1500 g 5 min + filtration 0.22 µm PTFE.<br>80 µL filtrate + 20 µL IS	LC-MS/MS<br>Column: RaptorTM ARC-18 (2.1 × 100 mm, 2.7 µm) at 40ºC<br>Flow: 0.2 mL/min<br>Mobile phase: (A) 0.1% formic acid in H_2O; (B) 0.1% formic acid in ACN: MeOH (50:50, v/v) in gradient conditions<br>Detector: QqQ, ESI (±), MRM	2019	[3]
Poultry feed	ZEA, α-ZEL, β-ZEL, T-2, FB1, FB2, FB3, AFB1, AFB2, AFG1, AFG2, HT-2, AME, DON, 3-ADON, 15-ADON, OTA	0.1-63.9	SLE<br>10 g + 40 mL ACN/ H_2O /formic acid (79/20/1) + mix 60 min 180 rpm horizontal shaker + centrifugation + filtration 0.22 µm PTFE	LC-MS/MS<br>Column: BEH C18 (2.1 × 50 mm, 1.7 µm) at 40ºC<br>Flow: 0.6 mL/min<br>Mobile phase: (A) H_2O; (B) ACN, both containing 0.1% of acetic acid and ammonium acetate at 5 mM in gradient conditions<br>Detector: QqQ, ESI (+), MRM	2019	[4]

Table S1. Analytical methods employed for mycotoxin analysis in feed (continuation)

Matrix	Analyte/s	LOQ (µg/kg)	Sample preparation	Separation and detection technique	year	Ref
Poultry, cattle and sheep feed	AFB1, AFB2, AFG1, AFG2, AME, AOH, TENT, OTA, BEA, ENNA, ENNA1, ENNB, ENNB1, DON, 15-ADON, 3-ADON, NIV, NEO, DAS, T-2, HT-2, ZEA.	0.3-225.5	QuEChERs<br>2-5 g + 10 mL ACN/ H_2O formic acid (79/20/1) + mix 5 min horizontal shaker + centrifugation at 4000 rpm 5 min 5ºC + QuEChERs (4 g of $MgSO_4$ and 1 g of NaCl) + 1 min shake + centrifugation 4000 rpm 10 min.<br>2 mL supernatant + 100 mg C18 (or PSA) + 600 mg $MgSO_4$ + 1 min shake + centrifugation at 4000 rpm 10 min + filtration 0.22 µm PTFE	LC-MS/MS<br>Column: Gemini C18 (2 × 150 mm, 2 µm) at 25ºC<br>Flow: 0.25 mL/min<br>Mobile phase: (A) MeOH; (B) H_2O, both containing 0.1% formic acid and 5 mM ammonium formate. Gradient conditions<br>Detector: QTRAP, ESI (+), MRM	2019	[5]
Cattle feed	AFB1, AFB2, AFG1, AFG2, OTA, ZEA	0.06-2.8	IAC<br>25 g + 5 g NaCl + ACN/ H_2O (80/20) + mix 2 min at high speed + filtration.<br>10 mL extract + 40 mL PBS (pH 7.4) 0.01% tween 20 + vortex + filtration.<br>20 mL of extract through a multiple-mycotoxin immunoaffinity column (AOZ IAC)	LC-FLD<br>Column: ACE C18 (250 × 4.6 mm, 5 µm) at 40ºC<br>Post column derivatization: UV light 254 nm<br>Flow: 0.8 mL/min<br>Mobile phase: MeOH/ H_2O/ACN (22/62/16)<br>Detector: 360/460 nm (λex/λem)	2019	[6]
Pig feed	AFB1, AFB2, AFG1, AFG2	1	SLE<br>2 g + 10 mL ACN + 3 min vortex + 5 min centrifugation at 4500 rpm.<br>2 mL supernatant evaporated to dryness under N_2 + reconstituted with 1 mL MeOH/ H_2O (50/50)	LC-FLD<br>Column: C18 Kinetex separation (150 × 4.6 mm, 2.6 µm) at 30ºC<br>Flow: 0.8 mL/min<br>Mobile phase: (A) H_2O, (B) MeOH, (C) ACN, in gradient conditions<br>Detector: 365/460 nm (λex/λem)	2019	[7]
Pig feed	FB1, FB2, ZEA, OTA, CIT, T-2, HT-2, DON, FUS-X, STER, ENNA, ENNA1, ENNB, ENNB1, BEA	2.0-136	QuEChERs<br>2 g + 8 mL H_2O + 1 min vortex + 10 mL ACN/formic acid (95/5) + 3 min vortex + 4 g $MgSO_4$ + 1g NaCl + 2 min shake + 5 min centrifugation 4500 rpm.<br>2 mL supernatant evaporated to dryness under N_2 + reconstituted with 1 mL MeOH/ H_2O (50/50)	LC-MS/MS<br>Column: C18 Zorbax Eclipse Plus RRHD (50 × 2.1 mm, 1.8 µm) at 35ºC<br>Flow: 0.4 mL/min<br>Mobile phase: (A) H_2O; (B) MeOH, both containing 0.3% formic acid and 5mM ammonium formate, in gradient conditions<br>Detector: QqQ, ESI (+), MRM	2019	[7]

Table S1. Analytical methods employed for mycotoxin analysis in feed (continuation)

Matrix	Analyte/s	LOQ (μg/kg)	Sample preparation	Separation and detection technique	year	Ref
Poultry feed	AFB1, AFB2, AFG1, AFG2, AFM1, AOH, AOH methyl ether, BEA, CIT, Cyclopiazonic acid, DON, FB1, FB2, FB3, FB4, Hydrolyzed FB1, MON, NIV, OTA, OTB, Tenuazonic acid, ZEA, ZEA-sulfate	n.i.	SLE<br>5 g + 20 mL ACN/ H_2O /formic acid (79/20/1) + 90 min rotatory shaker + 20 mL ACN/ H_2O /formic acid (20/79/1)	LC-MS/MS<br>Column: Gemini C18 (4.6 × 150 mm, 5 μm) at 25ºC<br>Flow: 0.25 mL/min<br>Mobile phase: (A) MeOH / H_2O /acetic acid (10:89:1); (B) MeOH / H_2O /acetic acid (97:2:1) both containing 5 mM ammonium acetate. in gradient conditions<br>Detector: QTRAP, ESI (±), MRM	2019	[8]
Animal Feed	AFB1	n.i.	SPE<br>5 g + 20 mL ACN/ H_2O (90/10) + vortex 10 min + centifuge 1895g 5 min + filtration + 6 mL extract passed through SPE + 4 mL extract evaporated N_2 + reconstituted 400 μL H_2O /TFA/acetic acid (35/10/5) + vortex 10 s + heat 65ºC 15 min + 20 h at room temperature	LC-FLD<br>Column: not indicated<br>Flow: 1.0 mL/min<br>Mobile phase: (A) H_2O (B) ACN<br>Detector: FLD	2019	[9]
Animal Feed	AFB1	n.i.	SLE<br>5 g + 25 mL MeOH 70% + shake 5 min + filtration + 1 mL extract diluted with 1 mL H_2O	ELISA<br>Aflatoxin kit r-biopharm®	2019	[9]
Cattle feed	AFB1	0.007-0.3	SLE<br>0.5 g + 10 mL MeOH 80% + vortex 20 min + centrifuge 3.5xg 5 min + supernatant + 6 mL n-hexane + vortex 30s + centrifuge 3.5xg 5 min + bottom layer dried at 45ºC + reconstitute with 10 mL MeOH/ H_2O (1/9) + 100 μL 1-octanol + 100 μL toluene + vortex 45s + 15% (w/v) Na_2SO_4. Collect upper organic phase + dried 20 μL of organic phase + reconstituted in 20 μL buffer	Capillary Electrophoresis-Laser Induced Fluorescence<br>Column: uncoated fused-silica capillary (44 cm x 50 μm)	2019	[10]

Table S1. Analytical methods employed for mycotoxin analysis in feed (continuation)

Matrix	Analyte/s	LOQ (µg/kg)	Sample preparation	Separation and detection technique	year	Ref
Animal Feed	AFB1, DON, FB1, FB2, OTA, T-2, HT-2, ZEA	0.71-12.6	SLE<br>1 g + 4 mL ACN/ H_2O / formic acid (79/20/1) + shake 30 min + centrifuge 3500 rpm 15 min + 0.1 mL extract evaporated N_2 40ºC + reconstituted in 0.05 mL of MeOH /0.01 M ammonium acetate (5:95, v/v) and 0.05 mL MeOH /0.01 M ammonium acetate (95:5, v/v) + centifuge 14800 rpm 30 min	LC-MS/MS<br>Column: Kinetex Biphenyl (100 × 2.1 mm, 2.6 µm) at 40ªC<br>Flow: 0.3 mL/min<br>Mobile phase: (A) 0.01 M ammonium acetate and 0.1% of acetic acid in H_2O /MeOH (95:5, v/v); (B) 0.01 M ammonium acetate and 0.1% of acetic acid in H_2O /MeOH (5:95, v/v),<br>in gradient conditions<br>Detector: QqQ, ESI (±), MRM	2019	[11]
Animal Feed	AFB1, DON, FB1, FB2, OTA, T-2, HT-2, ZEA	0.38-8.41	IAC<br>1 g + 4 mL ACN/ H_2O / formic acid (79/20/1) + shake 30 min + centifuge 3500 rpm 15 min + 0.5 mL extract + 6 mL PBS + centifuge 3500 rpm 10 min + 2 mL diluted extract passed through IAC + elution with 1.5 mL MeOH + dried N_2 40ºC + reconstitute in mobile phase	LC-MS/MS<br>Column: Kinetex Biphenyl (100 × 2.1 mm, 2.6 µm) at 40ªC<br>Flow: 0.3 mL/min<br>Mobile phase: (A) 0.01 M ammonium acetate and 0.1% of acetic acid in H_2O /MeOH (95:5, v/v); (B) 0.01 M ammonium acetate and 0.1% acetic acid in H_2O /MeOH (5:95, v/v),<br>in gradient conditions<br>Detector: QqQ, ESI (±), MRM	2019	[11]
Cattle feed	AFs	0.05	SLE<br>2 g + 5 mL of 70% MeOH + vortex 5 min + centifuge at 10000 g 10 min at room temperature.<br>0.5 mL + 0.5 mL H_2O	ELISA	2020	[12]
Cattle feed	T-2	0.05	SLE<br>1 g + 20 mL of 90% MeOH + vortex 5 min + centifuge at 10000 g 5 min at room temperature. 1 mL + 5 mL H_2O	ELISA	2020	[12]

Table S1. Analytical methods employed for mycotoxin analysis in feed (continuation)

Matrix	Analyte/s	LOQ (μg/kg)	Sample preparation	Separation and detection technique	year	Ref
Cattle feed	DON	10	SLE<br>2 g + 20 mL of 90% MeOH + vortex 5 min + centifuge at 10000 g 10 min at room temperature.<br>0.5 mL + 0.5 mL H_2O	ELISA	2020	[12]
Cattle feed	ZEA	0.3	SLE<br>2 g + 8 mL of 90% MeOH + vortex 5 min + centifuge at 10000g 10 min at room temperature.<br>0.5 mL + 2 mL H_2O	ELISA	2020	[12]
Cattle feed	FB	0.5	SLE<br>1 g + 5 mL of 70% MeOH + vortex 5 min + centifuge at 10000 g 10 min at room temperature.<br>0.1 mL + 1.9 mL H_2O	ELISA	2020	[12]
Pig, poultry and cattle feed	AFB1, AFB2, AFG1, AFG2, T-2, HT-2, OTA, FB1, FB2, DAS, 15-ADON, NEO, FUS-X, ZEA, DON, NIV, 3-ADON	1-40	QuEChERs<br>1 g + 10 mL H_2O /formic acid (99/1) + shake 30 min + 10 mL ACN + shake 30 min + 4 g $MgSO_4$ + 1 g NaCl + shake 30 s + centifuge 5 min at 10000 rpm<br>2 mL + 300 mg $MgSO_4$ + 100 mg C18 + mix and centifuge + evaporation to dryness + reconstitute with 960 μL 20% MeOH and 40 μL IS	LC-MS/MS<br>Column: Accucore analytical (100 × 2.1 mm x 2.6 μm) at 25ºC<br>Flow: 0.4 mL/min<br>Mobile phase: (A) MeOH, (B) H_2O, both containing 5 mM ammonium formate and 0.1% formic acid; (C) H_2O, (D) MeOH, both containing 5 mM ammonium acetate and 0.1% acetic acid, in gradient conditions<br>Detector: QqQ, ESI (±), MRM	2020	[13]
Cattle and poultry feed	AFB1, AFB2, AFG1, AFG2, AFM1, AFs, DON, DON-3gluc, NIV, FA1, FA2, FB1, FB2, FB3, FB4, FBs, OTA, ERGOT, HT-2, T-2, ZEA	n.i.	SLE<br>5 g + 20 mL ACN/ H_2O /formic acid (79/20/1) + 90 min rotatory shaker.<br>1 mL + 1 mL ACN/ H_2O /formic acid (20/79/1)	LC-MS/MS<br>Column: Gemini C18 (4.6 × 150 mm, 5 μm) at 25ºC<br>Flow: 0.25 mL/min<br>Mobile phase: (A) MeOH / H_2O /acetic acid (10:89:1); (B) MeOH / H_2O /acetic acid (97:2:1), both containing 5 mM ammonium acetate, in gradient conditions<br>Detector: QTRAP, ESI (±), MRM	2020	[14]

Table S1. Analytical methods employed for mycotoxin analysis in feed (continuation)

Matrix	Analyte/s	LOQ (µg/kg)	Sample preparation	Separation and detection technique	year	Ref
Cattle and animal Feed	AFB1, AFB2, AFG1, AFG2	0.05-1	IAC<br>5 g + 40 mL ACN/ H_2O (90:10) + ultrasonic bath 10 min + centifuge 10 min 4676 g.<br>3 mL + 72 mL PBS + filter through an IAC column (Easi-extract® Aflatoxin)	LC-FLD<br>Column: Poroshell 120 EC-C18 UHPLC (4.6 × 50 mm, 2.7 µm) at 40ºC<br>Post column derivatization: UV light 254 nm<br>Flow: 1.2 mL/min<br>Mobile phase: MeOH/ H_2O / ACN (20/70/10)<br>Detector: 365/440 nm (λex/λem)	2020	[15]
Animal feed	AFB1, AFB2, AFG1, AFG2	0.5	IAC<br>12.5 g + 12.5 mL H_2O + mix + 100 mL (MeOH/ H_2O 8:2) + 5 g NaCl + shake 30 min + centifuge 2800 g 5 min<br>3 mL + 12 mL H_2O + 0.1 mL tween 20 + pass through Aflatest® IAC column<br>1 mL evaporated and reconstituted in 0.5 mL MeOH/H20 1:1 0.5 % acetic acid.	LC-FLD<br>Column: UPLC BEH amide C18 (2.1x 100 mm x 1.7 µm) at 40ºC<br>Flow: 0.4 mL/min<br>Mobile phase: (A) H_2O 0.1% acetic acid (B) MeOH (C) ACN (64:18:18)<br>Detector: 365/456nm (λex/λem)	2020	[16]
Animal feed	AFB1, AFB2, AFG1, AFG2, OTA, OTB, ZEA	0,63-42	SPE<br>0.5 g + 5 mL ACN/ H_2O /orthophosphoric acid (79/20/1) + vortex 1h + centifuge 5500 rpm 10 min.<br>2 mL through Oasis Prime HLB SPE. 0.5 mL eluate + 0.5 mL chloroform + vortex 10 s + injection of 5 mL H_2O + vortex 30 s + centrifugation 7000 rpm 5 min.<br>0.2 mL organic phase evaporated and reconstituted in 0.2 mL mobile phase	LC-FLD<br>Column: Cortecs T3 C18 (150 × 4.6 mm, 2.7 µm) at 40ºC.<br>Post column derivatization: UV light 254 nm<br>Flow: 1.4 mL/min<br>Mobile phase: (A) H_2O 0.1% ortophosphoric acid (B) ACN (C) MeOH, in gradient conditions.<br>Detector: 365/440 nm (λex/λem) for AFs, 234/469 nm (λex/λem) for Ochratoxins and ZEA	2020	[17]
Animal feed	AFB1, ZEA, T-2	0.2-20	IAC<br>Sample + MeOH/ H_2O (75:25) for AFs and ZEA, (60:40) for T-2 + shaker 60 min at 23ºC + centrifuge 10 min 3468 g<br>Filter + PBS + through 11+Myco MS-PREP® IAC column	LC-FLD<br>Column: LiChrospher 100 RP-18, LiChroCART 250- 4 (250 × 4.0 mm, 5 µm); at 30ºC<br>Flow: 1 mL/min<br>Mobile phase: AFs: MeOH/ H_2O / ACN (30/60/20). T-2: H_2O / ACN (40/60). ZEA: MeOH/ H_2O / ACN (46/46/8)<br>Detector: AFs 365/435 nm (λex/λem). T-2 381/470 nm (λex/λem). ZEA 274/418 nm(λex/λem).	2021	[18]

Table S1. Analytical methods employed for mycotoxin analysis in feed (continuation)

Matrix	Analyte/s	LOQ (µg/kg)	Sample preparation	Separation and detection technique	year	Ref
Animal feed	DON	1.4	IAC<br>Sample + H_2O + shaker 60 min at 23ºC + centrifuge 10 min 3468 g<br>Filter + PBS + through 11+Myco MS-PREP® IAC column	LC-UV<br>Column: LiChrospher 100 RP-18, LiChroCART 250- 4 (250 × 4.0 mm, 5 µm), at 30ºC<br>Flow: 1 mL/min<br>Mobile phase: MeOH/ H_2O / ACN (3/94/3).<br>Detector: UV 218 nm	2021	[18]
Cattle feed	AFB1, AFB2, AFG1, AFG2, AFs	0.19-2.75	IAC<br>25 g + 5 g NaCl + 125 mL MeOH/ H_2O (70:30) + 30 min orbital shake + filter using whatman nº 1.<br>15 mL + 30 mL H_2O. 15 mL pass through AflaPureTM IAC column	ELISA + LC-FLD<br>Column: Waters Spherisorb C- 18 (250 × 4.6 mm, 5 µm), at 40ºC<br>Pre column derivatization: Hexane + TFA<br>Flow: 1 mL/min<br>Mobile phase: MeOH/ H_2O / ACN (20/60/20)<br>Detector: 365/435 nm (λex/λem)	2021	[19]
Animal feed	AFB1, DON, DON-3-glucoside, FB1, FB2, FB3, ZEA	0.19-5.77	SLE<br>5 g + 20 mL ACN/ H_2O / acetic acid (79:20:1) + 90 min rotatory shaker.<br>500 µL + 500 µL ACN/ H_2O /acetic acid (20:79:1)	LC-MS/MS<br>Column: Gemini C18 (4.6 × 150 mm, 5 µm), at 25ºC<br>Flow: 0.25 mL/min<br>Mobile phase: (A) MeOH / H_2O /acetic acid (10:89:1); (B) MeOH / H_2O /acetic acid (97:2:1), both containing 5 mM ammonium acetate, in gradient conditions<br>Detector: QTRAP, ESI (±), MRM	2021	[20]
Cattle feed	AFB1	0.1	IAC<br>25 g + 2.5 g NaCl + 50 mL MeOH 80% + mix 1 min + filter through whatman nº 4.<br>10 mL + 40 mL H_2O. 10 mL through AflaTest WB SR IAC column	LC-FLD<br>Column: Ace 5 C18, (250 × 4.6 mm, 5 µm)<br>Post column derivatization: UV light 254 nm<br>Flow: 1 mL/min<br>Mobile phase: MeOH/ H_2O / ACN (40/50/10)<br>Detector: 365/435 nm (λex/λem)	2021	[21]
Poultry feed	AFB1, AFB2, AFG1, AFG2, AFs	0.3-1.86	IAC<br>50 g + 5 g NaCl + 100 mL MeOH/ H_2O (80/20) + filter. 10 mL + 40 mL H_2O. 15 mL pass through AflaTest WB SR IAC column. Elution with 1 mL MeOH and 1 mL H_2O	LC-FLD<br>Column: C18 (25 0 × 4.6 mm, 5 µm)<br>Post column derivatization: UV light 254 nm<br>H_2O: 0.6 mL/min<br>Mobile phase: eOH/ ACN (55/35/10)<br>Detector: 365/435 nm (λex/λem)	2021	[22]

Table S1. Analytical methods employed for mycotoxin analysis in feed (continuation)

Matrix	Analyte/s	LOQ (µg/kg)	Sample preparation	Separation and detection technique	year	Ref
Pig, poultry and cattle feed	AFB1	0.5	IAC<br>25 g + 100 mL MeOH/ H_2O (80/20) + SPE clean up. Dilute with PBS + pass through AokinImmunoClean CF AFLA IAC colum. Elution with MeOH. Evaporate to dryness using N_2. Reconstitution in mobile phase.	LC-FLD<br>Column: Gemini C18 (250 × 4.6 mm, 5 µm), at 30ºC<br>Flow: 1 mL/min<br>Mobile phase: MeOH/ H_2O / ACN (30/60/10)<br>Detector: 360/435 nm (λex/λem)	2021	[23]
Pig, poultry and cattle feed	DON	100	IAC<br>25 g + 100 mL MeOH/ H_2O (60/40) + SPE clean up. Dilute with PBS + pass through AokinImmunoClean CF DON IAC column. Elution with MeOH. Evaporate to dryness using N_2. Reconstitution in mobile phase.	LC-UV<br>Column: ZORBAX Eclipse XDB-C18 (150 × 4.6 mm, 5 µm), at 30ºC<br>Flow: 0.8 mL/min<br>Mobile phase: MeOH/ H_2O (20/80)<br>Detector: 218 nm	2021	[23]
Pig, poultry and cattle feed	ZEA	10	IAC<br>25 g + 100 mL MeOH/ H_2O (84/16) + SPE clean up. Dilute with PBS + pass through ZearaStar IAC column + elution with MeOH. Evaporate to dryness using N_2. Reconstitution in mobile phase.	LC-FLD<br>Column: ZORBAX Eclipse XDB-C18 (150 × 4.6 mm, 5 µm) at 30ºC<br>Flow: 0.8 mL/min<br>Mobile phase: MeOH/ H_2O / ACN (8/46/46)<br>Detector: 274/440 nm (λex/λem)	2021	[23]
Cattle feed	NIV, DON, 3-ADON, 15-ADON, ZEA, AFG2, AFG1, AFB2, AFB1, DAS, AOH, FB1, FB2, FB3, OTA, AME, STER, ROQC, ENN B	1.7-317.7	SPE<br>5 g + IS (ZAN, DOM) + 20 mL ACN/ H_2O / acetic acid (79/20/1) + shake 1 h + centrifuge 3300g 15 min + SPE clean up + eluate with 25 mL solvent + 10 mL n-hexane + second SPE clean up. Evaporate to dryness using N_2. Reconstitution in 150 µL mobile phase	LC-MS/MS<br>Column: Symmetry C18 (150 × 2.1 mm, 5 µm) at 25ºC<br>Flow: 0.3 mL/min<br>Mobile phase: (A) H_2O /MeOH/Acetic acid (94/5/1); (B) H_2O/MeOH/Acetic acid (2/97/1), both containing 5 mM ammonium acetate, in gradient conditions<br>Detector: QqQ, ESI (+), MRM	2021	[24]

Table S1. Analytical methods employed for mycotoxin analysis in feed (continuation)

Matrix	Analyte/s	LOQ (μg/kg)	Sample preparation	Separation and detection technique	year	Ref
Animal feed	AFB1, AFB2, AFG1 AFG2, OTA, ZEA, DON, FB1, FB2, T-2, HT-2	0.5-500	QuEChERs<br>5 g + 10 mL H_2O 0.1% formic acid + 10 mL ACN + shake 4000 rpm 30 min + 4g $MgSO_4$ + 1 g NaCl + shake 4000 rpm 1 min + centrifuge 4000 rpm 10 min.<br>1 mL + 25 mg C18 + 25 mg PSA + centrifuge 10000 rpm 5 min.<br>0.4 mL + 0.5 mL H_2O + 0.5 mL ACN	LC-MS/MS<br>Column: Imtakt Cardenza CD-C18 UP (150 × 2.0 mm, 3.0 μm) at 40ºC<br>Flow: 0.4 mL/min<br>Mobile phase: (A) H_2O 0.1% formic acid (B) ACN 0.1% formic acid, both containing 5 mM ammonium formate, in gradient conditions<br>Detector: QqQ, ESI (+), MRM	2021	[25]
Poultry feed	AFs	n.i.	SLE<br>5 g + 25 mL MeOH + filtration	ELISA<br>Neogen Veratox® Corporation USA	2022	[26]
Poultry feed	AFB1, AFB2, AFG1, AFG2, AFs	0.5	IAC<br>20 g + 2 g NaCl + 100 mL MeOH/ H_2O (80/20) + 50 mL n-hexane + blend 5 min + filtration. 40 mL + 86 mL PBS + filtration. Pass through AflaCLEANTM IAC and elute with 2 mL MeOH	LC-FLD<br>Column: ZORBAX SB-C18 (150× 4.6 mm, 3.5 μm) at 35ºC<br>Flow: 1 mL/min<br>Mobile phase: H_2O / ACN / MeOH (60/25/15)<br>Detector: 360/440 nm (λex/λem)	2022	[27]
Animal feed	AFB1	2	SLE<br>20 g + 100 mL MeOH/ H_2O (70/30) + shake 1 h + filtration	ELISA<br>BIO-SHIELD B1 (ProGnosis biotech)	2022	[28]
Pig and poultry feed	AFs, Trichothecenes type B, FBs, ZEA	0.5-10	SPE<br>25 g + 100 mL ACN/ H_2O (50/50) + blend 1 h + filtration. 2 mL + 0.1 mL acetic acid. 750 μL to SPE. Centifuge 10000 rpm 1 min	LC-MS/MS<br>Column: Gemini C18 (150 × 4.6 mm, 5 μm) at 40ºC<br>Flow: 1 mL/min<br>Mobile phase: (A) H_2O /acetic acid (199/1); (B) MeOH /acetic acid (199/1), both containing 2 mM ammonium acetate, in gradient conditions<br>Detector: QTRAP, ESI (±), SRM	2022	[29]
Animal feed	DON	250	SLE<br>20 g + 100 mL H_2O + shake 1 h, 150 rpm, 25ºC + filtration and dilution 1:4	ELISA<br>AgraQuant® ELISA kits	2022	[30]

Table S1. Analytical methods employed for mycotoxin analysis in feed (continuation)

Matrix	Analyte/s	LOQ (µg/kg)	Sample preparation	Separation and detection technique	year	Ref
Animal feed	AFs, DON, FBs, OTA, T-2, ZEA	1-250	SLE<br>20 g + 100 mL MeOH/ H_2O (70/30) + shake 1 h 150 rpm 25ºC. Filtrate<br>Dilution 1:20 with H_2O for FBs, 1:5 with 70% MeOH for ZEA. No dilution for AFs, OTA, T-2	ELISA<br>AgraQuant® ELISA kits	2022	[30]
Pig and poultry feed	AFs, ZEA, Trichothecenes type B, FBs, OTA, T-2	0.5-10	SPE<br>25 g + 100 mL ACN/ H_2O (50/50) + blend 1 h + filtration. 2 mL + 0.1 mL acetic acid. 750 µL to SPE + eluate and centifuge 10000 rpm 1 min	LC-MS/MS<br>Column: Gemini C18 (150 × 4.6 mm, 5 µm) at 40ºC<br>Flow: 1 mL/min<br>Mobile phase: (A) H_2O /acetic acid (199/1); (B) MeOH /acetic acid (199/1), both containing 2 mM ammonium acetate, in gradient conditions<br>Detector: QTRAP, ESI (±), SRM	2022	[31]
Animal feed	AFB1, OTA, ZEA, DON, FB1, T-2	0.5-5	IAC<br>5 g + 1 g NaCl + 20 mL ACN/ H_2O (60:40) + vortex 30 min + centrifugation 6010 g 10 min at 4ºC<br>2 mL + 48 mL PBS 1% tween 20. Pass through a multi-IAC column prepared in the laboratory.<br>Eluent evaporated and reconstituted in MeOH/ H_2O (50:50)	LC-MS/MS<br>Column: UPLC® BEH C18 (100 × 2.1 mm, 1.7 µm) at 50ºC<br>Flow: 0.3 mL/min<br>Mobile phase: (A) MeOH 0.05% formic acid, (B) H_2O 0.15% formic acid containing 5 mM ammonium formate, in gradient conditions<br>Detector: QqQ, ESI (+), MRM	2022	[32]
Animal feed	AFB1, AFB2, AFG1 AFG2, OTA, ZEA, DON, FB1, FB2, T-2, HT-2	0.2-125	IAC<br>5 g + 20 mL (ACN/ H_2O /acetic acid) (79:20:1) + shake 30 min + centrifuge 4000 rpm 10 min<br>3 mL + 147 mL PBS.<br>20 mL passed through 11+MycoMSPREP IAC	LC-MS/MS<br>Column: Gemini C-18 (150 x 3 mm, 5 µm) at 40ºC<br>Flow: 0.3 mL/min<br>Mobile phase: (A) 1 mM ammonium formate and H_2O / MeOH /formic acid (94.9:5:0.1), (B) 1 mM ammonium formate and H_2O / MeOH /formic acid (1.9:98:0.1) in gradient conditions<br>Detector: QqQ, ESI (+), MRM	2022	[33]
Animal feed	AFB1, ZEA	0.25-1.25	SLE<br>5 g + 15 mL 70% MeOH/PBS (7:3) + vortex 5 min + centifuge 1600g 5 min.<br>Dilute supernatant to 50 mL with 0.5% tween 20/PBS (0.5%)	Lateral Flow Immunochromatographic Assay	2022	[34]

Table S1. Analytical methods employed for mycotoxin analysis in feed (continuation)

Matrix	Analyte/s	LOQ (μg/kg)	Sample preparation	Separation and detection technique	year	Ref
Cattle, pig, poultry and sheep feed	AFB1, AFB2, AFG1, AFG2, DON, OTA, STER, ZEA	0.8-84	SPE<br>0.5 g + 5 mL ACN/ H_2O / ortophosphoric acid (79/20/1) + vortex 1 h + centrifugation 5500 rpm 10 min. 2 mL pass through SPE Oasis prime HLB. 0.5 mL + 0.5 mL chloroform + vortex 10 s + 5 mL H_2O + vortex 30 s. 0.2 mL chloroform phase evaporated and redissolved in 0.2 mL mobile phase	LC-FLD<br>Column: C18 Cortecs T3 (150 × 4.6 mm, 2.7 μm) at 40ºC<br>Flow: 1.4 mL/min<br>Mobile phase: (A) H_2O (B) ACN (C) MeOH, all acidified with 0.1% ortophosphoric acid, in gradient conditions.<br>Detector: 365/440 nm (λex/λem) AFs, 234/469 nm (λex/λem) OTA, OTB, ZEA	2023	[35]
Pig and poultry feed	AFs, Trichothecenes type B, FBs, ZEA	0.5-10	SPE<br>25 g + 100 mL ACN/ H_2O (50/50) + blend 1 h + filtration. 2 mL + 0.1 mL acetic acid. 750 μL to SPE. Centifuge 10000 rpm 1 min	LC-MS/MS<br>Column: Gemini C18 (150 × 4.6 mm, 5 μm) at 40ºC<br>Flow: 1 mL/min<br>Mobile phase: (A) H_2O /acetic acid (199/1); (B) MeOH /acetic acid (199/1) both containing 2 mM ammonium acetate, in gradient conditions<br>Detector: QTRAP, ESI (±), SRM	2023	[36]

Table S2: Levels of mycotoxins in feed obtained from the bibliographic search (2019-2023)

Analyte	Matrix	n	% Positive sample (>LOQ)	Mean (µg/kg)	Maximum (µg/kg)	Origen of the samples	Collection of samples	Ref.
Total AFs	Pig feed	823	58.0	1.6	>20	Taiwan	2015-2017	[1]
DON			91.4	615.5	>5000			
ZEA			70.2	46.0	>1000			
Total FBs			50.4	1100	>5000			
Total AFs	Animal Feed	148	1.0	4.0	4.0	China	2021	[29]
	Poultry feed	434	32	14	206.0			
	Pig feed	350	21.0	4.0	49.0			
ZEA	Pig feed	350	99	88	857	China	2021	[29]
	Poultry feed	434	94	164	1490			
	Animal feed	148	91	87	499			
Total FBs	Pig feed	350	99	966	8539	China	2021	[29]
	Poultry feed	434	99	1263	12776			
	Animal feed	148	91	553	4618			
Total AFs	Animal Feed	782	60.2	6.22	66.66	Brazil	2017-2021	[30]
DON		741	67.8	690.0	4969.06			
ZEA		779	71.0	43.40	2503.86			
OTA		680	34.3	6.34	87.82			
Total FBs		792	45.4	970.0	17490.0			
T-2		681	24.7	24.11	135.23			
Total AFs	Animal Feed	85	1.0	3.0	3.0	China	2020	[31]
	Poultry feed	270	28	7	51.0			
	Pig feed	124	22.0	9.0	85.0			

Table S2: Raw data obtained from the bibliographic search (2019-2023) (Continuation)

Analyte	Matrix	n	% Positive sample (>LOQ)	Mean (μg/kg)	Maximum (μg/kg)	Origen of the samples	Collection of samples	Ref.
ZEA	Pig feed	124	51	73	331	China	2020	[31]
	Poultry feed	270	56	123	1094			
	Animal feed	85	68	111	719			
OTA	Pig feed	124	5	4	5.4	China	2020	[31]
	Poultry feed	270	5	50	27			
	Animal feed	85	n.i.	n.i.	n.i.			
Total FBs	Pig feed	124	95	871	4511	China	2020	[31]
	Poultry feed	270	91	990	4136			
	Animal feed	85	88	370	1287			
T-2	Pig feed	124	1	33	33	China	2020	[31]
	Poultry feed	270	n.i.	n.i.	n.i.			
	Animal feed	85	n.i.	n.i.	n.i.			
Total AFs	Animal Feed	470	2.8	8.5	39.0	China	2017-2021	[36]
	Poultry feed	1857	30.0	15.8	206.0			
	Pig feed	1418	21.9	16.9	245.0			
ZEA	Pig feed	1418	57.12	83.39	857	China	2017-2021	[36]
	Poultry feed	1857	71.46	120.13	1490			
	Animal feed	470	71.91	89.36	719			
Total FBs	Pig feed	1418	92.95	1116.0	13254	China	2017-2021	[36]
	Poultry feed	1857	95.85	1437.82	17052			
	Animal feed	470	93.36	554.15	7619			

Table S2: Raw data obtained from the bibliographic search (2019-2023) (Continuation)

Analyte	Matrix	n	% Positive sample (>LOQ)	Mean (µg/kg)	Maximum (µg/kg)	Origen of the samples	Collection of samples	Ref.
AFB1	Animal feed	45	13	n.i.	390	Brazil	2016	[3]
AFB2			4	n.i.	5.4			
AFG1			4	n.i.	12			
AFG2			0	n.i.	n.i.			
DON	Animal feed	45	44	n.i.	2300	Brazil	2016	[3]
ZEA			29	n.i.	520			
OTA			2	n.i.	11			
FB1	Animal feed	45	93	n.i.	53000	Brazil	2016	[3]
FB2			87	n.i.	2800			
AFB1	Poultry feed	105	98	0.2	0.9	South Africa	2015	[4]
AFB2			100	0.4	7.1			
AFG1			97	0.7	5.2			
AFG2			82	0.5	1.6			
DON	Poultry feed	105	98	37.8	154.0	South Africa	2015	[4]
3-ADON			95	1.6	12.9			
15-ADON			35	8.9	44.9			
ZEA	Poultry feed	105	99	71.2	428.9	South Africa	2015	[4]
α-ZEL				5.4	19.9			
β-ZEL				3.8	22.1			
OTA	Poultry feed	105	0	n.i.	n.i.	South Africa	2015	[4]
FB1	Poultry feed	105	100	1075.6	7125.3	South Africa	2015	[4]
FB2				28.5	125.1			
FB3				22.2	115.1			

Table S2: Raw data obtained from the bibliographic search (2019-2023) (Continuation)

Analyte	Matrix	n	% Positive sample (>LOQ)	Mean (µg/kg)	Maximum (µg/kg)	Origen of the samples	Collection of samples	Ref.
T-2	Poultry feed	105	100	3.1	15.3	South Africa	2015	[4]
HT-2				1.9	5.9			
AME	Poultry feed	105	100	23.1	155.5	South Africa	2015	[4]
AFB1	Poultry feed	43	n.i.	n.i.	n.i.	Tunisia	2016-2017	[5]
	Cattle feed	35	n.i.	n.i.	n.i.			
	Sheep feed	16	n.i.	n.i.	n.i.			
AFB2	Poultry feed	43	n.i.	n.i.	n.i.	Tunisia	2016-2017	[5]
	Cattle feed	35	n.i.	n.i.	n.i.			
	Sheep feed	16	n.i.	n.i.	n.i.			
AFG1	Poultry feed	43	n.i.	n.i.	n.i.	Tunisia	2016-2017	[5]
	Cattle feed	35	n.i.	n.i.	n.i.			
	Sheep feed	16	n.i.	n.i.	n.i.			
AFG2	Poultry feed	43	2	42.5	42.5	Tunisia	2016-2017	[5]
	Cattle feed	35	n.i.	n.i.	n.i.			
	Sheep feed	16	n.i.	n.i.	n.i.			
DON	Poultry feed	43	100	249.8	249.8	Tunisia	2016-2017	[5]
	Cattle feed	35	74	26.6	146.1			
	Sheep feed	16	6	105.1	25.3			
3-ADON	Poultry feed	43	21	158	167.9	Tunisia	2016-2017	[5]
	Cattle feed	35	6	29.8	168.7			
	Sheep feed	16	n.i.	n.i.	n.i.			

Table S2: Raw data obtained from the bibliographic search (2019-2023) (Continuation)

Analyte	Matrix	n	% Positive sample (>LOQ)	Mean (µg/kg)	Maximum (µg/kg)	Origen of the samples	Collection of samples	Ref.
15-ADON	Poultry feed	43	5	836.9	840.7	Tunisia	2016-2017	[5]
	Cattle feed	35	17	28.4	132.4			
	Sheep feed	16	25	18.7	19.0			
ZEA	Poultry feed	43	n.i.	n.i.	n.i.	Tunisia	2016-2017	[5]
	Cattle feed	35	3	35.1	35.1			
	Sheep feed	16	n.i.	n.i.	n.i.			
OTA	Poultry feed	43	n.i.	n.i.	n.i.	Tunisia	2016-2017	[5]
	Cattle feed	35	n.i.	n.i.	n.i.			
	Sheep feed	16	31	3.9	5.9			
T-2	Poultry feed	43	5	935.1	956.8	Tunisia	2016-2017	[5]
	Cattle feed	35	9	18.3	26.5			
	Sheep feed	16	n.i.	n.i.	n.i.			
HT-2	Poultry feed	43	9	119.8	119.8	Tunisia	2016-2017	[5]
	Cattle feed	35	37	21.0	173.4			
	Sheep feed	16	13	16.6	13.1			
NIV	Poultry feed	43	n.i.	n.i.	n.i.	Tunisia	2016-2017	[5]
	Cattle feed	35	n.i.	n.i.	n.i.			
	Sheep feed	16	n.i.	n.i.	n.i.			
AME	Poultry feed	43	2	109.7	109.7	Tunisia	2016-2017	[5]
	Cattle feed	35	n.i.	n.i.	n.i.			
	Sheep feed	16	n.i.	n.i.	n.i.			
AOH	Poultry feed	43	2	324.3	324.3	Tunisia	2016-2017	[5]
	Cattle feed	35	n.i.	n.i.	n.i.			
	Sheep feed	16	n.i.	n.i.	n.i.			

Table S2: Raw data obtained from the bibliographic search (2019-2023) (Continuation)

Analyte	Matrix	n	% Positive sample (>LOQ)	Mean (µg/kg)	Maximum (µg/kg)	Origen of the samples	Collection of samples	Ref.
NEO	Poultry feed	43	n.i.	n.i.	n.i.	Tunisia	2016-2017	[5]
	Cattle feed	35	n.i.	n.i.	n.i.			
	Sheep feed	16	n.i.	n.i.	n.i.			
DAS	Poultry feed	43	14	118.8	219.2	Tunisia	2016-2017	[5]
	Cattle feed	35	n.i.	n.i.	n.i.			
	Sheep feed	16	n.i.	n.i.	n.i.			
TENT	Poultry feed	43	n.i.	n.i.	n.i.	Tunisia	2016-2017	[5]
	Cattle feed	35	6	2.8	2.9			
	Sheep feed	16	6	7.0	7.0			
BEA	Poultry feed	43	100	7.1	29.3	Tunisia	2016-2017	[5]
	Cattle feed	35	69	3.1	5.7			
	Sheep feed	16	n.i.	n.i.	n.i.			
ENNA	Poultry feed	43	37	4.0	12.6	Tunisia	2016-2017	[5]
	Cattle feed	35	20	0.7	0.9			
	Sheep feed	16	25	2.9	4.3			
ENNA1	Poultry feed	43	51	1.5	5.0	Tunisia	2016-2017	[5]
	Cattle feed	35	77	1.8	4.3			
	Sheep feed	16	25	10.9	20.5			
ENNB	Poultry feed	43	79	6.7	39.8	Tunisia	2016-2017	[5]
	Cattle feed	35	80	9.0	21.9			
	Sheep feed	16	69	12.5	21.7			
ENNB1	Poultry feed	43	49	3.5	15.7	Tunisia	2016-2017	[5]
	Cattle feed	35	n.i.	n.i.	n.i.			
	Sheep feed	16	38	0.8	2.8	7.2	12.8	

Table S2: Raw data obtained from the bibliographic search (2019-2023) (Continuation)

Analyte	Matrix	n	% Positive sample (>LOQ)	Mean (µg/kg)	Maximum (µg/kg)	Origen of the samples	Collection of samples	Ref.
AFB1	Cattle feed	17	47	1.5	5.9	Egypt	2014-2015	[6]
AFB2		17	6	0.5	0.5			
AFG1		17	n.i.	n.i.	n.i.			
AFG2		17	n.i.	n.i.	n.i.			
ZEA	Cattle feed	17	24	8.1	11.9	Egypt	2014-2015	[6]
OTA			n.i.	n.i.	n.i.			
AFB1	Pig feed	228	3.07	0.94	2.91	Spain	2017	[7]
AFB2			1.32	0.60	1.06			
AFG1			0.88	0.33	0.44			
AFG2			0	-	-			
DON	Pig feed	228	4.39	237	555	Spain	2017	[7]
ZEA			7.02	741	7681			
OTA			0	-	-			
FB1	Pig feed	228	50	403	3959	Spain	2017	[7]
FB2			29.82	184	961			
T-2	Pig feed	228	0.88	31.9	35.9	Spain	2017	[7]
HT-2			0.88	117	123			
BEA	Pig feed	228	93.42	20.7	747	Spain	2017	[7]
ENNA			5.26	9.82	64.9			
ENNA1			40.79	19.0	140			
ENNB			100	118	1222			
ENNB1			53.51	34.3	247			

Table S2: Raw data obtained from the bibliographic search (2019-2023) (Continuation)

Analyte	Matrix	n	% Positive sample (>LOQ)	Mean (μg/kg)	Maximum (μg/kg)	Origen of the samples	Collection of samples	Ref.
CIT	Pig feed	228	14.04	147	512	Spain	2017	[7]
FUS-X			5.70	291	821			
STER			2.19	104	308			
AFB1	Poultry feed	30	83.3	74	760	Nigeria	2013	[8]
AFB2			50.0	21	188			
AFG1			56.7	19	79			
AFG2			13.3	3.5	7.6			
ZEA	Poultry feed	30	83.3	9.3	71	Nigeria	2013	[8]
ZEA-sulfate			13.3	56	162			
OTA	Poultry feed	30	26.7	5.4	15	Nigeria	2013	[8]
OTB			20.0	9.3	24			
FB1	Poultry feed	30	96.7	1014	3760	Nigeria	2013	[8]
FB2			93.3	310	870			
FB3			90.0	62	149			
FB4			96.7	623	168			
NIV	Poultry feed	30	23.3	114	647	Nigeria	2013	[8]
AOH			40.0	2.7	8.6			
TENT			70.0	44	315			
BEA			100	13	127			
CIT			16.7	522	2340			
Cyclopiazonic acid			10.0	39	49			
MON			93.3	62	900			

Table S2: Raw data obtained from the bibliographic search (2019-2023) (Continuation)

Analyte	Matrix	n	% Positive sample (>LOQ)	Mean (µg/kg)	Maximum (µg/kg)	Origen of the samples	Collection of samples	Ref.
Total AFs	Animal feed	365	n.i.	88.6	n.i.	Rwanda	2017	[2]
Total FBs			n.i.	1480	n.i.			
Total AFs	Cattle feed	1180	n.i.	108.8	n.i.	Rwanda	2017	[2]
Total FBs			n.i.	1520	n.i.			
Total AFs	Poultry feed	1726	n.i.	103.8	n.i.	Rwanda	2017	[2]
Total FBs			n.i.	1210	n.i.			
AFB1	Pig feed	100	34	1.7	14.2	Thailand	-	[13]
	Poultry feed	100	77	8.2	326.4			
	Cattle feed	100	32	1.6	14.9			
AFB2	Pig feed	100	13	0.9	4.1	Thailand	-	[13]
	Poultry feed	100	35	2.4	49.9			
	Cattle feed	100	8	0.9	2.4			
AFG1	Pig feed	100	4	0.5	1.6	Thailand	-	[13]
	Poultry feed	100	25	0.6	0.86			
	Cattle feed	100	12	0.5	1.6			
AFG2	Pig feed	100	1	0.66	0.66	Thailand	-	[13]
	Poultry feed	100	n.i.	n.i.	n.i.			
	Cattle feed	100	n.i.	n.i.	n.i.			
DON	Pig feed	100	43	215	631.9	Thailand	-	[13]
	Poultry feed	100	31	304.6	1430.8			
	Cattle feed	100	37	167.8	538.8			

Table S2: Raw data obtained from the bibliographic search (2019-2023) (Continuation)

Analyte	Matrix	n	% Positive sample (>LOQ)	Mean (µg/kg)	Maximum (µg/kg)	Origen of the samples	Collection of samples	Ref.
3-ADON	Pig feed	100	n.i.	n.i.	n.i.	Thailand	-	[13]
	Poultry feed	100	1	45.6	45.6			
	Cattle feed	100	3	36.0	46.6			
15-ADON	Pig feed	100	16	30.8	83.2	Thailand	-	[13]
	Poultry feed	100	26	31.7	57.1			
	Cattle feed	100	36	35.9	68.2			
ZEA	Pig feed	100	91	17.4	169.2	Thailand	-	[13]
	Poultry feed	100	72	30.2	235.8			
	Cattle feed	100	46	26.1	98.4			
OTA	Pig feed	100	n.i.	n.i.	n.i.	Thailand	-	[13]
	Poultry feed	100	1	3.1	3.1			
	Cattle feed	100	n.i.	n.i.	n.i.			
FB1	Pig feed	100	85	102.4	464.8	Thailand	-	[13]
	Poultry feed	100	96	451.7	2645.5			
	Cattle feed	100	62	88.1	731.0			
FB2	Pig feed	100	77	31.08	136.1	Thailand	-	[13]
	Poultry feed	100	91	123.2	573.3			
	Cattle feed	100	45	26.1	252.2			
T-2	Pig feed	100	n.i.	n.i.	n.i.	Thailand	-	[13]
	Poultry feed	100	4	2.6	3.2			
	Cattle feed	100	2	4.5	5.5			
HT-2	Pig feed	100	7	9.6	19.3	Thailand	-	[13]
	Poultry feed	100	7	6.6	10.0			
	Cattle feed	100	1	15.23	15.23			

Table S2: Raw data obtained from the bibliographic search (2019-2023) (Continuation)

Analyte	Matrix	n	% Positive sample (>LOQ)	Mean (µg/kg)	Maximum (µg/kg)	Origen of the samples	Collection of samples	Ref.
NIV	Pig feed	100	18	46.0	165.4	Thailand	-	[13]
	Poultry feed	100	32	103.4	626.0			
	Cattle feed	100	6	51.2	117.5			
NEO	Pig feed	100	n.i.	n.i.	n.i.	Thailand	-	[13]
	Poultry feed	100	n.i.	n.i.	n.i.			
	Cattle feed	100	n.i.	n.i.	n.i.			
DAS	Pig feed	100	2	4.7	5.1	Thailand	-	[13]
	Poultry feed	100	3	3.1	3.6			
	Cattle feed	100	1	4.36	4.36			
FUS-X	Pig feed	100	n.i.	n.i.	n.i.	Thailand	-	[13]
	Poultry feed	100	3	48.0	61.9			
	Cattle feed	100	n.i.	n.i.	n.i.			
AFB1	Cattle feed	193	12	n.i.	4.66	Spain	2016-2018	[15]
AFB2			5	n.i.	0.41			
AFG1			24	n.i.	6.45			
AFG2			7	n.i.	0.57			
AFB1	Animal feed	51	61	2.42	5.0	Lithuania	2019-2020	[18]
DON			55	283.94	500			
ZEA			49	377.60	700			
T-2			29	106.05	246.7			
AFB1	Animal feed	34	n.i.	n.i.	n.i.	Thailand	2018-2019	[20]
DON			26.5	61.84	122.44			
DON-3gluc			26.5	10.92	28.78			
ZEA			82.4	5.16	12.44			

Table S2: Raw data obtained from the bibliographic search (2019-2023) (Continuation)

Analyte	Matrix	n	% Positive sample (>LOQ)	Mean (µg/kg)	Maximum (µg/kg)	Origen of the samples	Collection of samples	Ref.
FB1	Animal feed	34	41.2	26.81	60.8	Thailand	2018-2019	[20]
FB2			14.7	12.52	18.28			
FB3			n.i.	n.i.	n.i.			
AFB1	Cattle feed	60	55	0.61	5.17	Spain	2015-2016	[21]
AFB1	Pig feed	620	100	4.1	59.7	China	2018-2020	[23]
	Poultry feed	571	99.9	4.9	57.4			
	Cattle feed	225	100	9.5	77.5			
DON	Pig feed	620	99.6	659.3	3712.2	China	2018-2020	[23]
	Poultry feed	571	99.7	660.8	2970.1			
	Cattle feed	225	99.3	752.1	2254.7			
ZEA	Pig feed	620	99.4	81.5	1599.0	China	2018-2020	[23]
	Poultry feed	571	100	108.0	852.8			
	Cattle feed	225	99.3	98.2	906.9			
AFB1	Cattle feed	77	3.9	26.1	30.2	South Africa	2018-2019	[24]
AFB2			3.9	4.4	6.8			
AFG1			2.6	20.2	23.1			
AFG2			1.3	11.1	11.1			
DON	Cattle feed	77	63.6	477.7	2385.4	South Africa	2018-2019	[24]
3-ADON			16.9	55.5	300.0			
15-ADON			20.8	169.6	858.8			
ZEA	Cattle feed	77	9.1	666.0	1793.7	South Africa	2018-2019	[24]
OTA			3.9	85.6	187.9			
FB1	Cattle feed	77	23.4	189.8	485.2	South Africa	2018-2019	[24]
FB2			19.5	132.4	416.9			
FB3			1.3	n.i.	n.i.			

Table S2: Raw data obtained from the bibliographic search (2019-2023) (Continuation)

Analyte	Matrix	n	% Positive sample (>LOQ)	Mean (µg/kg)	Maximum (µg/kg)	Origen of the samples	Collection of samples	Ref.
NIV	Cattle feed	77	5.2	36.9	36.9	South Africa	2018-2019	[24]
AME			6.5	229.2	603.2			
AOH			42.8	279.2	3088.2			
DAS			1.3	3.4	3.4			
ENNB			32.5	1143.1	14230.4			
STER			45.5	25.8	139.1			
ROQC			2.6	377.2	699.9			
AFB1	Cattle feed	100	12	3.4	5.4	Spain	2019-2020	[35]
	Pig feed	100	7	5.3	6.2			
	Poultry feed	100	13	5.0	6.9			
	Sheep feed	100	12	4.4	6.1			
AFB2	Cattle feed	100	11	1.5	3.2	Spain	2019-2020	[35]
	Pig feed	100	14	2.1	3.9			
	Poultry feed	100	11	1.7	3.1			
	Sheep feed	100	15	2.0	4.9			
AFG1	Cattle feed	100	7	2.9	3.4	Spain	2019-2020	[35]
	Pig feed	100	10	4.1	6			
	Poultry feed	100	7	4.3	5.6			
	Sheep feed	100	10	4.3	6.5			
AFG2	Cattle feed	100	9	1.7	3.3	Spain	2019-2020	[35]
	Pig feed	100	17	1.9	4.4			
	Poultry feed	100	14	2.0	3.9			
	Sheep feed	100	16	1.9	4			

Table S2: Raw data obtained from the bibliographic search (2019-2023) (Continuation)

Analyte	Matrix	n	% Positive sample (>LOQ)	Mean (µg/kg)	Maximum (µg/kg)	Origen of the samples	Collection of samples	Ref.
DON	Cattle feed	100	76	177.8	574	Spain	2019-2020	[35]
	Pig feed	100	72	157.5	410			
	Poultry feed	100	71	255.3	755			
	Sheep feed	100	72	238.3	887			
ZEA	Cattle feed	100	49	133.4	413	Spain	2019-2020	[35]
	Pig feed	100	50	162.3	816			
	Poultry feed	100	66	150.0	489			
	Sheep feed	100	52	201.3	658			
OTA	Cattle feed	100	6	6.3	7.7	Spain	2019-2020	[35]
	Pig feed	100	7	22.9	65.5			
	Poultry feed	100	5	18.1	23.2			
	Sheep feed	100	8	21.3	45.3			
STER	Cattle feed	100	6	3.3	4.7	Spain	2019-2020	[35]
	Pig feed	100	10	3.6	6.1			
	Poultry feed	100	7	3.4	5.1			
	Sheep feed	100	5	3.7	5.6			
Total AFs	Cattle feed	88	n.i.	4.2	5.1	Jordan	2018	[12]
DON			n.i.	49.5	2490			
ZEA			n.i.	80.2	333.5			
Total FBs			n.i.	5537	11638.2			
T-2			n.i.	266	1734.6			
Total AFs	Poultry feed	27	93.0.	17.2	89.0	Kenya	2019	[14]
AFB1	Cattle feed	16	94	31.2	134	Kenya	2019	[14]
	Poultry feed	27	93	10.2	38.8			
AFB2	Cattle feed	16	81	5.1	22.1	Kenya	2019	[14]
	Poultry feed	27	48	1.7	4.4			

Table S2: Raw data obtained from the bibliographic search (2019-2023) (Continuation)

Analyte	Matrix	n	% Positive sample (>LOQ)	Mean (µg/kg)	Maximum (µg/kg)	Origen of the samples	Collection of samples	Ref.
AFG1	Cattle feed	16	88	21.7	123	Kenya	2019	[14]
	Poultry feed	27	70	6.7	41.7			
AFG2	Cattle feed	16	44	8.8	28.5	Kenya	2019	[14]
	Poultry feed	27	33	2.5	6.4			
DON	Cattle feed	16	94	359.4	567	Kenya	2019	[14]
	Poultry feed	27	100	329.1	1037			
DON-3gluc	Cattle feed	16	88	22.1	61.7	Kenya	2019	[14]
	Poultry feed	27	100	16.4	45.7			
ZEA	Cattle feed	16	100	35.2	140.2	Kenya	2019	[14]
	Poultry feed	27	100	103.4	873.4			
OTA	Cattle feed	16	56	5.6	24.3	Kenya	2019	[14]
	Poultry feed	27	19	4.8	10.6			
Total FBs	Cattle feed	16	100	652.4	2171.3	Kenya	2019	[14]
	Poultry feed	27	100	597.9	2684.8			
FB1	Cattle feed	16	100	487.9	1494	Kenya	2019	[14]
	Poultry feed	27	100	431.4	1926			
FB2	Cattle feed	16	94	175.5	677.3	Kenya	2019	[14]
	Poultry feed	27	96	172.9	728.8			
FB3	Cattle feed	16	63	79.8	124.3	Kenya	2019	[14]
	Poultry feed	27	85	70.8	243			
FB4	Cattle feed	16	75	54.2	124.8	Kenya	2019	[14]
	Poultry feed	27	89	73.7	387.8			
FA1	Cattle feed	16	38	39	83.2	Kenya	2019	[14]
	Poultry feed	27	52	14.2	29.2			
FA2	Cattle feed	16	75	31.9	87.2	Kenya	2019	[14]
	Poultry feed	27	74	24.5	103.1			

Table S2: Raw data obtained from the bibliographic search (2019-2023) (Continuation)

Analyte	Matrix	n	% Positive sample (>LOQ)	Mean (µg/kg)	Maximum (µg/kg)	Origen of the samples	Collection of samples	Ref.
T-2	Cattle feed	16	13	3.5	4.4	Kenya	2019	[14]
	Poultry feed	27	4	5.2	5.2			
HT-2	Cattle feed	16	6	11.9	11.9	Kenya	2019	[14]
	Poultry feed	27	4	13.8	13.8			
NIV	Cattle feed	16	94	51.1	102.1	Kenya	2019	[14]
	Poultry feed	27	96	43.2	105.5			
ERGOT	Cattle feed	16	63	56.9	285.7	Kenya	2019	[14]
	Poultry feed	27	81	26	113.2			
Total AFs	Cattle feed	189	59.0	40.2	406.1	India	2017-2018	[19]
AFB1			59	35.6	374.6			
AFB2			59	3.9	31.5			
AFG1			11	0.3	3.9			
AFG2			5	n.i.	n.i.			
Total AFs	Poultry feed	27	7.4	0.39	0.63	Malaysia	2017-2018	[22]
AFB1			n.i.	n.i.	n.i.			
AFB2			n.i.	n.i.	n.i.			
AFG1			n.i.	n.i.	n.i.			
AFG2			n.i.	n.i.	n.i.			
Total AFs	Poultry feed	40	97.5	48.6	86.2	Pakistan	2018	[26]
Total AFs	Poultry feed	33	94	190.2	1919.8	Ethiopia	2018-2019	[27]
AFB1			n.i.	70.11	633.94			
AFB2			n.i.	13.50	142.98			
AFG1			n.i.	88.55	921.43			
AFG2			n.i.	18.00	221.43			
Total AFs	Cattle feed	293	2.4	<5	>20	Italy	2013-2021	[28]

Table S3. Analytical methods employed for the analysis of mycotoxin biomarkers in animal biological fluids.

Animal	Matrix	Analyte/s	LOD (ng/g or ng/mL)	LOQ (ng/g or ng/mL)	Sample preparation	Separation and detection Technique	Year	Ref.
Pig	Plasma<br>Urine	OTA, OTα	1.5	5	**LLE**<br>Normalize direct urine with 10 µM creatinine.<br>200 µL urine or plasma + 8 µL (1µg/mL ^{13}C-OTA and ^{13}C-OTα) + 800 µL EtOAc/phosphoric acid 85% (99/1, v/v). Centrifuge + 50 µL supernatant + 50 µL ACN	**LC-MS/MS**<br>Column: C18 (150 mm x 2.1 mm x 2.6µm) at 40ºC<br>Flow: 0.4 mL/min<br>MP: (A) H_2O/ACN (95/5 v/v) (0.1 FA); (B) ACN/H_2O (95/5, v/v) (0.1% FA) in gradient conditions<br>Detector: ESI (-), QTrap, MRM	2023	[37]
	Feces	OTA, OTα	3.0	10	1 g + 30 µL ^{13}C-OTA and ^{13}C-OTα (1µg/mL) + 6 mL phosphoric acid, 1M + 30 mL EtOAc. Extract 60 min at 80 rpm. Centrifuge. 3 mL water phase + 70 µL orthophosphoric acid 85% + 1.5 mL EtOAc. Mix at 80 rpm. Centrifuge. 50 µL supernatant + 50 µL ACN			
Pig, poultry, cattle, sheep	Plasma	AFB1, OTA, ZEN, DON, 3/15-ADON, DOM-1, T-1, HT-2, AFM1, STER, NEO, DAS, FUS-X, AFB2, AFG1, AFG2, OTB, NIV	0.04-9.10	0.2-20.4	**SPE Captiva**<br>400 µL plasma + SEP Captiva + 1.2 mL ACN (1% FA). Elute and evaporate. Group I: 400 µL, evaporate + 200 µL MF: 60A/40B% (A: 5mM ammonium formate, 0.1% FA in water; B: 5mM ammonium formate, 0.1% FA in a 95.5 MeOH/H_2O). Group II: 400 µL + evaporate + 200 MF: 95A/5B%.<br>450 µL plasma + 50µL β-glucuronidase-arylsulfatase + above described SPE Captiva extraction	**LC-MS/MS**<br>Column: C18 (150 mm x 2.1 mm x 2.7µm) at 45ºC<br>Flow: 0.4 mL/min<br>MP: (A) 5 mM ammonium formate, 0.1% FA in H_2O; (B) 5 mM ammonium formate, 0.1% FA in a 95.5 MeOH/H_2O, v/v) in gradient conditions<br>Detector: ESI (+), QqQ, MRM	2023	[38]
Poultry and cattle	Plasma	AFB1, AFB2, AFG1, AFG2	0.003-0.03	0.05-0.1	**SPE**<br>100 µL plasma + 25 µL IS + 100 µL ACN + 300 µL 1% FA in ACN + centrifuge + Oasis® Ostro-96-well plate. Evaporate (N_2) and reconstitute in 200 µL of H_2O/MeOH (50/50, v/v)	**LC-MS/MS**<br>Column: C18 (100 mm x 2.1 mm x 1.8 µm) at 40ªC<br>Flow: 0.3 mL/min<br>MP: (A) H_2O; (B) MeOH, in gradient conditions<br>Detector: ESI (+), QqQ, MRM	2023	[39]

Table S3. Analytical methods employed for the analysis of mycotoxin biomarkers in animal biological fluids (continuation)

Animal	Matrix	Analyte/s	LOD (ng/g or ng/mL)	LOQ (ng/g or ng/mL)	Sample preparation	Separation and detection Technique	Year	Ref.
Pig	Serum<br>Urine	DON, DOM-1, iso-DON, 3/15-ADON, 3/15-ADOM, isoDOM	n.i.	n.i.	**LLE**<br>50 µL serum + 35 µL β-glucuronidase-arylsulfatase (18 h. 37°) + 300 µL MeOH/AA (99.8/0.2, v/v)<br>Urine samples: Direct injection	**LC-MS/MS**<br>Column: C18 (150 mm x 2.1 mm x 2.6 µm) at 30ªC<br>Flow: 0.25 mL/min<br>MP: (A) H_2O/AA (99.9/0.1, v/v); (B) ACN/AA (99.9/0.1, v/v), in gradient conditions<br>Detector: ESI (-), QTrap, SRM	2023	[40]
Pig and poultry	Feces/ excreta<br><br>Liver, Kidney, Muscle, Skin and Fat<br><br>Urine<br>Plasma	OTA, OTα<br><br>OTA, OTα<br><br>OTA, OTα	3.0<br><br>0.15<br><br>1.5	10<br><br>0.5<br><br>5	**LLE**<br>1 g + 30 µL ^{13}C-OTA and ^{13}C-OTα (1µg/mL) + 6 mL phosphoric acid, 1M + 30 mL EtOAc. Extract 60 min at 80 rpm. Centrifuge. 3 mL water phase + 70 µL orthophosphoric acid 85% + 1.5 mL EtOAc. Mix at 80 rpm. Centrifuge. 50 µL supernatant + 50 µL ACN<br><br>Dilute urine sample to 10 µM creatinine. 200 µL + 8 µL (1µg/mL ^{13}C-OTA and ^{13}C-OTα) + 800 µL EtOAc /phosphoric acid 85% (99/1, v/v). Centrifuge. Centrifuge. 50 µL supernatant + 50 µL ACN	**LC-MS/MS**<br>Column: C18 (150 mm x 2.1 mm x 2.6µm) at 40ºC<br>Flow: 0.4 mL/min<br>MP: (A) H_2O/ACN (95/5 v/v) (0.1 FA); (B) ACN/H_2O (95/5 v/v) (0.1 FA) in gradient conditions<br>Detector: ESI (-), QTrap, MRM	2022	[41]
Poultry	Excreta	FB1, FB2, FB3<br>HFB1, HFB2, HFB3	50	160	**LLE and SPE (two methods)**<br>5 g + 20 mL ACN/H_2O/FA (74:25:1). Shake, ultrasonicate and centrifuge. 50 µL supernatant + 950 µL destiled water. A) 50 µL + 10 µL 13-C-FBs IS solution + 850 µL 0.2% FA in MeOH/H_2O (1:9)<br>B) hydrolysis: 50 µL + 10 µL 13-C-FBs IS solution + 850 µL 2.5M NaOH (2 h. at 70ºC) + Oasis MAX. Wash (3mL of 2% ammonium hydroxide) and eluted (3 mL 2% FA in MeOH). Evaporate (N_2) and reconstitute with 0.2% FA in MeOH/H_2O (1:9)	**LC-MS/MS**<br>Column: C18 (100 mm x 2.1 mm x 1.6 µm) at 40ªC<br>Flow: 0.4 mL/min<br>MP: (A) H_2O; (B) MeOH both with 0.2% FA, in gradient conditions<br>Detector: ESI (+), QqQ, MRM	2022	[42]

Table S3. Analytical methods employed for the analysis of mycotoxin biomarkers in animal biological fluids (continuation)

Animal	Matrix	Analyte/s	LOD (ng/g or ng/mL)	LOQ (ng/g or ng/mL)	Sample preparation	Separation and detection Technique	Year	Ref.
Pig	Urine	DON, 3-ADON, 15-ADON, DOM-1, ZEA,α-ZEL,β-ZEL, α-ZAL, β-ZAL, ZAN, OTA, AFB1, AFB2, AFG1, AFM1, T-2, HT-2, NIV, TEN, AOH, AME, ATX-I, CIT, DAS, FUS-X, STER, T-2 triol, OTα, HFB1, DH-CIT, ENNs and BEA	0.03-2	0.1-8	**LLE**<br>Normalize direct urine with creatinine.<br>(A) 250 µL urine + 1 g NaCl + 500 µL EtOAc. Centrifuge. Evaporate (N_2) and reconstitute with 50% FM A and B<br>(B) 250 µL urine + β-glucuronidase (2 h, 40°C)	**LC-MS/MS**<br>Column: C18 (150 mm x 2 mm x 3 µm) at 35°C<br>Flow: 0.45 mL/min<br>MP: (A) 95% MeOH/5% 10 mM ammonium acetate, 0.001% AA; (B) 5% MeOH/95% 10 mM ammonium acetate, 0.001% AA in gradient conditions<br>Detector: ESI (±), QTrap, MRM	2021	[43]
Pig	Plasma	DON, DOM1, ZEA, α-ZEL, OTA, OTα, CIT, DH-CIT	n.i.	n.i.	**LLE**<br>250 µL serum + 750 µL ACN (0.1% FA).Centrifuge. 800 µL supernatant + 10 µL IS. Evaporate (N_2) and reconstitute with 100 µL FM (50% eluent A and B)	**LC-MS/MS**<br>Column: C18 (150 mm x 2 mm x 3 µm) at 35°C<br>Flow: 0.4 mL/min<br>MP: (A) H_2O/5 mM ammonium acetate, 0.05% AA; (B) MeOH/5 mM ammonium acetate, 0.05% AA in gradient conditions<br>Detector: ESI (-), QqQ, MRM	2021	[44]
	Urine	DON, DOM1, ZEA, α-ZEL, OTA, OTα, CIT, DH-CIT	0.03-2	0.1-8	Normalize direct urine with creatinine.<br>250 µL urine + 1 g NaCl + 500 µL EtOAc. Centrifuge. Evaporate (N_2) and reconstitute with 50% FM A and B			
Pig and poultry	Plasma	CIT<br>DH-CIT	0.05<br>0.01	0.1<br>0.1	**LLE and SPE**<br>(A) 250 µL pig plasma + 750 µL ACN. Centrifuge. Evaporate supernatant (N_2) and reconstitute with 250 µL H_2O/MeOH (50/50, v/v)<br>(B) 100 µL poultry plasma + Oasis® Ostro 96-well plate + 300 µL ACN/FA (99.9/0.1, v/v). Evaporate (N_2) and reconstitute with 100 µL H_2O/MeOH; 50/50, v/v	**LC-MS/MS**<br>Column: C18 (100 mm x 2.1 mm x 1.8 µm) at 40ªC<br>Flow: 0.4 mL/min<br>MP: (A) H_2O ; (B) MeOH both with 0.2% FA, in gradient conditions<br>Detector: ESI (+), QqQ, MRM	2020	[45,46]

Table S3. Analytical methods employed for the analysis of mycotoxin biomarkers in animal biological fluids (continuation)

Animal	Matrix	Analyte/s	LOD (ng/g or ng/mL)	LOQ (ng/g or ng/mL)	Sample preparation	Separation and detection Technique	Year	Ref.
Poultry	Plasma	FB1<br>HFB1	0.15<br>0.17	1<br>2.5	**SPE**<br>100 µL plasma + Oasis® Ostro 96-well plate + 300 µL ACN/1% FA	**LC-MS/MS**<br>Column: C18 (100 mm x 2.1 mm x 1.8 µm) at 40ªC<br>Flow: 0.4 mL/min<br>MP: (A) H_2O/10 mM ammonium formate, 0.3% FA; (B) ACN, in gradient conditions<br>Detector: ESI (+), QqQ, MRM M	2020	[47]
Pig	Plasma<br>Feces<br>Urine	ZEN, α-ZEL, β-ZEL, α-ZAL, β-ZAL, ZAN, TEA, AOH, AME, DON, DOM-1, 3/15 ADON, T2, HT2, T2G, AFB1, AFM1, OTA, ENNA1, ENNA, ENNB, ENNB1, BEA	n.i	1.0-5.0	**LLE and SPE**<br>(A) Plasma: 250 µL + 750 µL ACN. Centrifuge. Evaporate supernatant (N_2) and reconstitute with 250 µL MeOH/H_2O (85/15, v/v)<br>(B) Feces: a) 250 mg + 5 mL MeOH/EtOAc/FA (75:24:1), shake, centrifuge (OTA, TeA, AME and AOH); b) 250 mg + 5 mL Acetone, shake, centrifuge + HybridSPE-phosfolipid cartridge (other mycotoxins)<br>Urine: 500 µL (at pH 2 and 8) + 300 µL EtOAc. Centrifuge. Evaporate (N2) and reconstitute with 250 µL MeOH/H_2O (85/15, v/v)	**LC-MS/MS**<br>Column: C18 (100 mm x 2.1 mm x 1.8 µm) at 45ºC<br>Flow: 0.3 mL/min<br>MP: (A) H_2O ; (B) MeOH both with 10 mM ammonium formate and 0.3% FA, in gradient conditions<br>Detector: ESI (+), QqQ, SRM	2019	[48]
Poultry	Plasma<br>Excreta	ZEN, α-ZEL, β-ZEL, α-ZAL, β-ZAL, ZAN, TEA, AOH, AME, DON, DOM-1, 3/15 ADON, T2, HT2, T2G, AFB1, AFM1, OTA, ENNA1, ENNA, ENNB, ENNB1, BEA	n.i.	1.0-10	**LLE and SPE**<br>(A) Plasma: 150 µL + Oasis® Ostro 96-well plate + 450 µL ACN/1%. Evaporate (N_2). Reconstitute with 150 µL MeOH/H_2O (85/15, v/v)<br>(C) Excreta: 250 mg + 1.5 mL ACN, shake and centrifuge. Evaporate (N2) and reconstitute with 250 µL MeOH/H_2O (85/15, v/v)	**LC-MS/MS**<br>Column: C18 (100 mm x 2.1 mm x 1.8 µm) at 45ºC<br>Flow: 0.3 mL/min<br>MP: (A) H_2O ; (B) MeOH both with 10 mM ammonium formate and 0.3% FA, in gradient conditions<br>Detector: ESI (+), QqQ, SRM	2019	[48]

Table S3. Analytical methods employed for the analysis of mycotoxin biomarkers in animal biological fluids (continuation)

Animal	Matrix	Analyte/s	LOD (ng/g or ng/mL)	LOQ (ng/g or ng/mL)	Sample preparation	Separation and detection Technique	Year	Ref.
Pig and poultry	Plasma<br>Feces/excreta<br>Urine	AFB1, DON, DON-s, DON-GlcA, ZEA, ZEA-GlcA,	0.001-1.68	1.0-5.0	**LLE and SPE**<br>(A) Poultry plasma: 150 µL + Oasis® Ostro 96-well plate + 450 µL ACN/1%. Evaporate(N_2) and reconstitute with 150 µL MeOH/H_2O (85/15, v/v)<br>(B) Pig plasma: 250 µL + 750 µL ACN. Centrifuge. Evaporate supernatant (N_2) and reconstitute with 250 µL MeOH/H_2O (85/15, v/v)<br>(C) Feces: 250 mg + 20 µL IS. Centrifuge + HyBridSPE-phospholipid cartridge. Evaporate (N_2) and reconstitute with 250 µL MeOH/H_2O (85/15, v/v)<br>(B) Urine: 500 µL (at pH 2 and 8) + 300 µL EtOAc. Centrifuge. Evaporate (N_2) and reconstitute with 250 µL MeOH/H_2O (85/15, v/v)	**LC-MS/MS**<br>Column: C18 (100 mm x 2.1 mm x 1.8 µm) at 45°C<br>Flow: 0.3 mL/min<br>MP: (A) H_2O ; (B) MeOH both with 10 mM ammonium formate and 0.3% FA, in gradient conditions<br>Detector: ESI (+), QqQ, SRM	2019	[49]
Pig and poultry	Plasma<br>DBS	DON, DOM1, 3/15ADON, AFB1, AFM1, ENNA, ENNA1, ENNB, ENNB1, BEA, FB1, FB2, OTA, ZEA, α-ZEL, β-ZEL, α-ZAL, β-ZAL, ZAN, TEA, AOH, AME, T-2	0.001-0.74	0.5-10	**LLE and SPE**<br>(A) 60 µL blood + H_2O/ACN/acetone (30/35/35, v/v/v). Dried paper (N_2) and reconstitute with 60 µL H_2O/MeOH/FA (60/39.9/0.1, v/v/v).<br>(B) Poultry plasma: 150 µL + Oasis® Ostro 96-well plate + 450 µL ACN/1%. Evaporate(N_2) and reconstitute with 150 µL MeOH/H_2O (85/15, v/v)<br>(D) Pig plasma: 250 µL + 750 µL ACN. Centrifuge. Evaporate supernatant (N_2) and reconstitute with 250 µL MeOH/H_2O (85/15, v/v)	**LC-MS/MS**<br>Column: C18 (100 mm x 2.1 mm x 1.8 µm) at 45°C<br>Flow: 0.3 mL/min<br>MP: (A) H_2O ; (B) MeOH both with 10mM ammonium formate and 0.3% FA, in gradient conditions<br>Detector: ESI (+), QqQ, SRM	2019	[50]

Table S3. Analytical methods employed for the analysis of mycotoxin biomarkers in animal biological fluids (continuation)

Animal	Matrix	Analyte/s	LOD (ng/g or ng/mL)	LOQ (ng/g or ng/mL)	Sample preparation	Separation and detection Technique	Year	Ref.
Pig	Plasma	ZEA, α,β-ZEL, α,β-ZAL, ZAN, ZEN14G, ZEN14S	0.02-0.2	0.04-0.41	**LLE**<br>250 µL plasma + 1000 µL ACN. Centrifuge. Evaporate supernatant (N_2) and reconstitute with 250 µL H_2O/MeOH (85/15, v/v)<br>(C)	**LC-MS/MS**<br>Column: C18 (100 mm x 2.1 mm x 1.8 µm) at 45°C<br>MP: (A) H_2O/0.01% AA ; (B) ACN, in gradient conditions<br>Detector: ESI (-), QqQ, SRM	2019	[51]
Pig	Plasma	ZEA-14GlcA, α,β-ZEL-14GlcA, α-ZEL-7GlcA, β-ZEL-16GlcA	1.1-3.1	3.7-10.2	**LLE**<br>150 µL plasma + 450 µL ACN. Centrifuge. Evaporate supernatant (N_2) and reconstitute with 100 µL ACN/H_2O (50/50, v/v)	**LC-HRMS**<br>Column: C18 (100 mm x 2.1 mm x 2.6 µm) at 45°C<br>MP: (A) H_2O/0.2% FA ; (B) ACN/0.2%FA, in gradient conditions<br>Detector: ESI (-), QOrbitrap	2019	[51]
Pig	Urine	DON, DOM-1, ZEA, α-ZEL, β-ZEL, FB1, OTA, AFM1	0.006-0.36	0.02-1.21	**IAC**<br>5 mL urine + β-glucuronidase/sulfatase + water (1:1, v/v) + Myco6in1+TM multi-antibody IAC and OASIS HLB® column in tandem. Dry and reconstitute with 200 µL of MP (MeOH/H_2O, 20/80, v/v)	**LC-MS/MS**<br>Column: C18 (100 mm x 2.1 mm x 1.7 µm) at 40°C<br>Flow: 0.25 mL/min<br>MP: (A) MeOH/0.5% AA; (B) H_2O/0.5%AA, in gradient conditions<br>Detector: ESI (-), QqQ, MRM	2019	[52]
Poultry	Feces/excreta and ileal content	AFB1, AF2, AFG1, AFG2, AFM1, AFP1, AFQ1, AFB1-N^7-guanine	0.045-0.135	0.15-0.45	**LLE-SPE**<br>1 g + AF standard mixture (to 10ng/g). 500 mg + 5 mL ACN/H_2O/AA (79:20:1) + rotary shaker (40 min) + SPE cartridge (Stata C18-T). Elute with MeOH/H_2O/AA. Evaporate and reconstitute with 0.25 mL MeOH/H_2O/AA (50:49.9:0.1)	**LC-MS/MS**<br>Column: C18 (15 mm x 3.1 mm x 2.6µm)<br>Flow: 0.4 mL/min<br>MP: (A) H_2O/AA (99.9/0.1); (B) MeOH/H_2O/AA(98/1.9/0.1, v/v/v) both with 5 mM ammonium acetate in gradient conditions<br>Detector: ESI (+), QTrap, MRM	2019	[53]

Table S3. Analytical methods employed for the analysis of mycotoxin biomarkers in animal biological fluids (continuation)

Animal	Matrix	Analyte/s	LOD (ng/g or ng/mL)	LOQ (ng/g or ng/mL)	Sample preparation	Separation and detection Technique	Year	Ref.
Pig	Heart, Liver, Spleen, Muscle	ZEN, ZAN, β-ZAL, α-ZAL, β-ZEL, α-ZEL	0.5-1	1-2	**QuEChERS**<br>5 g + 5 mL H_2O + 5 mL ACN/0.1% FA + 2 g of $MgSO_4$ and 0.5 g of NaCl + 50 mg C18 + 100 mg $MgSO_4$. Shake and centrifuge + evaporate (N_2) and reconstitute with 400 µL ACN/H_2O (50/50, v/v) + 400 µL hexane	**LC-MS/MS**<br>Column: C18 (100 mm x 4.6 mm x 3.5 µm) at 30ºC<br>Flow: 0.35 mL/min<br>MP: (A) 5 mH ammonium acetate; (B) MeOH, in gradient conditions<br>Detector: ESI (-), QqQ, SRM	2018	[54]
Goat	Plasma<br>Feces<br>Urine	FUS-X, NIV	0.5-1	1-2	**LLE-SPE**<br>1 mL plasma or urine, or 5 g feces + 3 mL ACN/H_2O (3/1) + ammonium sulfate. Shake and centrifuge + C18 Sep-pak silica cartridge. Evaporate (N_2) and reconstitute with 500 µL MeOH/H_2O + 5mM ammonium acetate	**LC-MS/MS**<br>Column: C18 (50 mm x 4.6 mm x 1.8 µm) at 40ºC<br>Flow: 0.4 mL/min<br>MP: (A) 5 mH ammonium acetate; (B) MeOH, in gradient conditions<br>Detector: ESI (+), QqQ, SRM	2018	[55]
Pig	Plasma	FB1, FB2, FB3, pHFB1, HFB1, pHFB2, HFB2, FB3, pHFB3, HFB3	0.05-0.27	0.15-0.8	**LLE**<br>300 µL plasma + 900 µL MeOH/ACN (50/50, v/v). Centrifuge. Reextract with 200 µL ACN/H_2O/FA (50/49/1, v/v/v). Evaporate (N_2) and reconstitute with 300 µL ACN/H_2O/FA (50/49/1, v/v/v)	**LC-MS/MS**<br>Column: C18 (150 mm x 3 mm x 3 µm) at 35ºC<br>MP: (A) H_2O/0.1% AA; (B) ACN/0.1% AA, in gradient conditions<br>Detector: ESI (-), QTrap, SRM	2018	[56]
	Feces		10-217	21-725	1 g of homogenized feces samples + 10 or 5 mL of ACN/H_2O/FA (74/25/1, v/v/v). Shake. Centrifuge and dilute with extraction solvent			
	Urine		0.18-0.36	0.6-4.5	150 µL aliquots of urine (diluted with water to 4 mM creatinine content) + 10 µL IS + 290 µL MeOH/FA (99/1, v/v). Shake and centrifuge			

Table S3. Analytical methods employed for the analysis of mycotoxin biomarkers in animal biological fluids (continuation)

Animal	Matrix	Analyte/s	LOD (ng/g or ng/mL)	LOQ (ng/g or ng/mL)	Sample preparation	Separation and detection Technique	Year	Ref.
Poultry	Plasma	FB1, pHFB1, HFB1, FB2	0.03-0.17	0.72-2.5	**SPE**<br>100 µL plasma + Oasis® Ostro 96-well plate + 300 µL ACN/1% FA	**LC-MS/MS**<br>Column: C18 (100 mm x 2.1 mm x 1.8 µm)<br>MP: (A) H_2O/10 mM ammonium formate, 0.3% FA; (B) ACN, in gradient conditions<br>Detector: ESI (+), QqQ, MRM	2018	[57]
Poultry	Liver and gizzards	AFB1	0.02-0.05	0.08-0.15	**LLE**<br>2 g + 8 mL MeOH. Shake and centrifuge. Dilute 300 µL supernatant with 900 µL MeOH/extraction buffer	**ELISA**<br>AFB1 MaxSignal® commercial kit	2017	[58]
Poultry	Plasma<br>Feces<br>Urine	ZEA, α-ZEL, β-ZEL, Phase II metabolites	n.i.	n.i.	**SPE and (LLE + SEP, combined method)**<br>plasma or urine sample + Oasis HLB Cartridge + MeOH/H_2O (5/95, v/v). Evaporate and redissolve in in ACN/H_2O (15/85, v/v)<br>2 g of homogenized feces samples + 10 mL EtOAc. Shake. Centrifuge. Evaporate (N_2) and redissolve in ACN/H_2O (15/85, v/v) +	**LC-MS/MS**<br>Column: C18 (100 mm x 2.1 mm x 1.7 µm)<br>Flow: 0.3 mL/min<br>MP: (A) H_2O/0.1% FA; (B) ACN/0.1% FA, in gradient conditions<br>Detector: ESI (-), QTOF, MRM	2017	[59]

Abbreviations:

15-ADON	15-acetyldeoxynivalenol
3-ADON	3-acetyldeoxynivalenol
AA	Acetic acid
ABM	Animal Biomonitoring
ACN	Acetonitrile
AFB1	Aflatoxin B1
AFB2	Aflatoxin B2
AFBO	Aflatoxin-8,9-epoxide
AFG1	Aflatoxin G1
AFG2	Aflatoxin G2
AFL	Aflatoxicol
AFM1	Aflatoxin M1
AFP1	Aflatoxin P1
AFQ1	Aflatoxin Q1
Afs	Aflatoxins
AME	Alternariol Monomethyl Ether
AOH	Alternariol
ATX-I	Altertoxine I
BEA	Beauvericin
CIT	Citrinin
DAS	Diacetoxyscirpenol
DBS	Dried blood spot
DH-CIT	Dihydrocitrinone
DOM	Deepoxydeoxynivalenol
DOM-1	Deepoxidesoxynivalenol
DON	Deoxynivalenol
DON-15GlcA	DON-15-glucuronide
DON-3GlcA	DON-3-glucuronide
DON-3gluc	DON-3 glucoside
DON-s	DON sulphate
EFSA	European Food Safety Authority
ELISA	Enzyme-Linked ImmunoSorbent Assay
ENNA	Enniatin A
ENNA1	Enniatin A1
ENNB	Enniatin B
ENNB1	Enniatin B1
ERGOT	Ergot Alkaloids
ESI	Electrospray ionization
EtOAc	Ethyl acetate
EU	European Union
FA	Formic acid
FB1	Fumonisin B1
FB2	Fumonisin B2
FB3	Fumonisin B3
FB4	Fumonisin B3
FBs	Fumonisins
FLD	Fluorescence Detector
FUS-X	Fusarenon-X
GlcA	Glucuronide
HBM	Human Biomonitorin
HFBx	Hydrolized FBx
HT-2	HT-2 toxin
IAC	Immunoaffinity Column
IARC	International Agency for Resarch on Cancer
IV	Intravenous administration

LC	Liquid Chromatography
LLE	Liquid-liquid extraction
LOD	Limit of detection
LOQ	Limit of Quantification
MeOH	Methanol
MON	Moniliformin
MP	Mobile phase
MRM	Multiple Reaction Monitoring
MS	Mass Spectrometer
MS/MS	Tanden Mass Spectrometry
n.i	not indicated
NEO	Neosolaniol
NIV	Nivalenol
OTA	Ochratoxin A
OTα	Ochratoxin α
OTB	Ochratoxin B
PAT	Patulin
pHFBx	Partially hydrolized FBx
PO	Oral administration
PSA	Primary Secondary Amines
Qorbitap	Quadrupole-orbitrap
QqQ	Triple quadrupole;
QTOF	Quadrupole-time of flight
Qtrap	Quadrupole- ion trap
QuEChERs	Quick, easy, cheap, effective, rugged, and safe
ROQC	Roquefortine C
SLE	Solid Liquid Extraction
SPE	Solid Phase Extraction
SRM	Selective reaction monitoring
STER	Sterigmatocystin
T-2	T-2 toxin
TENT	Tentoxin
ZAL	Zearalanol
ZAN	Zearalanone
ZAN-14-ClcA	Zearalanone-14-glucuronide
ZEA	Zearalenone
ZEL-14-ClcA	Zearalenone-14-glucuronide
ZEL-16-ClcA	Zearalenone-16-glucuronide
α-ZAL	α-zearalanol
α-ZEL	α-zearalenol
β-ZAL	β-zearalanol
β-ZEL	β-zearalenol

References

1. Yang, C.-K.; Cheng, Y.-H.; Tsai, W.-T.; Liao, R.-W.; Chang, C.-S.; Chien, W.-C.; Jhang, J.-C.; Yu, Y.-H. Prevalence of Mycotoxins in Feed and Feed Ingredients between 2015 and 2017 in Taiwan. *Environ. Sci. Pollut. Res.* **2019**, *26*, 23798–23806, doi:10.1007/s11356-019-05659-0.
2. Nishimwe, K.; Bowers, E.; Ayabagabo, J. de D.; Habimana, R.; Mutiga, S.; Maier, D. Assessment of Aflatoxin and Fumonisin Contamination and Associated Risk Factors in Feed and Feed Ingredients in Rwanda. *Toxins (Basel).* **2019**, *11*, 270, doi:10.3390/toxins11050270.
3. Franco, L.T.; Petta, T.; Rottinghaus, G.E.; Bordin, K.; Gomes, G.A.; Oliveira, C.A.F. Co-Occurrence of Mycotoxins in Maize Food and Maize-Based Feed from Small-Scale Farms in Brazil: A Pilot Study. *Mycotoxin Res.* **2019**, *35*, 65–73, doi:10.1007/s12550-018-0331-4.
4. Mokubedi, S.M.; Phoku, J.Z.; Changwa, R.N.; Gbashi, S.; Njobeh, P.B. Analysis of Mycotoxins Contamination in Poultry Feeds Manufactured in Selected Provinces of South Africa Using UHPLC-MS/MS. *Toxins (Basel).* **2019**, *11*, 452, doi:10.3390/toxins11080452.
5. Juan, C.; Oueslati, S.; Mañes, J.; Berrada, H. Multimycotoxin Determination in Tunisian Farm Animal Feed.

J. Food Sci. **2019**, *84*, 3885–3893, doi:10.1111/1750-3841.14948.
6. Abdallah; Girgin; Baydar Mycotoxin Detection in Maize, Commercial Feed, and Raw Dairy Milk Samples from Assiut City, Egypt. *Vet. Sci.* **2019**, *6*, 57, doi:10.3390/vetsci6020057.
7. Arroyo-Manzanares, N.; Rodríguez-Estévez, V.; Arenas-Fernández, P.; García-Campaña, A.M.; Gámiz-Gracia, L. Occurrence of Mycotoxins in Swine Feeding from Spain. *Toxins (Basel).* **2019**, *11*, 342, doi:10.3390/toxins11060342.
8. Akinmusire, O.O.; El-Yuguda, A.-D.; Musa, J.A.; Oyedele, O.A.; Sulyok, M.; Somorin, Y.M.; Ezekiel, C.N.; Krska, R. Mycotoxins in Poultry Feed and Feed Ingredients in Nigeria. *Mycotoxin Res.* **2019**, *35*, 149–155, doi:10.1007/s12550-018-0337-y.
9. Beyene, A.M.; Du, X.; E. Schrunk, D.; Ensley, S.; Rumbeiha, W.K. High-Performance Liquid Chromatography and Enzyme-Linked Immunosorbent Assay Techniques for Detection and Quantification of Aflatoxin B1 in Feed Samples: A Comparative Study. *BMC Res. Notes* **2019**, *12*, 492, doi:10.1186/s13104-019-4538-z.
10. Gao, J.; Wang, J.; Wu, C.; Hou, F.; Chang, S.; Wang, Z.; Pu, Q.; Guo, D.; Fu, H. Fast Screening of Aflatoxins in Dairy Cattle Feeds with CE-LIF Method Combined with Preconcentration Technique of Vortex Assisted Low Density Solvent–Microextraction. *Electrophoresis* **2019**, *40*, 499–507, doi:10.1002/elps.201800339.
11. Jedziniak, P.; Panasiuk, Ł.; Pietruszka, K.; Posyniak, A. Multiple Mycotoxins Analysis in Animal Feed with LC-MS/MS: Comparison of Extract Dilution and Immunoaffinity Clean-up. *J. Sep. Sci.* **2019**, *42*, 1240–1247, doi:10.1002/jssc.201801113.
12. Bani Ismail, Z.; Al-Nabulsi, F.; Abu-Basha, E.; Hananeh, W. Occurrence of On-Farm Risk Factors and Health Effects of Mycotoxins in Dairy Farms in Jordan. *Trop. Anim. Health Prod.* **2020**, *52*, 2371–2377, doi:10.1007/s11250-019-02166-9.
13. Nualkaw, K.; Poapolathep, S.; Zhang, Z.; Zhang, Q.; Giorgi, M.; Li, P.; Logrieco, A.F.; Poapolathep, A. Simultaneous Determination of Multiple Mycotoxins in Swine, Poultry and Dairy Feeds Using Ultra High Performance Liquid Chromatography-Tandem Mass Spectrometry. *Toxins (Basel).* **2020**, *12*, 253, doi:10.3390/toxins12040253.
14. Kemboi, D.C.; Ochieng, P.E.; Antonissen, G.; Croubels, S.; Scippo, M.-L.; Okoth, S.; Kangethe, E.K.; Faas, J.; Doupovec, B.; Lindahl, J.F.; et al. Multi-Mycotoxin Occurrence in Dairy Cattle and Poultry Feeds and Feed Ingredients from Machakos Town, Kenya. *Toxins (Basel).* **2020**, *12*, 762, doi:10.3390/toxins12120762.
15. Rodríguez-Blanco, M.; Ramos, A.J.; Prim, M.; Sanchis, V.; Marín, S. Usefulness of the Analytical Control of Aflatoxins in Feedstuffs for Dairy Cows for the Prevention of Aflatoxin M1 in Milk. *Mycotoxin Res.* **2020**, *36*, 11–22, doi:10.1007/s12550-019-00362-y.
16. Kumar, A.; Dhanshetty, M.; Banerjee, K. Development and Validation of a Method for Direct Analysis of Aflatoxins in Animal Feeds by Ultra-High-Performance Liquid Chromatography with Fluorescence Detection. *J. AOAC Int.* **2020**, *103*, 940–945, doi:10.1093/jaoacint/qsz037.
17. Muñoz-Solano, B.; González-Peñas, E. Mycotoxin Determination in Animal Feed: An LC-FLD Method for Simultaneous Quantification of Aflatoxins, Ochratoxins and Zearelanone in This Matrix. *Toxins (Basel).* **2020**, *12*, 374, doi:10.3390/toxins12060374.
18. Vaičiulienė, G.; Bakutis, B.; Jovaišienė, J.; Falkauskas, R.; Gerulis, G.; Kerzienė, S.; Baliukonienė, V. Prevalence of Mycotoxins and Endotoxins in Total Mixed Rations and Different Types of Ensiled Forages for Dairy Cows in Lithuania. *Toxins (Basel).* **2021**, *13*, 890, doi:10.3390/toxins13120890.
19. Patyal, A.; Gill, J.P.S.; Bedi, J.S.; Aulakh, R.S. Assessment of Aflatoxin Contamination in Dairy Animal Concentrate Feed from Punjab, India. *Environ. Sci. Pollut. Res.* **2021**, *28*, 37705–37715, doi:10.1007/s11356-021-13321-x.
20. Awapak, D.; Petchkongkaew, A.; Sulyok, M.; Krska, R. Co-Occurrence and Toxicological Relevance of Secondary Metabolites in Dairy Cow Feed from Thailand. *Food Addit. Contam. Part A* **2021**, *38*, 1013–1027, doi:10.1080/19440049.2021.1905186.
21. Bervis, N.; Lorán, S.; Juan, T.; Carramiñana, J.J.; Herrera, A.; Ariño, A.; Herrera, M. Field Monitoring of Aflatoxins in Feed and Milk of High-Yielding Dairy Cows under Two Feeding Systems. *Toxins (Basel).* **2021**, *13*, 201, doi:10.3390/toxins13030201.
22. Nasaruddin, N.; Jinap, S.; Samsudin, N.I.P.; Kamarulzaman, N.H.; Sanny, M. Prevalence of Mycotoxigenic Fungi and Assessment of Aflatoxin Contamination: A Multiple Case Study along the Integrated Corn-based Poultry Feed Supply Chain in Malaysia. *J. Sci. Food Agric.* **2021**, *101*, 1812–1821, doi:10.1002/jsfa.10795.
23. Zhao, L.; Zhang, L.; Xu, Z.; Liu, X.; Chen, L.; Dai, J.; Karrow, N.A.; Sun, L. Occurrence of Aflatoxin B1, Deoxynivalenol and Zearalenone in Feeds in China during 2018–2020. *J. Anim. Sci. Biotechnol.* **2021**, *12*, 74, doi:10.1186/s40104-021-00603-0.
24. Changwa, R.; De Boevre, M.; De Saeger, S.; Njobeh, P.B. Feed-Based Multi-Mycotoxin Occurrence in Smallholder Dairy Farming Systems of South Africa: The Case of Limpopo and Free State. *Toxins (Basel).* **2021**, *13*, 166, doi:10.3390/toxins13020166.
25. Seo, H.; Jang, S.; Jo, H.; Kim, H.; Lee, S.; Yun, H.; Jeong, M.; Moon, J.; Na, T.; Cho, H. Optimization of the

QuEChERS-Based Analytical Method for Investigation of 11 Mycotoxin Residues in Feed Ingredients and Compound Feeds. *Toxins (Basel).* **2021**, *13*, 767, doi:10.3390/toxins13110767.
26. Naveed, M.; Haleem, K.S.; Ghazanfar, S.; Tauseef, I.; Bano, N.; Adetunji, C.O.; Saleem, M.H.; Alshaya, H.; Paray, B.A. Quantitative Estimation of Aflatoxin Level in Poultry Feed in Selected Poultry Farms. *Biomed Res. Int.* **2022**, *2022*, 1–7, doi:10.1155/2022/5397561.
27. Kassaw, T.S.; Megerssa, Y.C.; Woldemariyam, F.T. Occurrence of Aflatoxins in Poultry Feed in Selected Chicken Rearing Villages of Bishoftu Ethiopia. *Vet. Med. Res. Reports* **2022**, *Volume 13*, 277–286, doi:10.2147/VMRR.S384148.
28. Ferrari, L.; Fumagalli, F.; Rizzi, N.; Grandi, E.; Vailati, S.; Manoni, M.; Ottoboni, M.; Cheli, F.; Pinotti, L. An Eight-Year Survey on Aflatoxin B1 Indicates High Feed Safety in Animal Feed and Forages in Northern Italy. *Toxins (Basel).* **2022**, *14*, 763, doi:10.3390/toxins14110763.
29. Hao, W.; Li, A.; Wang, J.; An, G.; Guan, S. Mycotoxin Contamination of Feeds and Raw Materials in China in Year 2021. *Front. Vet. Sci.* **2022**, *9*, 1–11, doi:10.3389/fvets.2022.929904.
30. Biscoto, G.L.; Salvato, L.A.; Alvarenga, É.R.; Dias, R.R.S.; Pinheiro, G.R.G.; Rodrigues, M.P.; Pinto, P.N.; Freitas, R.P.; Keller, K.M. Mycotoxins in Cattle Feed and Feed Ingredients in Brazil: A Five-Year Survey. *Toxins (Basel).* **2022**, *14*, 552, doi:10.3390/toxins14080552.
31. Li, A.; Hao, W.; Guan, S.; Wang, J.; An, G. Mycotoxin Contamination in Feeds and Feed Materials in China in Year 2020. *Front. Vet. Sci.* **2022**, *9*, doi:10.3389/fvets.2022.1016528.
32. Liu, Y.; Jin, Y.; Guo, Q.; Wang, X.; Luo, S.; Yang, W.; Li, J.; Chen, Y. Immunoaffinity Cleanup and Isotope Dilution-Based Liquid Chromatography Tandem Mass Spectrometry for the Determination of Six Major Mycotoxins in Feed and Feedstuff. *Toxins (Basel).* **2022**, *14*, 631, doi:10.3390/toxins14090631.
33. Mackay, N.; Marley, E.; Leeman, D.; Poplawski, C.; Donnelly, C. Analysis of Aflatoxins, Fumonisins, Deoxynivalenol, Ochratoxin A, Zearalenone, HT-2, and T-2 Toxins in Animal Feed by LC–MS/MS Using Cleanup with a Multi-Antibody Immunoaffinity Column. *J. AOAC Int.* **2022**, *105*, 1330–1340, doi:10.1093/jaoacint/qsac035.
34. Wang, Y.; Wang, X.; Wang, S.; Fotina, H.; Wang, Z. A Novel Lateral Flow Immunochromatographic Assay for Rapid and Simultaneous Detection of Aflatoxin B1 and Zearalenone in Food and Feed Samples Based on Highly Sensitive and Specific Monoclonal Antibodies. *Toxins (Basel).* **2022**, *14*, 615, doi:10.3390/toxins14090615.
35. Muñoz-Solano, B.; González-Peñas, E. Co-Occurrence of Mycotoxins in Feed for Cattle, Pigs, Poultry, and Sheep in Navarra, a Region of Northern Spain. *Toxins (Basel).* **2023**, *15*, 172, doi:10.3390/toxins15030172.
36. Hao, W.; Guan, S.; Li, A.; Wang, J.; An, G.; Hofstetter, U.; Schatzmayr, G. Mycotoxin Occurrence in Feeds and Raw Materials in China: A Five-Year Investigation. *Toxins (Basel).* **2023**, *15*, 63, doi:10.3390/toxins15010063.
37. Prasad, S.; Streit, B.; Gruber, C.; Gonaus, C. Enzymatic Degradation of Ochratoxin A in the Gastrointestinal Tract of Piglets. *J. Anim. Sci.* **2023**, *101*, 1–11, doi:10.1093/jas/skad171.
38. Muñoz-Solano, B.; González-Peñas, E. Biomonitoring of 19 Mycotoxins in Plasma from Food-Producing Animals (Cattle, Poultry, Pigs, and Sheep). *Toxins (Basel).* **2023**, *15*, 295, doi:10.3390/toxins15040295.
39. De Baere, S.; Ochieng, P.E.; Kemboi, D.C.; Scippo, M.-L.; Okoth, S.; Lindahl, J.F.; Gathumbi, J.K.; Antonissen, G.; Croubels, S. Development of High-Throughput Sample Preparation Procedures for the Quantitative Determination of Aflatoxins in Biological Matrices of Chickens and Cattle Using UHPLC-MS/MS. *Toxins (Basel).* **2023**, *15*, 37, doi:10.3390/toxins15010037.
40. Panisson, J.C.; Wellington, M.O.; Bosompem, M.A.; Nagl, V.; Schwartz-Zimmermann, H.E.; Columbus, D.A. Urinary and Serum Concentration of Deoxynivalenol (DON) and DON Metabolites as an Indicator of DON Contamination in Swine Diets. *Toxins (Basel).* **2023**, *15*, 120, doi:10.3390/toxins15020120.
41. Streit, B.; Czabany, T.; Weingart, G.; Marchetti-Deschmann, M.; Prasad, S. Toolbox for the Extraction and Quantification of Ochratoxin A and Ochratoxin Alpha Applicable for Different Pig and Poultry Matrices. *Toxins (Basel).* **2022**, *14*, 432, doi:10.3390/toxins14070432.
42. Zhang, S.; Zhou, S.; Yu, S.; Zhao, Y.; Wu, Y.; Wu, A. LC-MS/MS Analysis of Fumonisin B1, B2, B3, and Their Hydrolyzed Metabolites in Broiler Chicken Feed and Excreta. *Toxins (Basel).* **2022**, *14*, 131, doi:10.3390/toxins14020131.
43. Tkaczyk, A.; Jedziniak, P. Development of a Multi-Mycotoxin LC-MS/MS Method for the Determination of Biomarkers in Pig Urine. *Mycotoxin Res.* **2021**, *37*, 169–181, doi:10.1007/s12550-021-00428-w.
44. Tkaczyk, A.; Jedziniak, P.; Zielonka, Ł.; Dąbrowski, M.; Ochodzki, P.; Rudawska, A. Biomarkers of Deoxynivalenol, Citrinin, Ochratoxin A and Zearalenone in Pigs after Exposure to Naturally Contaminated Feed Close to Guidance Values. *Toxins (Basel).* **2021**, *13*, 750, doi:10.3390/toxins13110750.
45. Meerpoel, C.; Vidal, A.; Tangni, E.K.; Huybrechts, B.; Couck, L.; De Rycke, R.; De Bels, L.; De Saeger, S.; Van den Broeck, W.; Devreese, M.; et al. A Study of Carry-Over and Histopathological Effects after Chronic Dietary Intake of Citrinin in Pigs, Broiler Chickens and Laying Hens. *Toxins (Basel).* **2020**, *12*, 719, doi:10.3390/toxins12110719.

46. Meerpoel, C.; Vidal, A.; Huybrechts, B.; Tangni, E.K.; De Saeger, S.; Croubels, S.; Devreese, M. Comprehensive Toxicokinetic Analysis Reveals Major Interspecies Differences in Absorption, Distribution and Elimination of Citrinin in Pigs and Broiler Chickens. *Food Chem. Toxicol.* **2020**, *141*, 111365, doi:10.1016/j.fct.2020.111365.
47. Antonissen, G.; De Baere, S.; Novak, B.; Schatzmayr, D.; den Hollander, D.; Devreese, M.; Croubels, S. Toxicokinetics of Hydrolyzed Fumonisin B1 after Single Oral or Intravenous Bolus to Broiler Chickens Fed a Control or a Fumonisins-Contaminated Diet. *Toxins (Basel).* **2020**, *12*, 413, doi:10.3390/toxins12060413.
48. Lauwers, M.; De Baere, S.; Letor, B.; Rychlik, M.; Croubels, S.; Devreese, M. Multi LC-MS/MS and LC-HRMS Methods for Determination of 24 Mycotoxins Including Major Phase I and II Biomarker Metabolites in Biological Matrices from Pigs and Broiler Chickens. *Toxins (Basel).* **2019**, *11*, 171, doi:10.3390/toxins11030171.
49. Lauwers, M.; Croubels, S.; Letor, B.; Gougoulias, C.; Devreese, M. Biomarkers for Exposure as A Tool for Efficacy Testing of A Mycotoxin Detoxifier in Broiler Chickens and Pigs. *Toxins (Basel).* **2019**, *11*, 187, doi:10.3390/toxins11040187.
50. Lauwers, M.; Croubels, S.; De Baere, S.; Sevastiyanova, M.; Romera Sierra, E.M.; Letor, B.; Gougoulias, C.; Devreese, M. Assessment of Dried Blood Spots for Multi-Mycotoxin Biomarker Analysis in Pigs and Broiler Chickens. *Toxins (Basel).* **2019**, *11*, 541, doi:10.3390/toxins11090541.
51. Catteuw, A.; Broekaert, N.; De Baere, S.; Lauwers, M.; Gasthuys, E.; Huybrechts, B.; Callebaut, A.; Ivanova, L.; Uhlig, S.; De Boevre, M.; et al. Insights into In Vivo Absolute Oral Bioavailability, Biotransformation, and Toxicokinetics of Zearalenone, α-Zearalenol, β-Zearalenol, Zearalenone-14-Glucoside, and Zearalenone-14-Sulfate in Pigs. *J. Agric. Food Chem.* **2019**, *67*, 3448–3458, doi:10.1021/acs.jafc.8b05838.
52. Gambacorta, L.; Olsen, M.; Solfrizzo, M. Pig Urinary Concentration of Mycotoxins and Metabolites Reflects Regional Differences, Mycotoxin Intake and Feed Contaminations. *Toxins (Basel).* **2019**, *11*, 378, doi:10.3390/toxins11070378.
53. Jurišić, N.; Schwartz-Zimmermann, H.E.; Kunz-Vekiru, E.; Moll, W.D.; Schweiger, W.; Fowler, J.; Berthiller, F. Determination of Aflatoxin Biomarkers in Excreta and Ileal Content of Chickens. *Poult. Sci.* **2019**, *98*, 5551–5561, doi:10.3382/ps/pez308.
54. Yan, Z.; Wang, L.; Wang, J.; Tan, Y.; Yu, D.; Chang, X.; Fan, Y.; Zhao, D.; Wang, C.; De Boevre, M.; et al. A QuEChERS-Based Liquid Chromatography-Tandem Mass Spectrometry Method for the Simultaneous Determination of Nine Zearalenone-Like Mycotoxins in Pigs. *Toxins (Basel).* **2018**, *10*, 129, doi:10.3390/toxins10030129.
55. Phruksawan, W.; Poapolathep, S.; Giorgi, M.; Imsilp, K.; Sakulthaew, C.; Owen, H.; Poapolathep, A. Toxicokinetic Profile of Fusarenon-X and Its Metabolite Nivalenol in the Goat (Capra Hircus). *Toxicon* **2018**, *153*, 78–84, doi:10.1016/j.toxicon.2018.08.015.
56. Schertz, H.; Kluess, J.; Frahm, J.; Schatzmayr, D.; Dohnal, I.; Bichl, G.; Schwartz-Zimmermann, H.; Breves, G.; Dänicke, S. Oral and Intravenous Fumonisin Exposure in Pigs—A Single-Dose Treatment Experiment Evaluating Toxicokinetics and Detoxification. *Toxins (Basel).* **2018**, *10*, 150, doi:10.3390/toxins10040150.
57. De Baere, S.; Croubels, S.; Novak, B.; Bichl, G.; Antonissen, G. Development and Validation of a UPLC-MS/MS and UPLC-HR-MS Method for the Determination of Fumonisin B1 and Its Hydrolysed Metabolites and Fumonisin B2 in Broiler Chicken Plasma. *Toxins (Basel).* **2018**, *10*, 62, doi:10.3390/toxins10020062.
58. Sineque, A.; Macuamule, C.; Dos Anjos, F. Aflatoxin B1 Contamination in Chicken Livers and Gizzards from Industrial and Small Abattoirs, Measured by ELISA Technique in Maputo, Mozambique. *Int. J. Environ. Res. Public Health* **2017**, *14*, 951, doi:10.3390/ijerph14090951.
59. Yang, S.; Zhang, H.; Sun, F.; De Ruyck, K.; Zhang, J.; Jin, Y.; Li, Y.; Wang, Z.; Zhang, S.; De Saeger, S.; et al. Metabolic Profile of Zearalenone in Liver Microsomes from Different Species and Its in Vivo Metabolism in Rats and Chickens Using Ultra High-Pressure Liquid Chromatography-Quadrupole/Time-of-Flight Mass Spectrometry. *J. Agric. Food Chem.* **2017**, *65*, 11292–11303, doi:10.1021/acs.jafc.7b04663.
